# Taxation of foods high in fat, sugar, and sodium in India: A modelling study of health and economic impacts

**DOI:** 10.1371/journal.pmed.1004572

**Published:** 2026-01-05

**Authors:** Maxime Roche, Jingmin Zhu, Jack Olney, Daniel J. Laydon, William Joe, Manika Sharma, Lindsay Steele, Franco Sassi

**Affiliations:** 1 Centre for Health Economics & Policy Innovation, Department of Economics & Public Policy, Imperial College Business School, South Kensington Campus, London, United Kingdom; 2 Institute of Economic Growth, Delhi University Enclave, North Campus, New Delhi, India; 3 Resolve to Save Lives Services PLC, New Delhi, India; 4 Resolve to Save Lives, Alexandria, Virginia, United States of America; Carolina Population Center, UNITED STATES OF AMERICA

## Abstract

**Background:**

Consumption of foods high in fat, sugar, and sodium (HFSS) and obesity are rapidly increasing in India. Taxing HFSS foods has been proposed as one of the policy interventions to promote healthier diets globally. This study estimates the effect of this approach on nutrient intake, diet-related disease, and associated health and economic burdens in India.

**Methods and findings:**

We use a nationally representative expenditure survey of 261,746 households, dietary requirements, and food composition tables to model individual nutrient intake. Consumer responsiveness to food price changes for three income terciles, captured in price elasticities, is estimated using an Almost Ideal Demand System model. Longer-term policy impacts are estimated through a novel dynamic microsimulation model, Health-GPS. Modelled policy outcomes include changes in risk exposures, disease incidence and burden, and total health expenditure. On average, 9.9% of total energy intake comes from HFSS items, based on the definition by the Food Safety and Standards Authority of India’s Labelling and Display Amendment Draft Regulations 2022. Applying the highest Goods and Services Tax (GST) rate of 40% on HFSS items is associated with a persistent average per capita decrease of 0.1705 kg/m^2^ (95% CI: −0.1709, −0.1700) in body mass index and 45.8 mg (95% CI: −45.9, −45.7) in daily sodium intake. Over 30 years, this could reduce annual disease incidence by up to 1.72% (95% CI: −1.78%, −1.66%) on average and prevent 0.63 million (95% CI: −0.71, −0.55) disability-adjusted life years per year from ischaemic heart disease, chronic kidney disease, stroke, diabetes, and asthma, reducing total health expenditure by US$601 million (95% CI: −624, −578) per year. Larger absolute health gains accrue to higher-income individuals, reflecting higher baseline HFSS food intake. Given substitution patterns and a price-inelastic demand, the tax change is expected to generate a 92.0% (95% CI: 88.2%, 95.7%) increase in tax revenue from foods and beverages with only a minor effect on household spending (+1.0%, 95% CI: + 0.0%, + 1.9%). This analysis only captures the potential health impacts of changes in energy and sodium intakes. In addition, it does not model underlying temporal trends in disease incidence beyond those due to demographic changes, which would make our health impact estimates conservative if baseline disease risks were to increase in the future.

**Conclusions:**

Higher taxation of HFSS foods could help mitigate rising incidence of diet-related diseases and morbidity in India, reduce healthcare costs, and serve as an additional source of revenue for the government.

## Author summary

**Why was this study done?:**

- India faces a rising burden of obesity and diet-related diseases.
- A growing number of countries have implemented or are considering implementing taxes on food high in fat, sugar, and sodium (HFSS).
- However, the long-term population health and economic impact of taxing HFSS items is not well understood, particularly in middle-income economies.

**What did the researchers do and find?:**

- We used the most recent household consumption expenditure data to estimate consumers response to food price changes, nutrient intake, and model the potential impact across the Indian population of two main scenarios increasing the Goods and Services Tax (GST) rate on HFSS items to: (1) the highest 28% GST rate; (2) with an additional 12% top-up to match the 40% “sin” goods GST rate.
- The simulations indicated that a 40% GST rate on HFSS items could reduce annual average disease incidence by up to 1.72% and avert as many as 19 million disability-adjusted life years (DALYs) over 30 years through improved energy balance and reduced sodium intake, saving up to 0.6% annually in total health spending.
- Given substitution patterns and the limited responsiveness of demand to price changes, the tax changes are expected to generate significant tax revenue increases, up to 92%, while raising household food spending by only approximately 1%, which could be offset by reduced out-of-pocket expenditure.

**What do these findings mean?:**

- This study illustrates potentially large benefits of aligning GST rates with nutritional quality for improving population health, reducing health spending, and providing additional fiscal revenue.
- Further work is needed to incorporate potential benefits of changes in other foods and nutrients, such as fruits, whole grains, or fibre, as well as to assess the tax administration challenges associated with taxing foods based on their nutritional content.
- We assume no underlying time trend in disease incidence, which means our health gains may be underestimated if underlying disease risks were to increase. We also do not capture potential benefits or harms from changes in the consumption of foods and nutrients beyond energy and dietary sodium intake.

## Abstract 3

In a modelling study, Maxime Roche, Jingmin Zhu and colleagues analyse what the effect would be of taxing food items high in fat, sugar and sodium in India, assessing both the effect on population health and the economic impact.

## Introduction

Unhealthy diets and obesity are major risk factors for non-communicable diseases, such as cardiovascular disease, cancer, and diabetes. These diseases account for substantial healthcare costs, as well as wider economic costs for governments and individuals [1]. In India, adult overweight and obesity levels have increased 4-fold since 1975 [2]. Of particular concern is the increasing consumption of highly industrially processed foods rich in energy, saturated fat, sugar, and sodium [3].

Governments can promote healthy diets through fiscal policies, including the taxation of foods high in fat, sugar, and sodium (HFSS), as well as subsidies on fruits and vegetables (F&V), alongside food marketing regulation and front-of-pack nutrition labelling (FOPNL). There are strong health and economic rationales for using fiscal policies to address the external costs of unhealthy diets and obesity (i.e., ‘externalities’, such as collectively borne healthcare costs linked with obesity) while “encouraging people to avoid acting against their own self-interest” (i.e., ‘internalities’) [4,5].

India applies a Goods and Services Tax (GST), introduced in July 2017, at five different rates: 0%, 5%, 12%, 18%, and 28%. Among food and beverage items, the 28% rate only applies to caffeinated and sweetened aerated beverages, which include sugar-sweetened beverages (SSBs), along with an additional 12% compensatory Cess. This cess was part of a nationwide mechanism intended to compensate states for any shortfall in their tax revenue for five years following the introduction of the GST system, relative to a baseline assuming 14% annual growth in their 2015–2016 tax revenue from taxes subsumed under GST. Adjusting existing GST rates to align with nutrition and health objectives may improve diets by shifting food preferences.

Only a few countries have implemented taxes on HFSS foods, with limited scope, targeting either specific food categories (e.g., confectionery and ice cream in Finland or French Polynesia), non-essential energy-dense foods (e.g., in Mexico), or selected nutrients (e.g., sugar and salt in Hungary) [6]. Identifying HFSS foods for taxation based on nutrient content, for example, using a nutrient profile model (NPM), may capture unhealthy foods more broadly, and is less likely to apply high rates to healthier foods or incentivise negative substitutions from ‘healthy’ to ‘less healthy’ foods [7]. NPMs are increasingly used in nutrition policies, such as FOPNL and marketing restrictions. In 2023, Colombia became the first country to introduce a broad excise tax on HFSS foods based on an NPM. The government subsequently increased the excise tax rate in 2024 and 2025, offering a promising example for other countries [8].

This study estimates the potential impacts on nutrient intakes and diet-related diseases of aligning India’s GST rate differentiation on foods and beverages to nutritional quality, placing a higher tax burden on HFSS foods, in line with suggested policy intervention globally [6]. We first estimate baseline energy and nutrient intakes, as well as consumers’ responsiveness to food price changes. We then calibrate a microsimulation model to measure the effects of alternative taxation scenarios on various health and economic outcomes, as well as their distribution across income groups, over the next 30 years.

Simulation analyses in Costa Rica, the Philippines, and the United Kingdom have suggested that NPM-based taxes can significantly improve diet [9–11]. The long-term population health and economic impacts of such a comprehensive nutrient-based approach remain understudied, especially in low- and middle-income countries. A recent study investigated the potential impact of an excise tax on HFSS foods in India on consumption and tax revenue, without considering nutrition or health outcomes [12]. While others have simulated SSB taxes and their impact on non-communicable diseases in middle-income countries [13], including in India [14], our study fills this gap for broader HFSS food taxation.

We contribute to the literature by applying a novel microsimulation model, Health-GPS, to evaluate the impacts of HFSS food taxation in India. The model’s architecture involves a hierarchy of demographic and socio-economic characteristics, risk factors, and diseases [15]. We simulate 0.5% of the Indian population to estimate policy impacts on risk factors, disease incidence, and disability-adjusted life years (DALYs) as well as associated health expenditure. We provide the most recent estimates of macronutrient intakes in the Indian population using the latest National Sample Survey Office (NSSO) Household Consumption Expenditure survey 2022–2023. Lastly, we present updated estimates of the price and income elasticity of demand for foods and beverages by income groups. These represent essential metrics to estimate the potential impact of alternative fiscal reforms.

Our primary objective is to evaluate how higher GST rates on HFSS foods may influence nutrient intakes, body mass index (BMI), diet-related diseases, healthcare expenditure, household spending, and government tax revenue in India. Using Health-GPS, we assess the extent to which aligning GST rates with nutritional quality impacts these outcomes and their distribution across income groups. The findings of this study can inform policymakers in India and elsewhere considering GST reforms and other health taxes on foods and beverages to promote healthier diets and improve population health.

## Methods

### Estimating energy and nutrient intake

Our primary data source is the 2022–2023 Household Consumption Expenditure survey from the NSSO. This survey collected food and beverage consumption, and expenditure from a nationally representative sample of 261,746 households between August 2022 and July 2023 (**Table A1** in S1 Text) provides household characteristics) [16]. Food consumption is recorded over two recall periods: 30 days for cereals, pulses, sugar, and salt, and 7 days for all other items. We harmonised these data to obtain daily household consumption for each item and matched them to their respective nutrient content. We extract nutrient information (energy, sugar, sodium, saturated fat, total fat, carbohydrates, and protein) from the National Institute of Nutrition (NIN) Indian Food Composition Tables 2017 [17], complemented by the United States Department of Agriculture (USDA) Food and Nutrient Database for Dietary Studies 2017–2018 [18]. As NSSO items do not differentiate between processing levels for meat and fish, we assume that 20% of purchased quantities are processed for these groups, based on expert opinion. We individualise household-level intake based on household members’ age and sex using the daily NIN Dietary Guidelines for Indians, 2024 [19]. Lastly, we assume that 55 kg of food is wasted yearly by the average Indian to better proxy dietary intake based on data from the United Nations Environmental Programme [20]. The final sample size is 256,464 households after dropping households with missing food consumption information and 1% lowest outliers (i.e., daily per capita energy intake lower than 933 kcal) and 1% highest outliers (i.e., daily per capita energy intake higher than 4,673 kcal).

### Estimating response to food price changes

We estimate consumer responses to food price changes, accounting for food substitutions (own- and cross-price elasticities of demand) using the 2022–2023 Household Consumption Expenditure survey data and an Almost Ideal Demand System (AIDS) model. In this utility-based structural model, goods are heterogeneous, and households choose the quantity and quality of a given good as a function of its price, the price of other goods, household income, and household sociodemographic characteristics. Following Deaton (1988), we adjust for quality shading and measurement error stemming from the use of unit values as a proxy for unobservable prices (i.e., expenditure divided by quantity) [21]. This model is extensively used to estimate the demand for food in low- and middle-income countries [22]. It assumes spatially varying prices, where all households within a near geographical area, here a cluster, defined by NSSO as a village or urban block, face the same price, regardless of their income group. Any within-cluster variation in unit values is due to differences in the quality of the purchased items. Spatial variations in unit values between clusters provide an identification strategy to avoid the endogeneity of prices and address quality shading.

This paper uses uncompensated price elasticities, which capture the effect of a change in the price of a good on demand, holding income and other prices constant. These elasticities reflect both the substitution effect and the income effect of the price change. Details of the microeconomic foundations of Deaton’s (1988) AIDS model, as well as its derivation and the steps involved in its estimation, have been described elsewhere [23]. More information is provided in Appendix C in S3 Text.

We estimate the model parameters for 11 groups: cereals, dairy, pulses, edible oils and spices (including salt and raw sugar), F&V (including nuts), animal meats (fresh), packaged processed foods, sweets, SSBs, non-SSBs, and a *numeraire* group containing food-away-from-home and non-food expenditure. Adding the latter allows reallocation between food and non-food when prices or real expenditure change. We normalise the *numeraire* group’s unit value to one. After dropping clusters that do not have at least two households consuming at least one item for each group [24], we are left with a total of 5,432 clusters and 212,797 households. We fit this model for three income groups, defined by terciles of monthly per capita consumption expenditure.

### Fiscal policy scenarios

The estimated income-group-specific own- and cross-price elasticities are used to simulate the impacts of two main scenarios consisting of increasing GST to the highest rate of 28% on foods and beverages classified as HFSS based on their nutrient composition, namely the proposed HFSS food definition from the Food Safety and Standards Authority of India (FSSAI) Labelling and Display Amendment Draft Regulations 2022 [25] (scenario 1) as well as an additional 12% (equivalent in effect to a 40% tax rate) (scenario 2). This latter scenario aligns with the ‘sin’ goods GST rate (40%), which is applied to caffeinated and sweetened aerated beverages (including SSBs) and some tobacco products.

The proposed FSSAI definition applies uniform sodium, sugar, and saturated fat thresholds across all processed food and beverage products. We assume processed products to be items not included in Schedule IV, Category III Solid Foods/Liquid Foods, which are exempt from FOPNL under the Indian Nutrition Rating, as per the FSSAI draft regulation [25]. The impact of using an alternative NPM to define HFSS foods, namely the one developed for the South East Asian Region (SEARO) by the World Health Organisation (WHO) [26], based on category-specific nutrient thresholds for energy, sodium, sugar, saturated fat, and total fat content (Table A2 in S1 Text), is tested in further simulations using the same tax rates as in scenarios 1 and 2 (scenarios 3 and 4, respectively). Figs A1 and A2 in S1 Text present the baseline distribution of total energy intake by GST rate and food group and by GST rate and HFSS status (based on the FSSAI definition), respectively. They show that most HFSS items are currently taxed at 12% or 18%.

The immediate impact of each policy scenario on individual energy and nutrient intake, total household expenditure on foods and beverages, and government tax revenue from foods and beverages is calculated based on the estimated price elasticities. Key assumptions, which are subsequently relaxed in sensitivity analyses (Table B1 in S2 Text), include a 100% tax passthrough to retail prices and no supply-side tax-induced product reformulation. Finally, we also test the impact of a 0% GST on F&V and pulses (equivalent to a subsidy). Results sensitivity analyses are presented in Appendix B in S2 Text.

### Health-GPS microsimulation model

Long-term nutrient intake projections and policy impacts are estimated using Health-GPS, a dynamic policy microsimulation model developed by the Centre for Health Economics and Policy Innovation [27]. Health-GPS creates a synthetic population of individuals that broadly reproduces demographic and socioeconomic characteristics of the population of a given country, or sub-national jurisdiction. It simulates individual life histories from birth to death, including age, gender, socioeconomic characteristics, risk factors, and disease profiles. Health-GPS updates annually with statistical and probabilistic models, which are calibrated with real-world data to capture relationships between variables. Changes in disease risks are assumed to result solely from changes in demographic composition and risk factor distributions, with no underlying time trend in diseases.

A baseline scenario is simulated reflecting expected demographic and epidemiological changes over a time horizon in a given population. Intervention scenarios are then simulated, reflecting the introduction of policy interventions applied to targeted risk factors. Changes in risk factor exposures will, in turn, impact other risk factors, risk of diseases, and disease burden. Disease outcomes typically include incidence, prevalence, and mortality of each disease in the population. Disease burden is measured by DALYs, a sum of years of life lost (YLLs) and years lived with disability (YLDs). To calculate YLLs and YLDs, the model iterates over each individual in the simulated population, summing the YLLs due to premature mortality and the YLDs, weighted by the corresponding disability weights. Policy impacts are estimated as the difference in these outcomes between the baseline and intervention scenarios.

In this study, 0.5% of the Indian population is simulated at the individual level, repeated 20 times for each scenario, between 2022 and 2053, with policy introduced in 2024. The synthetic population is modelled to reflect demographic estimates and projections from the United Nations World Population Prospects [28], estimated distribution of individual energy and nutrient intakes, weight and height distributions by age and gender from NCD-RisC [29], as well as prevalence, incidence and excess mortality of diseases from the Institute for Health Metrics and Evaluation Global Burden of Disease [30]. Specifically, for each individual, we simulate two demographic characteristics: age, gender; two socio-economic characteristics: income tertile, sector (urban/rural); six risk factors including four nutrients (carbohydrate, protein, fat, and sodium), physical activity, and BMI; disease status of five key diseases: ischaemic heart disease (IHD), chronic kidney disease (CKD), stroke (including ischaemic stroke, intracerebral haemorrhage and subarachnoid haemorrhage), diabetes, and asthma. A complete list of model parameters and variables definition and sources is presented in Table D1 in S4 Text.

We assume two main diet-related risk-factor-to-disease pathways in this study: the first reflects the impact of diet (and physical activity) on energy balance and thereafter BMI; the second accounts for the impact of dietary sodium. Diet composition-related benefits (e.g., increased consumption of whole grains, F&V, pulses) are not modelled. With data on two risk factors and relative risk ratios linking risk factors to diseases from the literature, changes in incidence and disease burden of five key diseases are estimated. In addition, changes in hypertension prevalence are estimated independently with projected risk factor data from Health-GPS.

We apply two alternative assumptions on the time trend of nutrient intake. First, we assume no time trends in age-, sex- and income-group-specific nutrient intakes, which means that socio-demographic shifts solely drive population-wide changes. Microsimulation outcomes under this assumption thus represent a lower bound of estimates and policy impacts (hereafter referred to as the ‘lower bound’). Our alternative assumption is an income trend which accounts for a constant 6% income growth based on International Monetary Fund projections for real GDP growth [31]. It is scaled by the estimated income group- and food group-specific income elasticities (Table C1 and Fig C1 in S3 Text) and an exponential decay over time, based on Engel’s Law, which posits that, as household income increases, the proportion spent on food decreases [32]. The decay rate is nutrient-specific and estimated by regressing the logarithm of the predicted growth rate in individual nutrient intake on a time trend. The former is estimated based on a regression of individual nutrient intake on monthly per capita total expenditure and a set of household- and individual-level controls among the NSS Household Consumption Expenditure 2022–2023 sample. Fig E1 in S5 Text cross-validates this approach by comparing the estimated yearly growth rate in the consumption of packaged processed foods and sweets—two food groups with a high proportion of HFSS foods (Table A6 in S1 Text)—with historical trends based on Euromonitor International Passport data and Tak and colleagues (2022) for selected highly processed food items [3,33]. This alternative assumption gives an upper-bound estimate of policy impacts (the ‘upper bound’). More details on how we derive the parameters for the income trend assumption can be found in Appendix E in S5 Text.

Policy scenarios are applied at the beginning of 2024 (two years after the initial year) and their impact on health is estimated over 30 years until 2053. We report microsimulations outcomes including the reduction in major risk factors: sodium intake and BMI, cumulative reduction in disease incidence and DALYs, in the general population and by income group. Economic benefits are calculated as averted total health expenditures in United States (US) dollar 2024, including both government and household expenditure. Household health expenditures per disease per year are derived from the literature and converted to total health expenditure based on data from the literature and National Health Accounts Estimates for India 2021–2022 [34]. Confidence intervals are constructed with results from 20 repeated simulation runs to capture stochastic variation.

**Fig 1:**
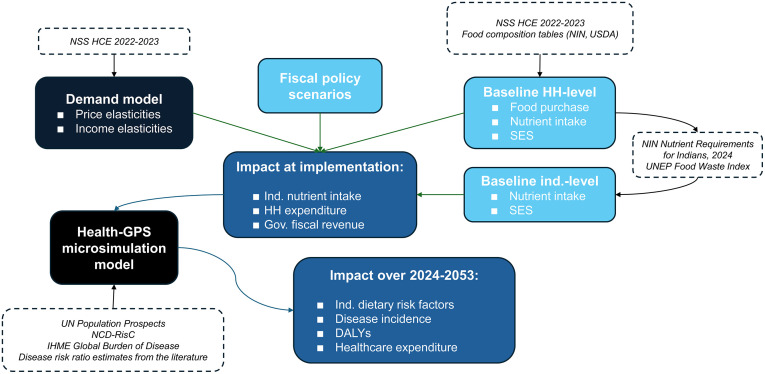
Analysis steps flow chart. DALYs, Disability-adjusted life years; Gov, Government; HH, Household; IHME, Institute for Health Metrics and Evaluation; Ind, Individual; NIN, National Institute of Nutrition; NSS HCE, National Sample Survey Household Consumption Expenditure; SES, Socio-economics characteristics; UN, United Nations; UNEP, United Nations Environmental Programme; USDA, United States Department of Agriculture.

A simplified model diagram describing the Health-GPS model can also be found in Fig D1 in S4 Text. More details on the methods related to its use are listed in Appendix D in S4 Text. Lastly, Fig 1 offers a simplified flow chart of the data sources and methodological steps taken in this analysis.

## Results

### Energy and nutrient intakes

**Table 1** presents the estimated baseline average daily nutrient intake per capita. Tables A3–A5 in S1 Text show the results for low-, middle-, and high-income groups, respectively. The average daily energy intake is lower than previous NSS-based estimates, which did not account for waste [35]. Cereals represent approximately half of the total calorie intake. Total daily energy intake increases with income. While our estimated average daily sodium intake is slightly more conservative than subnational dietary recall estimates [36], we find that 80.7% of adult males, 73.0% of adult females, and 80.8% of adolescents have daily sodium intakes above WHO-recommended level of 2,000 mg/day. Three in 10 adults also consume more saturated fat daily than WHO-recommended levels (no more than 10% of total daily energy intake). For total sugars, 10.0% of adult males and 6.5% of adult females have a daily total sugar intake above the National Health Service of the United Kingdom recommended levels (no more than 90g per day) (Fig A3 in S1 Text).

**Table 1:** Estimated average daily per capita energy and nutrient intake, full sample.

	Energy (kcal)	Sugar (g)	Sat. fat (g)	Sodium (mg)	Carbs (g)	Total fat (g)	Protein (g)
Served proc. food	103.6	0.7	1.0	134.8	14.1	4.1	2.7
Cereals	900.2	3.1	0.5	6.6	194.1	2.9	24.6
Dairy	161.8	8.5	6.9	58.5	10.9	10.5	5.9
Pulses	71.2	0.3	0.1	4.5	11.7	0.4	5.2
Oils and spices	361.9	20.4	3.8	2,650.9	23.3	29.3	1.3
F&V	193.9	9.9	5.2	20.9	22.2	9.5	4.8
Animal meats, fresh	27.2	0.0	0.4	17.4	0.1	1.5	3.3
Packaged proc. foods	89.1	0.6	1.4	185.4	9.8	4.2	3.0
Sweets	91.6	8.1	1.7	40.3	14.3	3.3	1.2
SSBs	12.0	2.2	0.0	2.7	2.7	0.1	0.2
Non-SSBs	2.6	0.0	0.0	6.5	0.6	0.0	0.0
**Total**	2,015.2	53.7	21.0	3,128.6	303.7	65.7	52.2

Notes: Survey weighted. Sat., saturated; carbs, carbohydrates; kcal, kilo calorie; g, gram; mg, milligram; proc., processed; F&V, fruits and vegetables; SSBs, sugar-sweetened beverages.

### Price elasticities of demand

**Fig 2:**
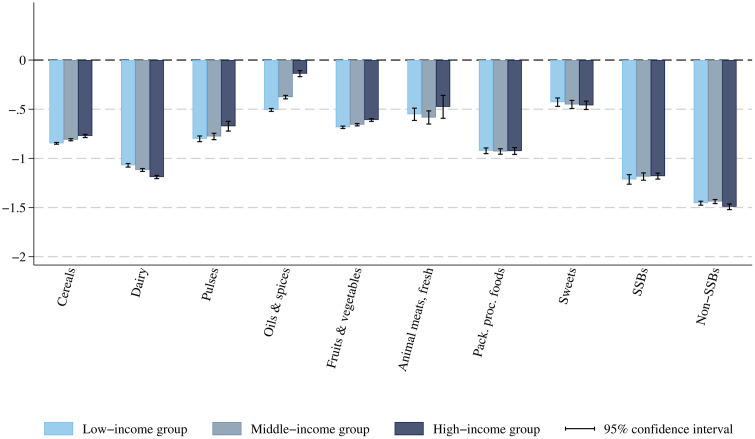
Estimated average own-price elasticity estimates, by income group. Estimated based on NSS Household Consumption Expenditure survey 2022–2023 [16] and using Deaton (1988)’s quality-adjusted Almost Ideal Demand System model [21]. Bootstrapped standard errors after 300 replications. Vertical segments represent the 95% confidence intervals. Pack., Packaged; proc., processed; SSBs, sugar-sweetened beverages.

**Table 2** shows the estimated average own- and cross-price elasticities of demand. Demand for most foods is inelastic (proportionate change in purchases smaller than proportionate change in price), except for dairy, SSBs, and non-SSBs. The demand for oils and spices is the least price-sensitive, with consumption decreasing by 3.7% for each 10% rise in oils and spices price (95% CI: 3.5%, 3.9% decrease). The demand for packaged processed foods, which mainly contain HFSS items (Table A6 in S1 Text), is close to unit-elastic (−0.92, 95% CI: −0.95, −0.89). Results indicate significant substitution and complementarity patterns between groups. F&V and fresh animal meats are complements (negative cross-price elasticities, p < 0.01). Packaged processed foods and fresh animal meats are substitutes, as well as SSBs and non-SSBs. **Fig 2** provides the estimated average own-price elasticity results by income group. Tables A7–A9 in S1 Text additionally display the estimated cross-price elasticities. In line with the international literature and previous studies in India [22,37], the demand from low-income households is more price-sensitive, except for dairy.

**Table 2:** Estimated average price elasticity estimates, full sample.

	Cereals	Dairy	Pulses	Oils and spices	F&V	Animal meats, fresh	Packaged proc. foods	Sweets	SSBs	Non-SSBs
Cereals	−0.794***	−0.089***	0.009***	−0.023***	0.051***	0.026***	−0.025***	0.025***	0.013**	0.004
	**(0.004)**	(0.007)	(0.003)	(0.005)	(0.005)	(0.008)	(0.004)	(0.005)	(0.006)	(0.004)
Dairy	−0.039***	−1.128***	−0.012***	−0.006	−0.024***	0.072***	0.048***	0.055***	0.013*	0.071***
	(0.002)	**(0.005)**	(0.002)	(0.004)	(0.003)	(0.006)	(0.004)	(0.005)	(0.008)	(0.004)
Pulses	0.015***	−0.025***	−0.744***	−0.142***	−0.169***	0.298***	−0.070***	−0.027***	−0.002	0.037***
	(0.004)	(0.008)	**(0.013)**	(0.017)	(0.009)	(0.020)	(0.009)	(0.010)	(0.008)	(0.004)
Oils and spices	−0.005**	0.019***	−0.041***	−0.372***	0.060***	0.070***	0.004	−0.031***	0.016**	0.075***
	(0.002)	(0.004)	(0.005)	**(0.008)**	(0.004)	(0.008)	(0.007)	(0.007)	(0.006)	(0.003)
F&V	0.015***	−0.018***	−0.051***	0.036***	−0.646***	−0.126***	−0.015***	0.004	0.006	0.006**
	(0.002)	(0.004)	(0.003)	(0.004)	**(0.005)**	(0.006)	(0.004)	(0.004)	(0.005)	(0.003)
Animal meats, fresh	0.008	0.131***	0.135***	0.085***	−0.234***	−0.624***	0.266***	−0.008	−0.019	0.025***
	(0.006)	(0.011)	(0.010)	(0.013)	(0.010)	**(0.033)**	(0.013)	(0.014)	(0.013)	(0.007)
Packaged proc. foods	−0.039***	0.133***	−0.062***	−0.024	−0.060***	0.416***	−0.917***	0.005	0.034***	−0.011**
	(0.004)	(0.012)	(0.007)	(0.017)	(0.011)	(0.021)	**(0.011)**	(0.009)	(0.007)	(0.005)
Sweets	0.017**	0.184***	−0.036***	−0.141***	−0.014	−0.022	0.004	−0.437***	0.031***	−0.073***
	(0.007)	(0.019)	(0.009)	(0.021)	(0.015)	(0.027)	(0.011)	**(0.018)**	(0.010)	(0.008)
SSBs	0.014	0.067	−0.020	0.050	−0.005	−0.105*	0.083***	0.061***	−1.214***	0.093***
	(0.018)	(0.062)	(0.018)	(0.045)	(0.035)	(0.056)	(0.020)	(0.022)	**(0.018)**	(0.018)
Non-SSBs	−0.000	0.196***	0.020***	0.152***	0.017**	0.043***	−0.002	−0.045***	0.037***	−1.476***
	(0.004)	(0.011)	(0.003)	(0.007)	(0.007)	(0.010)	(0.005)	(0.006)	(0.006)	**(0.009)**

Notes: Estimated based on NSS Household Consumption Expenditure survey 2022–23 [16] and using Deaton (1988)’s quality-adjusted Almost Ideal Demand System model [21]. Own-price elasticities and their standard errors are denoted in bold. Bootstrapped standard errors after 300 replications. Pack., Packaged; proc., processed; SSBs, sugar-sweetened beverages.

We provide sensitivity analyses estimating the model using a broader beverage category, i.e., combining SSBs and non-SSBs, to reduce the number of clusters dropped due to non-purchases (Table C2 in S3 Text). We also perform a sensitivity analysis in which we relax our assumption of unitary unit values for the *numeraire* group and instead assign the non-food consumer price index by state, quarter, and sector (i.e., rural or urban) (Table C3 in S3 Text) [38]. Elasticity estimates from these alternative specifications are consistent.

### Immediate policy impacts

Depending on the NPM used, HFSS foods account for 9.9% (FSSAI) or 11.4% (WHO SEARO) of total baseline energy intakes by Indians (Table A6 in S1 Text). Most of the items in the sweets and packaged processed food groups are HFSS. The difference between the two approaches is primarily driven by the extensive list of items excluded from the proposed FOPNL by FSSAI [25]. Immediate policy impacts on nutrient intake are presented in Fig A4 in S1 Text. Figs B1, B4, and B7 in S2 Text show the results for the sensitivity scenarios.

All scenarios would lead to a minor increase in expenditure on food and beverages for all income groups, from 0.6% (95% CI: 0.1%,1.1%) for low-income households for scenario 1%–1.3% (95% CI: 0.1%, 2.5%) for high-income households for scenario 2. Under scenario 2, an average household would incur a 1% increase in expenditure (95% CI: +0.0%, +1.9%) (Fig A5 in S1 Text). These results are mainly driven by a significant decrease in HFSS food purchases—particularly, packaged processed foods (high absolute price elasticity of −0.92)—and substitutions from HFSS foods to dairy and fresh animal meats. Results are similar for sensitivity scenarios (passthrough, reformulation, and subsidy) (Figs B2, B5, and B8 in S2 Text).

All scenarios would significantly increase tax revenue from foods and beverages, from +51.3% for scenario 1 (95% CI: 49.8%, 52.8%) to +110.0% for scenario 4 (95% CI: 105.3%, 114.7%) (Fig A6 in S1 Text). Results are similar for sensitivity passthrough and reformulation scenarios (Figs B3 and B6 in S2 Text). Tax revenue is predictably lower for the sensitivity scenario with GST 0% on F&V and pulses (Fig B9 in S2 Text). Behaviourally, these results are driven by a quantity (substitution) effect, with consumers shifting away from HFSS items toward dairy and fresh animal meats, and a tax increase effect, as most HFSS items belong to the packaged processed food and sweets groups, whose demand is price inelastic. The latter implies that the percentage fall in quantity demanded is smaller than the tax-induced percentage increase in price, thus increasing tax revenue. Regarding the magnitude of these results, most HFSS items are taxed at 12% or 18% at baseline; therefore, our scenarios imply a 10- to 28-percentage-point increase, which represents a significant 43% to 122% relative rise in rates for these items. The tax base is also concentrated in these tax brackets: HFSS items account for 85.3%, 93.1%, and 100% of baseline energy intake taxed at 12%, 18%, and 28%, respectively, but only 1.6% and 1.1% at the 0% and 5% rates, respectively. Most non-HFSS items are zero-rated or taxed at 5%. (Fig A2 in S1 Text).

### Long-term policy impacts

**Fig 3:**
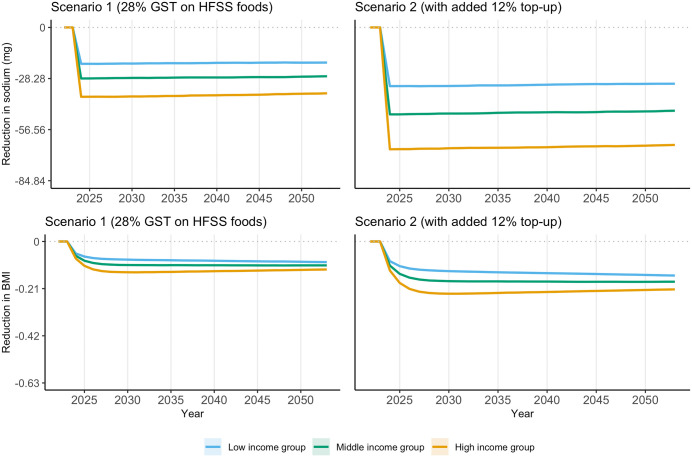
Reduction in sodium and body mass index compared to no policy change, by income group, scenarios 1 and 2. Upper bound estimates (lower bound estimates can be found in Fig A7 in S1 Text). Ninety-five% confidence interval reported as shaded area. In the top two plots on sodium, a unit of 28.28 in y-axis corresponds to 1% of adjusted baseline sodium intake (2,828 mg after adjustment according to height and weight distributions). In the bottom two plots on BMI, a unit of 0.21 in y-axis corresponds to 1% of baseline average BMI (21.43 kg/m^2^). Policy is introduced in 2024. Scenario 1: GST rate is increased to 28% for foods and beverages high in fat, sugar, and sodium (HFSS) based on the definition by the Food Safety and Standards Authority of India [25]; Scenario 2: adding a 12% top-up to the tax rate applied on HFSS foods and beverages in Scenario 1. BMI, body mass index; GST, Goods and Services Tax; HFSS, High in fat, sugar, and sodium.

**Fig 3** presents the upper bounds of reduction in sodium intake and BMI under scenarios 1 and 2 compared to no policy change over time, by income group (lower bound results available in Fig A7 in S1 Text). The reduction in daily sodium intake is persistent and stable over the 30 years, while the decrease in BMI tends to be stable after 4 years of policy implementation. Scenario 2 has greater impact on sodium intake and BMI than scenario 1. Under scenario 2, the population average reduction in daily sodium intake is approximately 45.8 mg (95% CI: −45.9, −45.7) (−1.62% of baseline sodium intake; 95% CI: −1.623%, −1.615%), ranging from 31.8 mg (95% CI: −32.1, −31.6) in the low-income group (−1.24%; 95% CI: −1.25%, −1.23%) to 66.3 mg (95% CI: −66.7, −66.0) in the high-income group (−2.07%; 95% CI: −2.08%, −2.06%). After 30 years of policy implementation, the average reduction in BMI of the entire population is 0.1705 kg/m^2^ (95% CI: −0.1709, −0.1700) (−0.796% of baseline BMI; 95% CI: −0.798%, −0.793%) under scenario 2, ranging from 0.137 kg/m^2^ (95% CI: −0.138, −0.136) in the low-income group (−0.663%; 95% CI: −0.668%, −0.659%) to 0.219 kg/m^2^ (95% CI: −0.220, −0.218) in the high-income group (−0.972%; 95% CI: −0.978%, −0.967%). Sodium and BMI reductions are consistently larger for the high-income group compared to the lower-income group. Results under scenarios 3 and 4 are reported in Figs A8 and A9 in S1 Text.

A reduction in sodium intake and a decrease in BMI reduce the risk of five major diseases: IHD, CKD, stroke, diabetes, and asthma. Over 30 years, the cumulative reduction in the incidence of the five diseases in the entire population is 5.44 million (95% CI: −5.71, −5.17) cases and 9.23 million (95% CI: −9.54, −8.91) cases (equivalent to 0.18 million (95% CI: −0.17, −0.19) and 0.31 million (95% CI: −0.32, −0.30) cases per year on average) under scenarios 1 and 2, respectively (Table A10 in S1 Text, corresponding lower bound estimates in Table A11 in S1 Text). This corresponds to an average yearly reduction of 1.01% (95% CI: −1.06%, −0.96%) and 1.72% (95% CI: −1.78%, −1.66%) in the baseline incidence number of the five diseases in India (2019). As reported in **Table 3**, the high-income group has the largest cumulative reduction in incidence rate of the five diseases, with 915 cases per 100,000 population (95% CI: −972, −858) over 30 years under scenario 2. Among diseases, the largest reductions are observed in IHD and diabetes, which made up 7.8% and 2.5% of DALYs in India in 2019, respectively [30]. Corresponding lower bound estimates and results under scenarios 3 and 4 are reported in Tables A12–A14 in S1 Text. In addition, a lower prevalence of hypertension is observed under all scenarios. In the 30th year after policy implementation, the number of hypertension cases in India decrease by 7.07 million (95% CI: −7.66, −6.48) and 11.87 million (95% CI: −12.46, −11.28) under scenarios 1 and 2, respectively (Fig A10 in S1 Text, −3.10% (95% CI: −3.36%, −2.84%) and −5.20% (95% CI: −5.46%, −4.94%) compared to the national prevalence number in India from National Family Health Survey, 2019−21; lower bound estimates in Fig A11 in S1 Text).

**Table 3:** Cumulative reduction in disease incidence rate (cases per 100,000 population) compared to no policy change, by income group, scenarios 1 and 2.

Disease	Scenario 1			Scenario 2		
	Low income (95% CI)	Middle income (95% CI)	High income (95% CI)	Low income (95% CI)	Middle income (95% CI)	High income (95% CI)
IHD	−78 (−84, −72)	−119 (−128, −110)	−180 (−190, −169)	−111 (−120, −101)	−204 (−215, −193)	−317 (−330, −304)
CKD	−16 (−23, −9)	−22 (−28, −16)	−32 (−44, −21)	−20 (−26, −14)	−41 (−46, −36)	−64 (−71, −57)
Stroke	−4 (−12, 4)	−17 (−25, −9)	−25 (−35, −14)	−14 (−22, −6)	−22 (−32, −13)	−34 (−47, −22)
Diabetes	−77 (−88, −66)	−117 (−128, −106)	−173 (−184, −162)	−129 (−138, −120)	−197 (−204, −190)	−296 (−308, −284)
Asthma	−65 (−76, −53)	−80 (−88, −72)	−104 (−118, −90)	−103 (−115, −92)	−140 (−149, −130)	−204 (−215, −192)
**Total**	**−239 (−283, −196)**	**−355 (−397, −313)**	**−513 (−571, −456)**	**−377 (−421, −334)**	**−603 (−646, −561)**	**−915 (−972, −858)**

Notes: Upper bound estimates (lower bound estimates can be found in Table A12 in S1 Text). Results estimated over a 30-year period (2024–2053). Ninety-five% confidence interval reported in parenthesis. Scenario 1: GST rate is increased to 28% for foods and beverages high in fat, sugar, and sodium based on the definition by the Food Safety and Standards Authority of India [25]; Scenario 2: adding a 12% top-up to the tax rate applied on HFSS foods and beverages in Scenario 1. IHD, Ischaemic heart disease; CKD, Chronic kidney disease; GST, Goods and Services Tax; HFSS, High in fat, sugar, and sodium.

**Fig 4:**
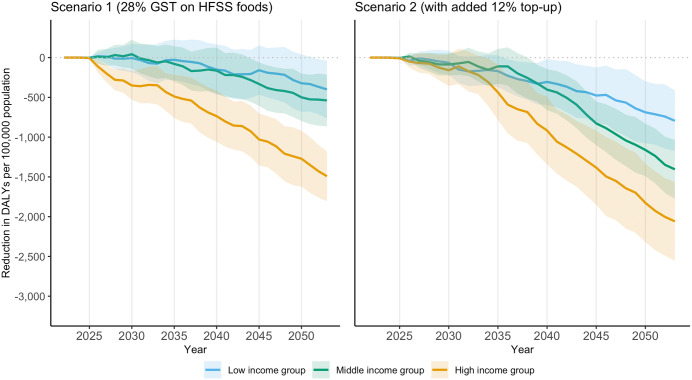
Cumulative reduction in DALYs per 100,000 population compared to no policy change, by income group, scenarios 1 and 2. Notes: Cumulative reduction in DALYs per 100,000 population of five key diseases including ischaemic heart disease, chronic kidney disease, stroke, diabetes and asthma over 2022–2053. Upper bound estimates (lower bound estimates can be found in Fig A12 in S1 Text). Ninety-five% confidence interval reported as shaded area. Policy is introduced in 2024. Scenario 1: GST rate is increased to 28% for foods and beverages high in fat, sugar, and sodium based on the definition by the Food Safety and Standards Authority of India [25]; Scenario 2: adding a 12% top-up to the tax rate applied on HFSS foods and beverages in Scenario 1. DALYs, Disability-Adjusted Life Years; GST, Goods and Services Tax; HFSS, High in fat, sugar, and sodium.

As a result, associated DALYs of IHD, CKD, stroke, diabetes, and asthma are reduced. Over 30 years, in the entire population, the cumulative reduction in DALYs is 10.36 million years (95% CI: −12.47, −8.25) and 18.98 million years (95% CI: −21.37, −16.58) under scenarios 1 and 2, respectively (Table A10 in S1 Text, equivalent to 0.35 million years (95% CI: −0.42, −0.27) and 0.63 million years (95% CI: −0.71, −0.55) per year on average). The high-income group has the largest cumulative reduction in DALYs rate, up to 2,059 DALYs per 100,000 population (95% CI: −2,553, −1,564) under scenario 2 (**Fig 4**). Meanwhile, the policy scenarios are associated with a decrease in total health expenditure of US$10.7 billion (95% CI: −11.3, −10.1) and US$18.0 billion (95% CI: −18.7, −17.3) over 30 years under scenarios 1 and 2, respectively (**Table 4**, equivalent to US$357 million (95% CI: −376, −337) and US$601 million (95% CI: −624, −578) per year on average). Lower bound estimates and results under scenarios 3 and 4 are reported in Tables A15-A17 in S1 Text, as well as in Figs A12-A14 in S1 Text.

**Table 4:** Summary of main results, scenarios 1 and 2.

		Immediate effect at implementation		Cumulative results for the five key diseases over 2024–2053		
		Change in HH expenditure on foods and beverages (%)	Change in tax revenue from foods and beverages (%)	Change in incidence rate per 100,000 population	Change in DALYs per 100,000 population	Change in total health expenditure (USD billion)
Scenario 1	Low income	+0.6% (+0.1%, +1.1%)	+51.3% (+49.8%, +52.8%)	−239 (−283, −196)	−400 (−763, −36)	−10.7 (−11.3, −10.1)
	Middle income	+0.6% (+0.1%, +1.1%)		−355 (−397, −313)	−538 (−863, −213)	
	High income	+0.8% (+0.2%, +1.4%)		−513 (−571, −456)	−1,492 (−1806, −1,177)	
Scenario 2	Low income	+0.9% (+0.0%, +1.8%)	+92.0% (+88.2%, +95.7%)	−377 (−421, −334)	−795 (−1,174, −416)	−18.0 (−18.7, −17.3)
	Middle income	+1.0% (+0.0%, +1.9%)		−603 (−646, −561)	−1,405 (−1,775, −1,035)	
	High income	+1.3% (+0.1%, + 2.5%)		−915 (−972, −858)	−2059 (−2,553, −1,564)	

Notes: Upper bound estimates (lower bound estimates can be found in Table A15 in S1 Text). Results estimated over a 30-year period (2024–2053). Ninety-five% confidence interval reported in parenthesis. Scenario 1: GST rate is increased to 28% for foods and beverages high in fat, sugar, and sodium based on the definition by the Food Safety and Standards Authority of India [25]; Scenario 2: adding a 12% top-up to the tax rate applied on HFSS foods and beverages in Scenario 1. DALYs, Disability-adjusted life years; GST, Goods and Services Tax; HFSS, High in fat, sugar, and sodium. HH, household. USD: US dollar 2024.

## Discussion

Applying a GST rate of 40% to HFSS items, based on the draft FSSAI HFSS definition, could reduce annual average disease incidence by up to 1.72% (95% CI: −1.78%, −1.66%). Over 30 years, it could prevent as many as 18.98 million (95% CI: −21.37, −16.58) DALYs and avert up to US$18.0 billion (95% CI: −18.7, −17.3) in total health expenditure on IHD, CKD, stroke, diabetes and asthma (upper bound estimates). This represents an average reduction of approximately 0.6% of total health expenditure, equivalent to US$0.44 per capita and 0.02% of GDP in India per year. A 40% GST rate on HFSS items is also associated with an immediate increase in tax revenue from foods and beverages of 92.0% (95% CI: 88.2%, 95.7%) and a minor increase in total household expenditure on foods and beverages for all income groups (ranging from +0.9% (95% CI: 0.0%, 1.8%) for low-income to +1.3% for high-income (95% CI: 0.1%, 2.5%)).

The largest absolute health gains accrue to higher-income individuals because their baseline intake of HFSS calories and sodium is greater (Tables A3, A4, and A5 in S1 Text). Nevertheless, health benefits remain significant for low-income individuals, who face higher odds of incidence and intensity of catastrophic out-of-pocket health expenditure [39]. The modelled reductions in morbidity and service use lead to a decline in total health expenditure and thus out-of-pocket expenditure - representing over 40% of total health spending in the country [34]-, which could offset the rise in food and beverage expenditure, particularly for the poorest, eliminating or mitigating the net financial burden.

Slightly higher health and economic benefits are obtained under a scenario using WHO SEARO NPM to define HFSS foods (scenarios 3 and 4, Appendix A in S1 Text), as fewer items are exempted (Tables A2 and A6 in S1 Text). Under both the draft FSSAI and WHO SEARO NPM HFSS food definition, significantly stronger health and economic benefits are associated with a 40% GST rate (scenarios 2 and 4, i.e., including an additional 12% tax on HFSS food) than with a 28% rate. The difference between lower- and upper-bound estimates is small, suggesting the results are robust to a plausible income trend (Appendix A in S1 Text). Our results are robust to potential strategic industry tax avoidance, namely tax under-shifting to prices and reformulation (Appendix B in S2 Text).

This study is based on the latest available nationally representative household consumption survey data, with information for more than 250,000 households. By linking this data with nutritional information, it provides the most up-to-date energy and nutrient intake estimates across the Indian population. Additionally, it estimates the income and price elasticity of demand for 10 food groups and three income strata, updating previous estimates based on earlier waves of this microdata from more than a decade ago. Up-to-date intake levels and elasticities are foundational inputs for projecting diet and nutrition outcomes and for simulating policy or market shocks (e.g., taxes, subsidies, income growth, inflation). This analysis utilises a novel microsimulation model, Health-GPS, to project health impacts. It simulates a large synthetic population of over 7 million individuals, representing 0.5% of the Indian population. The individual-level simulation allows us to capture heterogeneity in risk factor exposures and disease status. Health-GPS accounts for comorbidity, reflecting real-world disease patterns, and encompasses a wide range of diseases. Mortality from other causes is captured through residual mortality in Health-GPS.

Our analysis is not exempt from limitations. Our approach is a first-order attempt to individualise energy and nutrient intake from household-level data based on members’ age and sex and dietary guidelines. However, intra-household allocation of food and nutrients in India may be influenced by age and gender biases [40]. As our demand parameters are estimated at the household level, our analysis also assumes uniform relative consumption responses to the fiscal scenarios across household members, regardless of age or sex. Inherent to demand estimations based on cross-sectional data and rather aggregated food groups [9,37], we do not account for cross-price effects within such groups. Further disaggregation comes with a trade-off. It reduces the number of non-zero consumption observations per group, which undermines the reliability and identification of demand elasticities. Future research using product-level consumer panel or scanner data in India could overcome this issue [11]. Consistent with standard practice, we assume elasticity estimates are constant over time [13,14]. However, responsiveness can drift with income growth, urbanisation, demographics, and the food environment, such that holding elasticities fixed may bias long-run impacts [41].

Our microsimulation assumes no underlying time trend of diseases, which may be inconsistent with the observed increasing risks of non-communicable diseases associated with, for example, air pollution and climate change [42]. As a result, our absolute health and cost impacts estimates should be interpreted as conservative, because any unmodelled upward drift in baseline risk would scale up the number of cases and DALYs averted by a given proportional risk reduction. The analysis accounts for two pathways from diet to health, or two dietary risks: high sodium intake and high BMI (determined by energy imbalance). It does not consider the positive health impact of improving the source of energy (e.g., from F&V or pulses rather than HFSS foods) or increasing whole grain, F&V or legume intake [43]. Because these composition effects are not captured, our health impact estimates are likely conservative, particularly for policies that promote these foods (e.g., subsidy scenario zero-rating F&V and pulses) (Fig B7 in S2 Text). A fuller treatment would require multi-risk modelling with joint intake distributions and covariance to avoid double-counting, which is not currently feasible with available data.

For ease of computability, we only account for the five diseases most attributable to excess sodium intake and high BMI in terms of DALYs in India. Our health benefit estimates therefore represent an underestimate of the potential effect. The same epidemiological data on disease incidence, prevalence, mortality, and remission are applied across income groups due to the dearth of granular data. Thus, differences in estimated absolute health benefits across income groups need to be interpreted with caution, as non-communicable diseases are not necessarily diseases of affluence in India [44]. In addition, the exposure-response functions (relative risks) used in this study are derived from global meta-analyses (e.g., Institute for Health Metrics and Evaluation Global Burden of Disease), whose underlying cohorts are disproportionately from high-income countries and typically assume generalizability across different settings. Restricted by data availability, total health expenditure is extrapolated from household expenditure on health, assuming an equal distribution of health expenditure between households and the government across diseases, following the methods adopted by WHO [45]. We did not fully propagate parameter and structural uncertainty through the model. Uncertainty was addressed by bootstrapping standard errors in our demand estimation, providing confidence intervals from 20 repeated simulation runs to capture stochastic variation, and reporting sensitivity scenario analyses. This is due to data limitations regarding the variance of risk ratio estimates and the underlying distribution of dietary risk factors. As such, our uncertainty intervals are likely conservative.

The 2024 Economic Survey of India and WHO take cognisance of the increasing consumption of HFSS foods in India and outline the need to promote healthy diets [46,47]. The introduction of the 28% GST and 12% Cess on caffeinated and sweetened aerated beverages (including SSBs) in 2017, which represent only a fraction of HFSS food intake, has shown limited impacts in reducing consumption [48]. However, the 2016–2017 Kerala state-wise indirect tax of 14.5% on burgers, pizzas, tacos, doughnuts, sandwiches, pasta and bread fillings sold in restaurants (repealed following the launch of the nationwide GST by the federal government to replace all state-level indirect taxes) was associated with reduced sales [49].

Adjusting taxes within the existing GST rate structure helps avoid multiple taxation schemes. This is particularly relevant given the latest policy debates around simplifying the Indian GST rate structure to reduce financial and administrative burdens and increase revenue collection efficiency. The most recent government proposal, enacted in September 2025, includes reducing the number of rates from five to three, removing the 12% and 28% GST rates. The 12% compensatory Cess is discontinued and a 40% GST rate on “sin” goods is introduced (equivalent to the previous 28% GST + 12% Cess), still covering only caffeinated and sweetened aerated beverages and some tobacco products [50]. Curbing HFSS food consumption may require a further expansion of the “sin” goods rate to all HFSS foods.

The tax administration challenges and companies’ compliance costs associated with taxing foods based on their nutritional content should be assessed, particularly in a setting with a significant informal market. While there is no robust independent evidence for negative macroeconomic impacts of diet-related fiscal policies [51], concerns may arise regarding the economic impact of the measure on the food industry and inflation. These potential challenges should be further studied.

Increasing GST rates on HFSS foods based on nutrient content may lead to significant dietary improvements and positive health effects, reducing health expenditure and increasing government tax revenue, with only a minor impact on household spending. The latter is important since it is estimated that at least two-thirds of the rural poor could not afford a recommended healthy diet in 2011 [52]. Our nutrient intake estimates based on the latest available microdata, robust demand elasticity estimates, and simulations of various GST scenarios contribute to informing future GST reforms to improve nutrition among the Indian population.

As an increasing number of countries contemplate taxing HFSS foods [6], the results of this study may also contribute to the broader global discussion on the use of this policy. Indeed, many countries share a similar differentiated value-added or sales tax system applied to foods and beverages, as seen in India, with an opportunity to align existing rates with nutritional objectives without adding new tiers. This approach has, for example, been supported by the European Parliament in its Farm to Fork strategy [53].

India faces a rapid shift in diets towards HFSS foods. Tackling unhealthy diets is urgent. Other countries have highlighted the feasibility of regulating the marketing of unhealthy foods and beverages, the introduction of FOPNL, and shifts in relative prices to encourage significant changes towards healthier food purchases. Clear quantitative nutrient thresholds defining HFSS foods are crucial for the consistent development and implementation of these policies.

India introduced a higher 28% GST rate and a 12% Cess on caffeinated and sweetened aerated beverages, including SSBs, in July 2017 (equivalent to the “sin” goods 40% GST rate which will be applied from September 2025). Broader alignment of GST rates with nutrition and health objectives, targeting HFSS foods with higher rates, may represent the next critical step. This study has shown that it could result in meaningful reductions in the intake of nutrients of concern and significant long-run health benefits, yielding both lower health expenditure and higher tax revenue from foods and beverages while having a limited impact on household expenditure.

## Supporting information

S1 Text Appendix A. Additional tables and figures.
(PDF)

S2 Text Appendix B. Sensitivity scenarios.
(PDF)

S3 Text Appendix C. Almost Ideal Demand System (AIDS) demand model.
(PDF)

S4 Text Appendix D. Health-GPS microsimulation model.
(PDF)

S5 Text Appendix E. Income trend assumption.
(PDF)

## Abbreviations

AIDS: Almost Ideal Demand System
BMI: body mass index
CKD: chronic kidney disease
DALYs: disability-adjusted life years
F&V: fruits and vegetables
FOPNL: front-of-pack nutrition labelling
FSSAI: Food Safety and Standards Authority of India
GST: Goods and Services Tax
HFSS: high in fat, sugar and sodium
IHD: ischaemic heart disease
NIN: National Institute of Nutrition
NPM: nutrient profile model
NSSO: National Sample Survey Office
SEARO: South East Asian Region
SSBs: sugar-sweetened beverages
USDA: United States Department of Agriculture
YLLs: years of life lost
