# Supplementary material for: Taxation of foods high in fat, sugar, and sodium in India: A modelling study of health and economic impacts
Source: PLoS Med. 2026 Jan 5;23(1):e1004572. doi: 10.1371/journal.pmed.1004572 (PMC12768244; doi:10.1371/journal.pmed.1004572)
Supplement: S1 Text — Additional tables and figures. (PDF) [file pmed.1004572.s001.pdf]

S1 Text

Appendix A. Additional tables and figures

Appendix A forms part of the final submission.

Supplement to: *Roche M, Zhu J, Olney J, Laydon DJ, Joe W, Sharma M, Steele L, Sassi F. Taxation of foods high in saturated fat, sugar, and sodium in India: A modelling study of health and economic impacts. Submitted after final revisions on 12 December 2025.*

Table of Contents

Table A1. Household characteristics by income group ... 3
Table A2. Draft Food Safety and Standards Authority of India definition of HFSS foods and WHO South East Asia Region nutrient profile model category-specific thresholds ... 4
Figure A1. Baseline distribution of average daily per capita energy intake by GST rate, by food group, full sample ... 5
Figure A2. Baseline distribution of total energy intake by GST rate, by HFSS status, full sample ... 6
Table A3. Estimated average daily per capita energy and nutrient intake, low-income group ... 7
Table A4. Estimated average daily per capita energy and nutrient intake, middle-income group ... 7
Table A5. Estimated average daily per capita energy and nutrient intake, high-income group ... 8
Figure A3. Estimated daily distribution of sodium, sugar, and saturated fat intake per capita compared to daily recommendations, for adult men, adult women, and adolescents (10-18 years old) ... 8
Table A6. The proportion of total energy intake from the off-trade sector defined as high in fat, sugar, and sodium, full sample ... 9
Table A7. Estimated average price elasticities, low-income group ... 10
Table A8. Estimated average price elasticities, middle-income group ... 11
Table A9. Estimated average price elasticities, high-income group ... 12
Figure A4. Immediate impact of fiscal policy scenarios on average daily energy and nutrient intake, by income group ... 13
Figure A5. Immediate impact of fiscal policy scenarios on average total household expenditure on food and beverages, by income group ... 14
Figure A6. Impact of fiscal policy scenarios on government tax revenue from foods and beverages ... 15
Figure A7. Lower bound of reduction in sodium and body mass index compared to no policy change, by income group, scenarios 1 and 2 ... 16
Figure A8. Upper bound of reduction in sodium and body mass index compared to no policy change, by income group, scenarios 3 and 4 ... 17
Figure A9. Lower bound of reduction in sodium and body mass index compared to no policy change, by income group, scenarios 3 and 4 ... 18
Table A10. Upper bound cumulative reduction in the incidence of five key diseases in the entire population and associated change in total health expenditure and DALYs over 30 years of policy implementation, under four policy scenarios ... 19
Table A11. Lower bound cumulative reduction in the incidence of five key diseases in the entire population and associated change in total health expenditure and DALYs over 30 years of policy implementation, under four policy scenarios ... 20

Table A12. Lower bound of cumulative reduction in disease incidence rate (cases per 100,000 population) compared to no policy change, by income group, scenarios 1 and 2 21
Table A13. Upper bound of cumulative reduction in disease incidence rate (cases per 100,000 population) compared to no policy change, by income group, scenarios 3 and 4 21
Table A14. Lower bound of cumulative reduction in disease incidence rate (cases per 100,000 population) compared to no policy change, by income group, scenarios 3 and 4 22
Figure A10. Upper bound reduction in the prevalence number of hypertension in the entire population in 10, 20 and 30 years after policy implementation under four policy scenarios 23
Figure A11. Lower bound reduction in the prevalence number of hypertension in the entire population in 10, 20 and 30 years after policy implementation under four policy scenarios 24
Figure A12. Lower bound of cumulative reduction in DALYs per 100,000 population compared to no policy change, by income group, scenarios 1 and 2 25
Figure A13. Upper bound of cumulative reduction in DALYs per 100,000 population compared to baseline, scenarios 3 and 4 26
Figure A14. Lower bound of cumulative reduction in DALYs per 100,000 population compared to baseline, scenarios 3 and 4 27
Table A15. Summary of lower bound results, scenarios 1 and 2 28
Table A16. Summary of upper bound results, scenarios 3 and 4 29
Table A17. Summary of lower bound results, scenarios 3 and 4 30
References Appendix A 31

Table A1. Household characteristics by income group

	Low income	Middle income	High income	Full sample
Sector (urban=0)	0.88	0.71	0.41	0.69
Size	5.16	4.21	3.23	4.29
Head sex (female=0)	0.88	0.86	0.84	0.86
Head primary education completed	0.33	0.48	0.72	0.49
HH religion (hindu=1; others=0)	0.83	0.82	0.83	0.83
MPCE (INR)	2,592.32	4,409.12	9,515.38	5,211.49

Notes: Based on NSS Household Consumption Expenditure 2022-23 [1]. Primary education completed with at least 5 years of education. Income groups are defined by terciles of monthly per capita expenditure. Survey weighted. INR: Indian rupee. NSS: National Sample Survey.

Table A2. Draft Food Safety and Standards Authority of India definition of HFSS foods and WHO South East Asia Region nutrient profile model category-specific thresholds

Food Safety and Standards Authority of India, Labelling & Display Amendment Draft Regulations 2022

"High fat, sugar, salt (HFSS) food means a processed food product which has high levels of saturated fat or total sugar or sodium. The declared values of these ingredients are such that the product; does not satisfy the value of energy (kcal) from total sugar less than 10 percent of total energy, or from saturated fat 10 percent of total energy, and sodium less than 1 mg/1 kcal."

In this analysis, we assume processed products to be items not included in Schedule IV Category III Solid Foods/Liquid Foods exempted from FOPNL under INR in the Labelling & Display Amendment Draft Regulations 2022 (44272/2022/REGULATION-FSSAI) [2].

WHO South East Asia Region nutrient profile model

In this analysis, we define items as HFSS if nutrient content exceeds the values per 100g (or 100ml) listed below.

Food category	Food category name	Total fat (g)	Saturated fat (g)	Total sugars (g)	Sodium (g)	Energy (kcal)
1	Confectionery	8·0	NA	6·0	NA	230
2	Fine bakery wares	8·0	NA	6·0	0·25	230
3	Bread & ordinary bakery wares	8·0	NA	6·0	0·25	NA
4	Cereals	12·0	NA	9·0	0·35	NA
5a	Ready-to-eat savouries: potato, cereal or starch-based	8·0	NA	NA	0·25	230
5b	Ready-to-eat savouries: processed nuts	NA	NA	NA	0·05	NA
5c	Ready-to-eat savouries: fish-based	NA	NA	6·0	0·25	230
6a	Beverages: juices	NA	NA	6·0	NA	NA
6b	Beverages: milk & dairy-based	7·0	NA	NA	NA	NA
6c	Beverages: water-based flavoured drink	NA	NA	2·0	0·3	NA
6d	Beverages: coffee, tea, herbal infusions	NA	NA	2·0	NA	NA
6e	Beverages: cereal, grain, & tree nut-based	NA	NA	6·0	0·2	NA
7	Frozen dairy-based desserts & edible ices	8·0	NA	12·0	0·1	230
8	Curded dairy-based desserts	7·0	NA	6·0	0·1	230
9	Cheese & analogues	20·0	NA	NA	0·6	NA
10	Composite foods (prepared foods)	8·0	3·5	9·0	0·35	NA
11	Fats, oils, & fat emulsions	NA	35·0	NA	0·1	NA
12	Pasta, noodles, & like products	3·0	NA	NA	0·25	NA
13	Fresh & frozen meat, poultry, game, fish & seafood products	15·0	NA	NA	NA	NA
14a	Processed meat, poultry, & game products	8·0	NA	NA	0·4	NA
14b	Processed fish & sea food products	8·0	3·0	NA	0·4	NA
15	Fresh & frozen fruits, vegetables, & legumes	NA	NA	NA	NA	NA
16	Processed fruits & vegetables	NA	NA	NA	0·4	NA
17	Solid-form soybean products	12·0	NA	5·0	0·1	NA
18	Sauces, dips, & dressings	12·0	NA	10·0	0·3	NA

Source: Food Safety and Standards Authority of India, Labelling & Display Amendment Draft Regulations 2022 (44272/2022/REGULATION-FSSAI) and WHO South East Asia Region nutrient profile model.

Notes: We omit the 'Added sugars (g)' column for the WHO South East Asia Region nutrient profile model as we lacked information on added sugars [3]. FOPNL: front-of-pack nutritional labelling; HFSS: high in fat, sugar, and sodium; INR: Indian Nutrition Rating; NA: not applicable; WHO: World Health Organization.

Figure A1. Baseline distribution of average daily per capita energy intake by GST rate, by food group, full sample

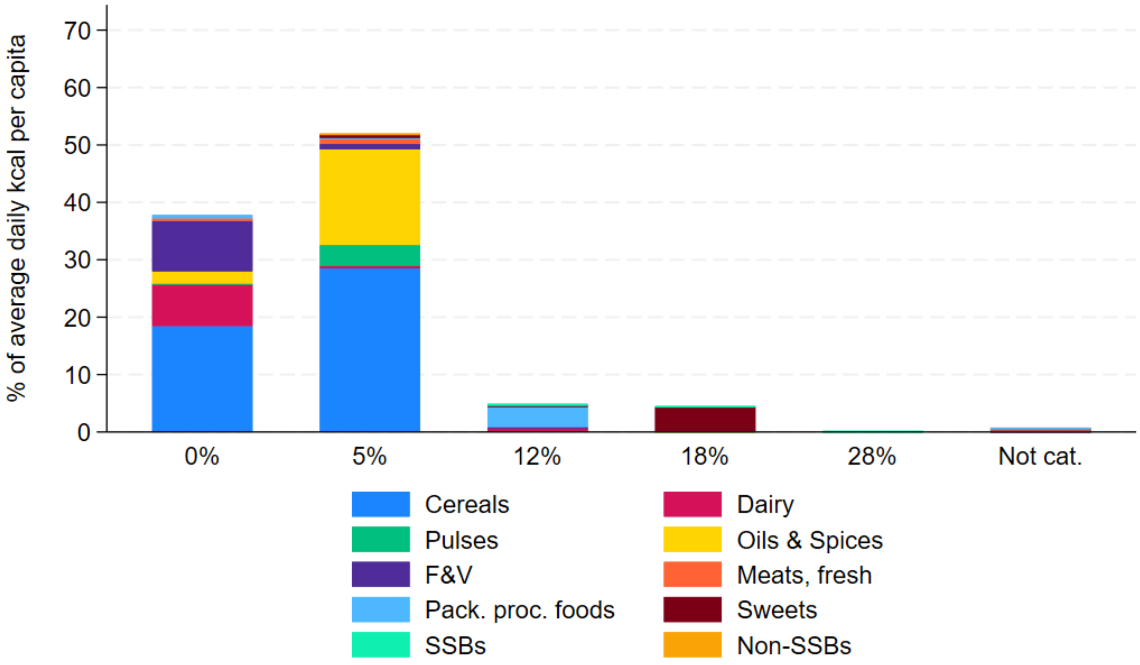

Notes: Based on NSS Household Consumption Expenditure 2022-23 [1]. On-trade food items are excluded from this analysis, i.e., NSS category ‘served processed food’. All non-PDS cereals, fresh meat/fish, arhar, khesari, curd, and honey are assumed pre-packaged (GST rates: not pre-packaged 0% / pre-packaged 5%). Tea and coffee cups assumed GST rate 5%, i.e., service GST in non-air-conditioned restaurants/bars. Survey-weighted. Pack.: packaged; proc.: processed; F&V: fruits & vegetables; NSS: National Sample Survey; SSBs: sugar-sweetened beverages. GST: Goods and Services Tax; PDS: Public Distribution System. Not. cat.: NSS items defined as ‘other’ in their respective category, thus their GST rate is assumed as the quantity-weighted average GST rate among items in the category and not categorized in this figure.

Figure A2. Baseline distribution of total energy intake by GST rate, by HFSS status, full sample

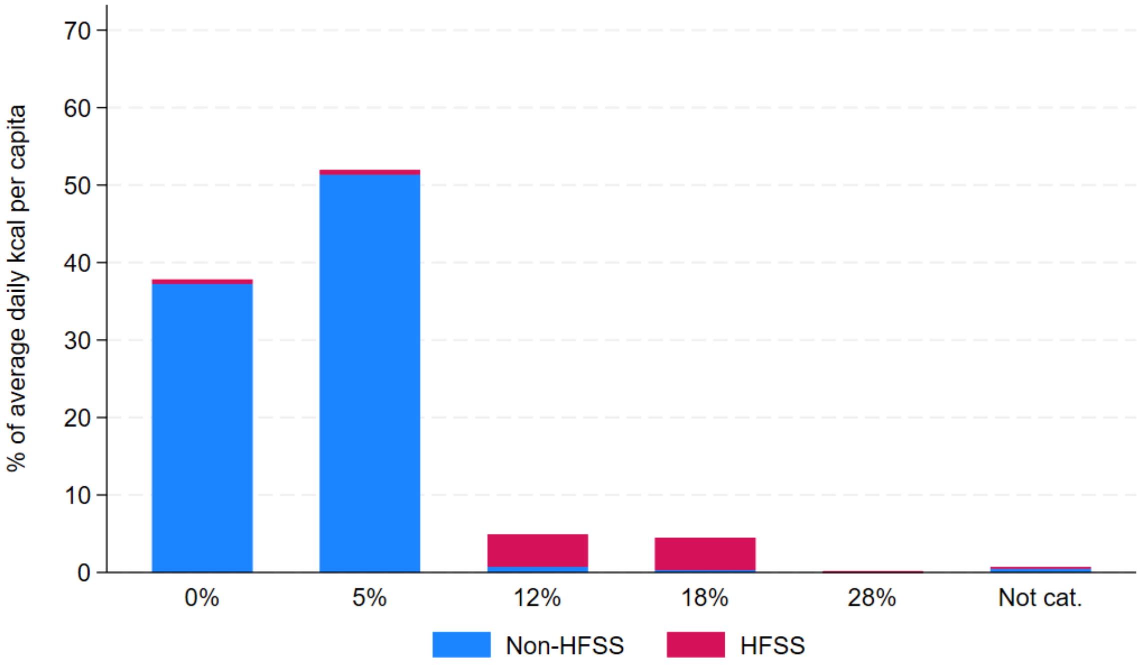

Notes: Based on NSS Household Consumption Expenditure 2022-23 [1]. HFSS definition based on the FSSAI Labelling & Display Amendment Draft Regulations, 2022 (44272/2022/REGULATION-FSSAI) [2]. On-trade food items are excluded from this analysis, i.e., NSS category 'served processed food'. All non-PDS cereals, fresh meat/fish, arhar, khesari, curd, and honey are assumed pre-packaged (GST rates: not pre-packaged 0% / pre-packaged 5%). Tea and coffee cups assumed GST rate 5%, i.e., service GST in non-air-conditioned restaurants/bars. Survey-weighted. FSSAI: Food Safety and Standards Authority of India. GST: Goods and Services Tax; HFSS: High in fat, salt, and sugar; NSS: National Sample Survey; PDS: Public Distribution System. Not. cat.: NSS items defined as 'other' in their respective category, thus their GST rate is assumed as the quantity-weighted average GST rate among items in the category and not categorized in this figure.

Table A3. Estimated average daily per capita energy and nutrient intake, low-income group

	Energy (kcal)	Sugar (g)	Sat. fat (g)	Sodium (mg)	Carbs (g)	Total fat (g)	Protein (g)
Served proc. food	79.2	0.4	0.7	102.0	11.7	2.8	1.8
Cereals	922.2	3.1	0.5	6.6	199.0	2.9	25.1
Dairy	102.0	5.5	4.3	35.1	7.1	6.5	3.8
Pulses	58.9	0.3	0.1	3.7	9.7	0.4	4.2
Oils & spices	304.0	17.2	2.7	2,462.0	19.6	24.6	1.0
F&V	119.4	6.4	2.0	17.4	17.3	4.0	3.5
Animal meats, fresh	19.5	0.0	0.3	12.5	0.0	1.1	2.4
Packaged proc. foods	55.7	0.3	0.9	111.9	5.9	2.8	1.8
Sweets	61.4	5.4	1.1	26.6	9.7	2.2	0.8
SSBs	3.1	0.6	0.0	0.8	0.7	0.0	0.0
Non-SSBs	1.8	0.0	0.0	4.3	0.4	0.0	0.0
**Total**	**1,727.3**	**39.3**	**12.4**	**2,782.9**	**281.1**	**47.2**	**44.5**

Notes: Based on NSS Household Consumption Expenditure 2022-23 [1]. Survey weighted. Income groups are defined by terciles of monthly per capita expenditure. Sat.: saturated; carbs: carbohydrates; kcal: kilo calorie; g: gram; mg: milligram. proc.: processed; F&V: fruits & vegetables; SSBs: sugar-sweetened beverages.

Table A4. Estimated average daily per capita energy and nutrient intake, middle-income group

	Energy (kcal)	Sugar (g)	Sat. fat (g)	Sodium (mg)	Carbs (g)	Total fat (g)	Protein (g)
Served proc. food	88.9	0.5	0.8	114.1	12.6	3.4	2.1
Cereals	926.2	3.2	0.5	6.8	199.5	3.0	25.3
Dairy	168.6	8.9	7.1	59.3	11.4	10.9	6.2
Pulses	72.9	0.3	0.1	4.6	11.9	0.5	5.3
Oils & spices	378.4	21.7	3.9	2,747.8	24.7	30.4	1.4
F&V	191.1	9.8	5.0	21.4	22.3	9.2	4.9
Animal meats, fresh	27.8	0.0	0.4	17.8	0.1	1.5	3.4
Packaged proc. foods	84.2	0.6	1.3	173.4	9.2	4.0	2.9
Sweets	85.2	7.7	1.5	37.1	13.5	3.0	1.1
SSBs	9.9	1.8	0.0	2.5	2.2	0.1	0.1
Non-SSBs	2.6	0.0	0.0	6.6	0.6	0.0	0.0
**Total**	**2,035.9**	**54.5**	**20.6**	**3,191.3**	**308.1**	**65.9**	**52.7**

Notes: Based on NSS Household Consumption Expenditure 2022-23 [1]. Survey weighted. Income groups are defined by terciles of monthly per capita expenditure. Sat.: saturated; carbs: carbohydrates; kcal: kilo calorie; g: gram; mg: milligram. proc.: processed; F&V: fruits & vegetables; SSBs: sugar-sweetened beverages.

Table A5. Estimated average daily per capita energy and nutrient intake, high-income group

	Energy (kcal)	Sugar (g)	Sat. fat (g)	Sodium (mg)	Carbs (g)	Total fat (g)	Protein (g)
Served proc. food	151.8	1.3	1.8	200.7	18.9	6.5	4.5
Cereals	842.2	2.8	0.4	6.3	181.6	2.7	23.0
Dairy	231.5	11.9	9.9	88.1	15.2	15.2	8.4
Pulses	85.2	0.4	0.1	5.5	13.9	0.5	6.2
Oils & spices	418.2	23.1	5.1	2,785.0	26.6	33.9	1.6
F&V	293.7	14.4	9.8	24.9	28.4	17.1	6.5
Animal meats, fresh	36.5	0.0	0.5	23.4	0.1	2.0	4.5
Packaged proc. foods	137.8	1.1	2.2	294.3	15.5	6.3	4.8
Sweets	137.9	11.9	2.6	61.8	21.2	5.1	1.8
SSBs	26.1	4.7	0.1	5.5	5.8	0.1	0.4
Non-SSBs	3.7	0.0	0.0	9.2	0.8	0.0	0.1
**Total**	**2,364.5**	**71.5**	**32.5**	**3,504.7**	**328.0**	**89.5**	**61.7**

Notes: Based on NSS Household Consumption Expenditure 2022-23 [1]. Survey weighted. Income groups are defined by terciles of monthly per capita expenditure. Sat.: saturated; carbs: carbohydrates; kcal: kilo calorie; g: gram; mg: milligram. proc.: processed; F&V: fruits & vegetables; SSBs: sugar-sweetened beverages.

Figure A3. Estimated daily distribution of sodium, sugar, and saturated fat intake per capita compared to daily recommendations, for adult men, adult women, and adolescents (10-18 years old)

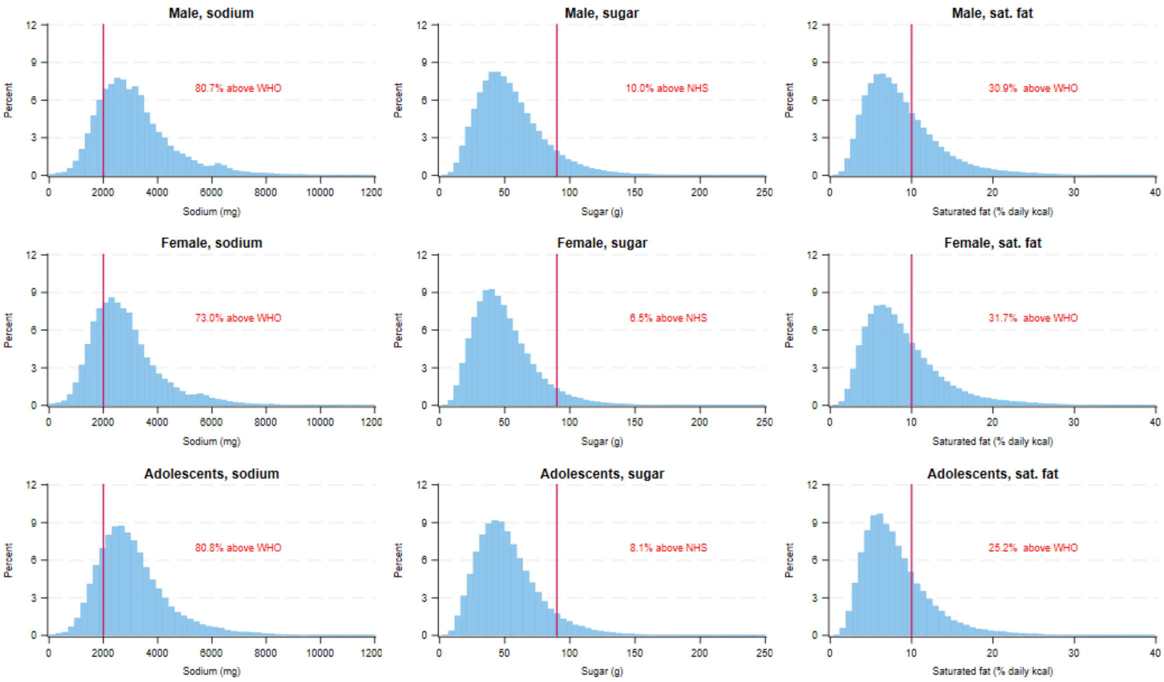

Notes: Based on NSS Household Consumption Expenditure 2022-23 [1]. NHS: National Health Service, here referring to the NHS daily recommendation for total sugar intake in the United Kingdom (90g), which used instead of the WHO daily recommendation for free sugars as we missed information on free sugars; Sat.: saturated; WHO: World Health Organization, here referring to the WHO daily recommendations for the intake of sodium and saturated fat (no more than 2,000mg for sodium and 10% of total energy intake for saturated fat); kcal: kilo calorie; g: gram; mg: milligram.

Table A6. The proportion of total energy intake from the off-trade sector defined as high in fat, sugar, and sodium, full sample

	FSSAI HFSS	WHO SEARO NPM
Cereals	0.000	0.000
Dairy	0.005	0.137
Pulses	0.000	0.000
Oils & spices	0.000	0.017
F&V	0.053	0.000
Animal meats, fresh	0.000	0.000
Packaged proc. foods	0.956	0.956
Sweets	0.911	0.998
SSBs	0.752	1.000
Non-SSBs	0.000	0.000
**Total**	**0.099**	**0.114**

Notes: On-trade food items are excluded from this analysis, i.e., NSS category 'served processed food'. Survey-weighted. FSSAI HFSS: definition of foods and beverages high in fat, sugar, and sodium by the Food Safety and Standards Authority of India [2]; WHO SEARO NPM: items with nutrient content above at least one of the respective thresholds set by the World Health Organization South East Asia Region nutrient profile model [3]. proc.: processed; F&V: fruits & vegetables; HFSS: high in fat, sodium, and sugar; NSS: National Sample Survey; SSBs: sugar-sweetened beverages.

Table A7. Estimated average price elasticities, low-income group

	Cereals	Dairy	Pulses	Oils & spices	F&V	Animal meats, fresh	Packaged proc. foods	Sweets	SSBs	Non-SSBs
Cereals	**-0.847*****	-0.052***	0.003	-0.029***	0.050***	0.032***	-0.005	0.017***	0.029***	-0.001
	**(0.004)**	(0.007)	(0.003)	(0.006)	(0.006)	(0.007)	(0.004)	(0.005)	(0.005)	(0.004)
Dairy	-0.048***	**-1.070*****	-0.034***	-0.063***	-0.049***	0.090***	0.042***	0.036***	0.034***	0.045***
	(0.004)	**(0.009)**	(0.004)	(0.006)	(0.005)	(0.008)	(0.006)	(0.007)	(0.009)	(0.005)
Pulses	0.009**	-0.054***	**-0.801*****	-0.066***	-0.118***	0.188***	-0.059***	-0.013	-0.004	0.030***
	(0.004)	(0.010)	**(0.015)**	(0.018)	(0.012)	(0.020)	(0.009)	(0.010)	(0.007)	(0.004)
Oils & spices	-0.007***	-0.003	-0.019***	**-0.507*****	0.043***	0.042***	-0.008	-0.005	0.014***	0.044***
	(0.002)	(0.005)	(0.005)	**(0.008)**	(0.005)	(0.008)	(0.005)	(0.005)	(0.004)	(0.003)
F&V	0.014***	-0.013***	-0.045***	0.018***	**-0.684*****	-0.111***	-0.017***	-0.008	0.004	0.002
	(0.002)	(0.005)	(0.004)	(0.005)	**(0.006)**	(0.007)	(0.005)	(0.005)	(0.005)	(0.003)
Animal meats, fresh	0.002	0.154***	0.086***	0.020	-0.226***	**-0.552*****	0.249***	-0.036***	-0.015	0.017**
	(0.006)	(0.013)	(0.011)	(0.016)	(0.012)	**(0.032)**	(0.013)	(0.014)	(0.013)	(0.007)
Packaged proc. foods	-0.033***	0.126***	-0.077***	-0.093***	-0.095***	0.453***	**-0.923*****	-0.015	0.025***	-0.008
	(0.006)	(0.018)	(0.009)	(0.019)	(0.015)	(0.023)	**(0.015)**	(0.011)	(0.008)	(0.006)
Sweets	0.009	0.152***	-0.031**	-0.079***	-0.057***	-0.079**	-0.014	**-0.428*****	-0.019	-0.054***
	(0.009)	(0.026)	(0.012)	(0.023)	(0.021)	(0.032)	(0.014)	**(0.022)**	(0.012)	(0.009)
SSBs	0.105***	0.344***	-0.047*	0.062	-0.048	-0.136	0.079***	-0.075**	**-1.214*****	0.120***
	(0.028)	(0.102)	(0.024)	(0.055)	(0.054)	(0.086)	(0.030)	(0.035)	**(0.025)**	(0.026)
Non-SSBs	-0.012**	0.166***	0.021***	0.119***	0.005	0.049***	0.004	-0.037***	0.040***	**-1.456*****
	(0.006)	(0.016)	(0.004)	(0.010)	(0.009)	(0.014)	(0.006)	(0.007)	(0.007)	**(0.010)**

Notes: Estimated based on NSS Household Consumption Expenditure survey 2022-23 [1] and using Deaton (1988)'s quality-adjusted Almost Ideal Demand System model [4]. Income groups are defined by terciles of monthly per capita expenditure. Own-price elasticities and their standard errors are denoted in bold. Bootstrapped standard errors after 300 replications. Pack.: Packaged; proc.: processed; F&V: fruits & vegetables; SSBs: sugar-sweetened beverages. * $p<0{\cdot}1$, ** $p<0{\cdot}05$, *** $p<0{\cdot}01$.

Table A8. Estimated average price elasticities, middle-income group

		Cereals	Dairy	Pulses	Oils & spices	F&V	Animal meats, fresh	Packaged proc. foods	Sweets	SSBs	Non-SSBs
Cereals		**-0.810*****	-0.075***	0.006*	-0.033***	0.049***	0.024***	-0.015***	0.021***	0.039***	-0.003
		**(0.006)**	(0.009)	(0.003)	(0.007)	(0.006)	(0.009)	(0.005)	(0.006)	(0.007)	(0.005)
Dairy		-0.033***	**-1.118*****	-0.003	-0.012***	-0.027***	0.070***	0.044***	0.050***	0.009	0.068***
		(0.003)	**(0.008)**	(0.003)	(0.004)	(0.004)	(0.007)	(0.005)	(0.005)	(0.007)	(0.005)
Pulses		0.012***	0.014	**-0.777*****	-0.116***	-0.169***	0.298***	-0.073***	-0.042***	0.011	0.037***
		(0.004)	(0.011)	**(0.017)**	(0.018)	(0.013)	(0.023)	(0.012)	(0.013)	(0.009)	(0.005)
Oils & spices		-0.009***	0.016***	-0.034***	**-0.377*****	0.054***	0.080***	-0.009	-0.023***	0.019***	0.065***
		(0.003)	(0.005)	(0.005)	**(0.009)**	(0.006)	(0.011)	(0.007)	(0.007)	(0.006)	(0.004)
F&V		0.013***	-0.020***	-0.050***	0.027***	**-0.657*****	-0.117***	-0.015***	-0.001	0.004	0.003
		(0.002)	(0.004)	(0.003)	(0.005)	**(0.006)**	(0.007)	(0.005)	(0.005)	(0.005)	(0.003)
Animal meats, fresh		0.003	0.121***	0.119***	0.081***	-0.206***	**-0.584*****	0.265***	-0.032**	-0.023*	0.033***
		(0.007)	(0.012)	(0.010)	(0.017)	(0.012)	**(0.034)**	(0.014)	(0.015)	(0.013)	(0.008)
Packaged proc. foods		-0.029***	0.117***	-0.062***	-0.063***	-0.064***	0.433***	**-0.930*****	-0.002	0.022***	-0.000
		(0.005)	(0.013)	(0.009)	(0.018)	(0.013)	(0.022)	**(0.014)**	(0.011)	(0.008)	(0.006)
Sweets		0.009	0.171***	-0.050***	-0.120***	-0.036**	-0.075**	-0.004	**-0.452*****	-0.016	-0.058***
		(0.008)	(0.020)	(0.011)	(0.022)	(0.019)	(0.031)	(0.013)	**(0.021)**	(0.012)	(0.009)
SSBs		0.082***	0.030	0.004	0.056	-0.029	-0.133**	0.052**	-0.044	**-1.185*****	0.087***
		(0.020)	(0.061)	(0.017)	(0.041)	(0.038)	(0.063)	(0.022)	(0.027)	**(0.019)**	(0.022)
Non-SSBs		-0.009*	0.199***	0.019***	0.125***	0.006	0.061***	0.009*	-0.034***	0.037***	**-1.439*****
		(0.005)	(0.014)	(0.003)	(0.009)	(0.009)	(0.012)	(0.005)	(0.006)	(0.008)	**(0.010)**

Notes: Estimated based on NSS Household Consumption Expenditure survey 2022-23 [1] and using Deaton (1988)'s quality-adjusted Almost Ideal Demand System model [4]. Income groups are defined by terciles of monthly per capita expenditure. Own-price elasticities and their standard errors are denoted in bold. Bootstrapped standard errors after 300 replications. Pack.: Packaged; proc.: processed; F&V: fruits & vegetables; SSBs: sugar-sweetened beverages. * p<0·1, ** p<0·05, *** p<0·01.

Table A9. Estimated average price elasticities, high-income group

	Cereals	Dairy	Pulses	Oils & spices	F&V	Animal meats, fresh	Packaged proc. foods	Sweets	SSBs	Non-SSBs
Cereals	**-0.772*****	-0.140***	0.013**	-0.068***	0.079***	0.024	-0.034***	0.032***	0.066***	-0.003
	**(0.008)**	(0.015)	(0.006)	(0.012)	(0.010)	(0.019)	(0.009)	(0.010)	(0.013)	(0.009)
Dairy	-0.030***	**-1.190*****	0.005	0.028***	-0.024***	0.083***	0.062***	0.075***	-0.002	0.103***
	(0.003)	**(0.008)**	(0.003)	(0.005)	(0.005)	(0.010)	(0.006)	(0.007)	(0.010)	(0.007)
Pulses	0.013**	0.035**	**-0.673*****	-0.350***	-0.246***	0.507***	-0.077***	-0.055***	0.032**	0.053***
	(0.006)	(0.017)	**(0.025)**	(0.029)	(0.023)	(0.042)	(0.021)	(0.021)	(0.016)	(0.008)
Oils & spices	-0.018***	0.058***	-0.108***	**-0.139*****	0.089***	0.170***	0.003	-0.031***	0.048***	0.108***
	(0.004)	(0.008)	(0.009)	**(0.016)**	(0.009)	(0.019)	(0.011)	(0.011)	(0.010)	(0.006)
F&V	0.014***	-0.027***	-0.060***	0.052***	**-0.609*****	-0.150***	-0.019***	0.004	-0.005	0.010**
	(0.002)	(0.006)	(0.005)	(0.006)	**(0.007)**	(0.010)	(0.007)	(0.007)	(0.007)	(0.005)
Animal meats, fresh	0.004	0.155***	0.193***	0.196***	-0.255***	**-0.476*****	0.351***	-0.032	-0.026	0.052***
	(0.008)	(0.018)	(0.016)	(0.024)	(0.017)	**(0.059)**	(0.021)	(0.023)	(0.023)	(0.014)
Packaged proc. foods	-0.023***	0.151***	-0.046***	-0.017	-0.056***	0.472***	**-0.926*****	0.017	0.023**	0.007
	(0.005)	(0.015)	(0.011)	(0.019)	(0.015)	(0.027)	**(0.017)**	(0.012)	(0.011)	(0.009)
Sweets	0.010	0.212***	-0.042***	-0.095***	-0.011	-0.062*	0.016	**-0.461*****	-0.023*	-0.060***
	(0.006)	(0.020)	(0.013)	(0.023)	(0.019)	(0.037)	(0.014)	**(0.022)**	(0.013)	(0.010)
SSBs	0.053***	-0.035	0.025	0.125***	-0.053	-0.083	0.038*	-0.041*	**-1.181*****	0.079***
	(0.013)	(0.050)	(0.016)	(0.033)	(0.033)	(0.061)	(0.022)	(0.021)	**(0.015)**	(0.023)
Non-SSBs	-0.005	0.221***	0.020***	0.138***	0.018**	0.060***	0.011	-0.034***	0.040***	**-1.493*****
	(0.004)	(0.015)	(0.004)	(0.008)	(0.009)	(0.016)	(0.007)	(0.007)	(0.010)	**(0.015)**

Notes: Estimated based on NSS Household Consumption Expenditure survey 2022-23 [1] and using Deaton (1988)'s quality-adjusted Almost Ideal Demand System model [4]. Income groups are defined by terciles of monthly per capita expenditure. Own-price elasticities and their standard errors are denoted in bold. Bootstrapped standard errors after 300 replications. Pack.: Packaged; proc.: processed; F&V: fruits & vegetables; SSBs: sugar-sweetened beverages. * p<0·1, ** p<0·05, *** p<0·01.

Figure A4. Immediate impact of fiscal policy scenarios on average daily energy and nutrient intake, by income group

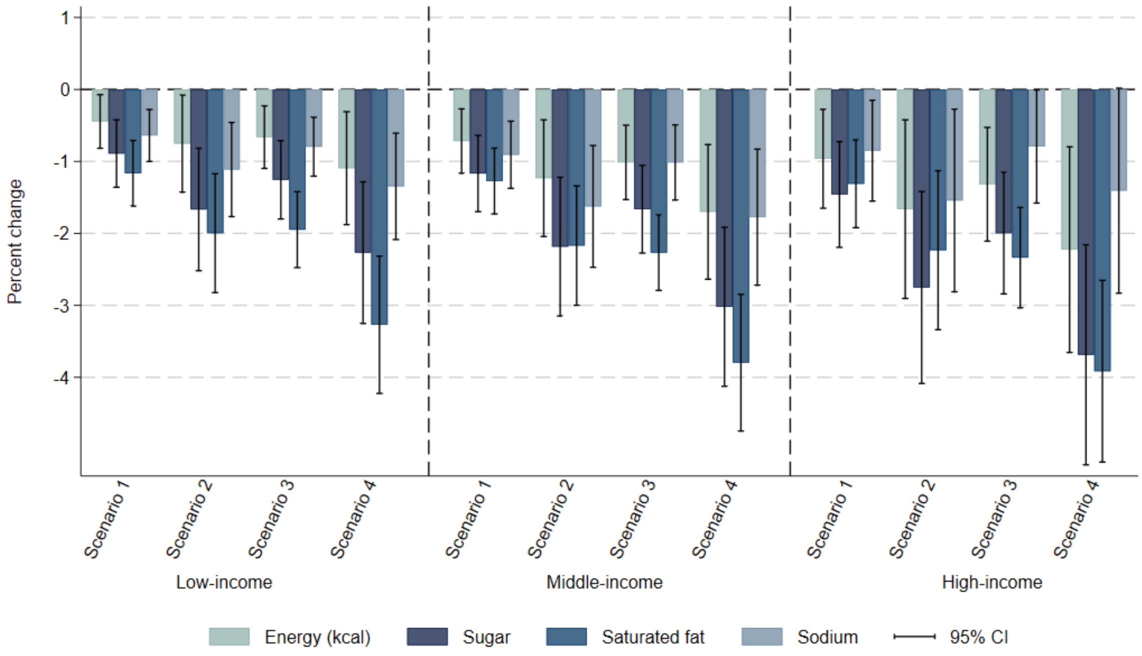

Notes: Vertical segments represent the 95% confidence intervals. Survey weighted. Scenario 1: defining items for which GST rate is increased to 28% based on the definition of foods and beverages high in fat, sugar, and sodium by the Food Safety and Standards Authority of India [2]; Scenario 2: adding a 12% top-up to the tax rate applied on HFSS foods and beverages in Scenario 1. Scenario 3: scenario defining items for which GST rate is increased to 28% if their nutrient content is above at least one of the respective thresholds set by the World Health Organization South East Asia Region nutrient profile model [3]; Scenarios 4: adding a 12% top-up to the tax rate applied on HFSS foods and beverages in Scenario 3. GST: Goods and Services Tax. HFSS: High in fat, sodium, and sugar.

Figure A5. Immediate impact of fiscal policy scenarios on average total household expenditure on food and beverages, by income group

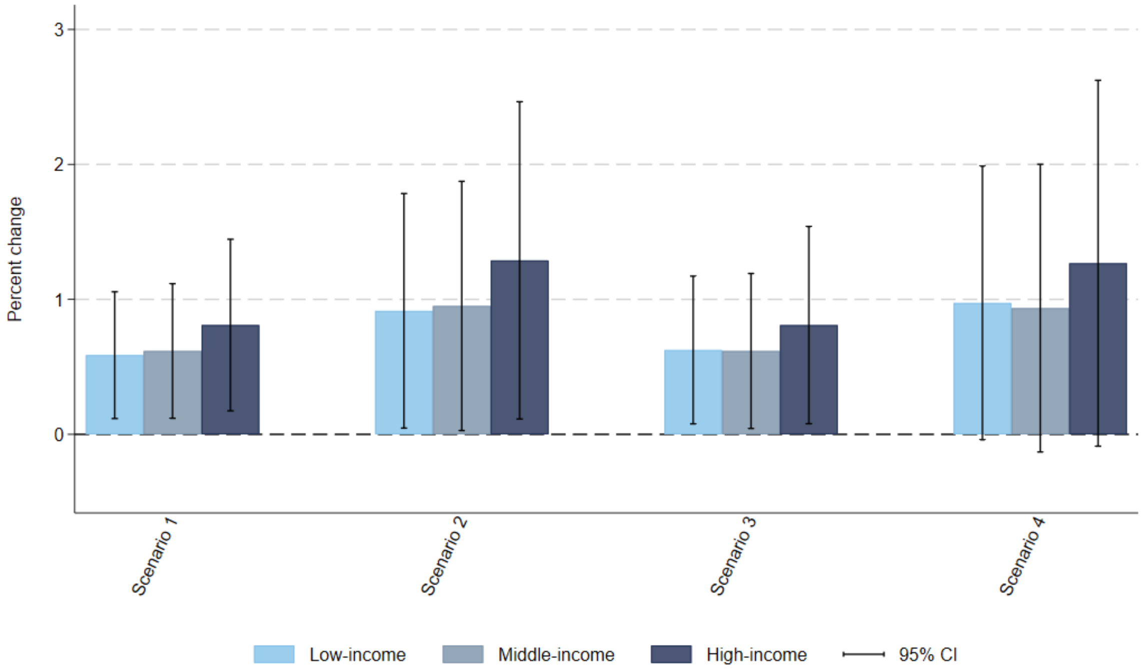

Notes: Vertical segments represent the 95% confidence intervals. Survey weighted. Scenario 1: defining items for which GST rate is increased to 28% based on the definition of foods and beverages high in fat, sugar, and sodium by the Food Safety and Standards Authority of India [2]; Scenario 2: adding a 12% top-up to the tax rate applied on HFSS foods and beverages in Scenario 1. Scenario 3: scenario defining items for which GST rate is increased to 28% if their nutrient content is above at least one of the respective thresholds set by the World Health Organization South East Asia Region nutrient profile model [3]; Scenarios 4: adding a 12% top-up to the tax rate applied on HFSS foods and beverages in Scenario 3. GST: Goods and Services Tax. HFSS: High in fat, sodium, and sugar.

Figure A6. Impact of fiscal policy scenarios on government tax revenue from foods and beverages

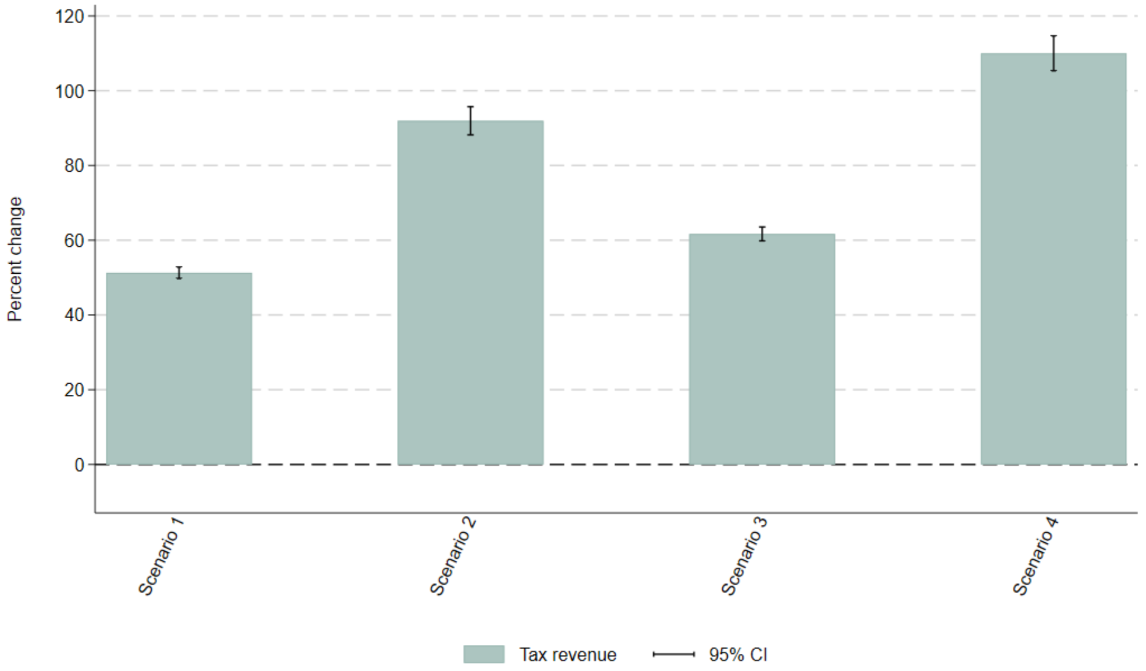

Notes: Vertical segments represent the 95% confidence intervals. Survey weighted. Scenario 1: defining items for which GST rate is increased to 28% based on the definition of foods and beverages high in fat, sugar, and sodium by the Food Safety and Standards Authority of India [2]; Scenario 2: adding a 12% top-up to the tax rate applied on HFSS foods and beverages in Scenario 1. Scenario 3: scenario defining items for which GST rate is increased to 28% if their nutrient content is above at least one of the respective thresholds set by the World Health Organization South East Asia Region nutrient profile model [3]; Scenarios 4: adding a 12% top-up to the tax rate applied on HFSS foods and beverages in Scenario 3. GST: Goods and Services Tax. HFSS: High in fat, sodium, and sugar.

Figure A7. Lower bound of reduction in sodium and body mass index compared to no policy change, by income group, scenarios 1 and 2

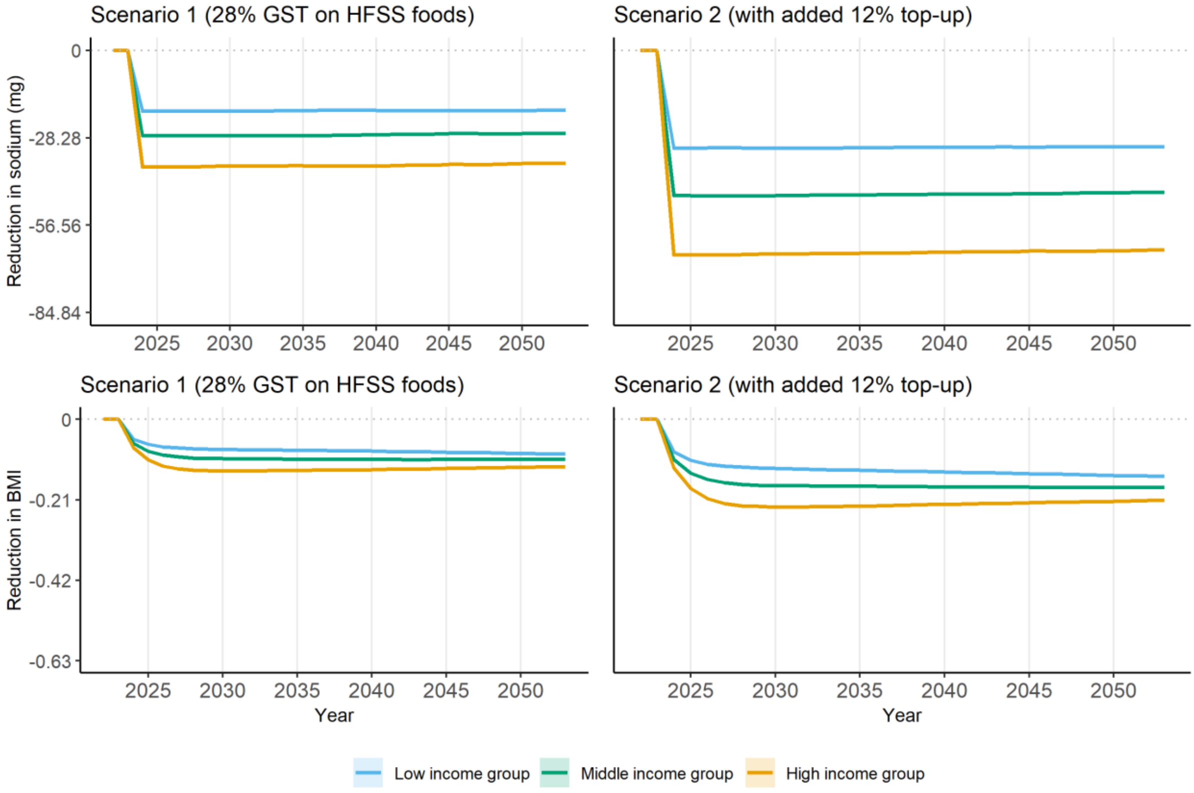

Notes: Lower bound estimates (upper bound estimates can be found in **Figure 3**). 95% confidence interval reported as shaded area. In the top two plots on sodium, a unit of 28.28 in y-axis corresponds to 1% of adjusted baseline sodium intake (2,828mg after adjustment according to height and weight distributions). In the bottom two plots on BMI, a unit of 0.21 in y-axis corresponds to 1% of baseline average BMI (21.43kg/m^2). Policy is introduced in 2024. Scenario 1: defining items for which GST rate is increased to 28% based on the definition of foods and beverages high in fat, sugar, and sodium by the Food Safety and Standards Authority of India [2]; Scenario 2: adding a 12% top-up to the tax rate applied on HFSS foods and beverages in Scenario 1. BMI: body mass index; GST: Goods and Services Tax. HFSS: High in fat, sodium, and sugar.

Figure A8. Upper bound of reduction in sodium and body mass index compared to no policy change, by income group, scenarios 3 and 4

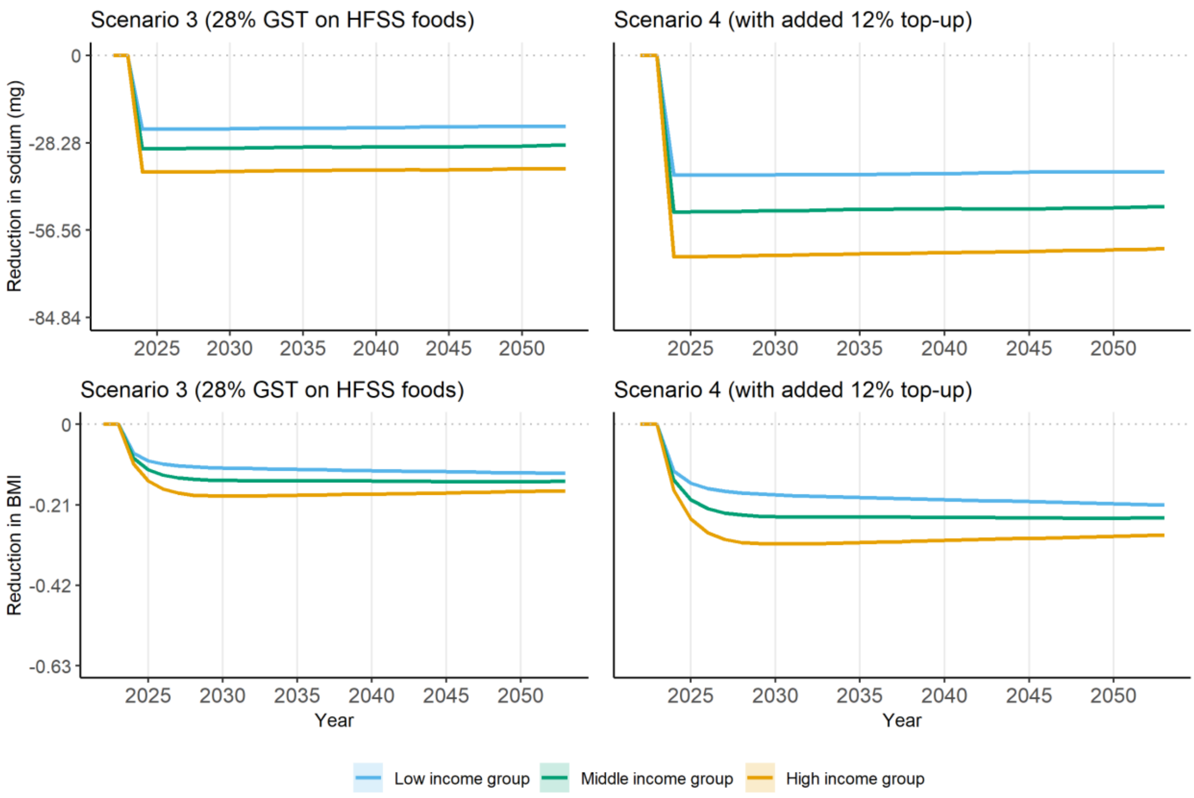

Notes: Upper bound estimates (lower bound estimates can be found in **Figure A9**). 95% confidence interval reported as shaded area. In the top two plots on sodium, a unit of 28.28 in y-axis corresponds to 1% of adjusted baseline sodium intake (2,828mg after adjustment according to height and weight distributions). In the bottom two plots on BMI, a unit of 0.21 in y-axis corresponds to 1% of baseline average BMI (21.43kg/m^2). Policy is introduced in 2024. Scenario 3: scenario defining items for which GST rate is increased to 28% if their nutrient content is above at least one of the respective thresholds set by the World Health Organization South East Asia Region nutrient profile model [3]. Scenarios 4: adding a 12% top-up to the tax rate applied on HFSS foods and beverages in Scenario 3. BMI: body mass index; GST: Goods and Services Tax. HFSS: High in fat, sodium, and sugar.

Figure A9. Lower bound of reduction in sodium and body mass index compared to no policy change, by income group, scenarios 3 and 4

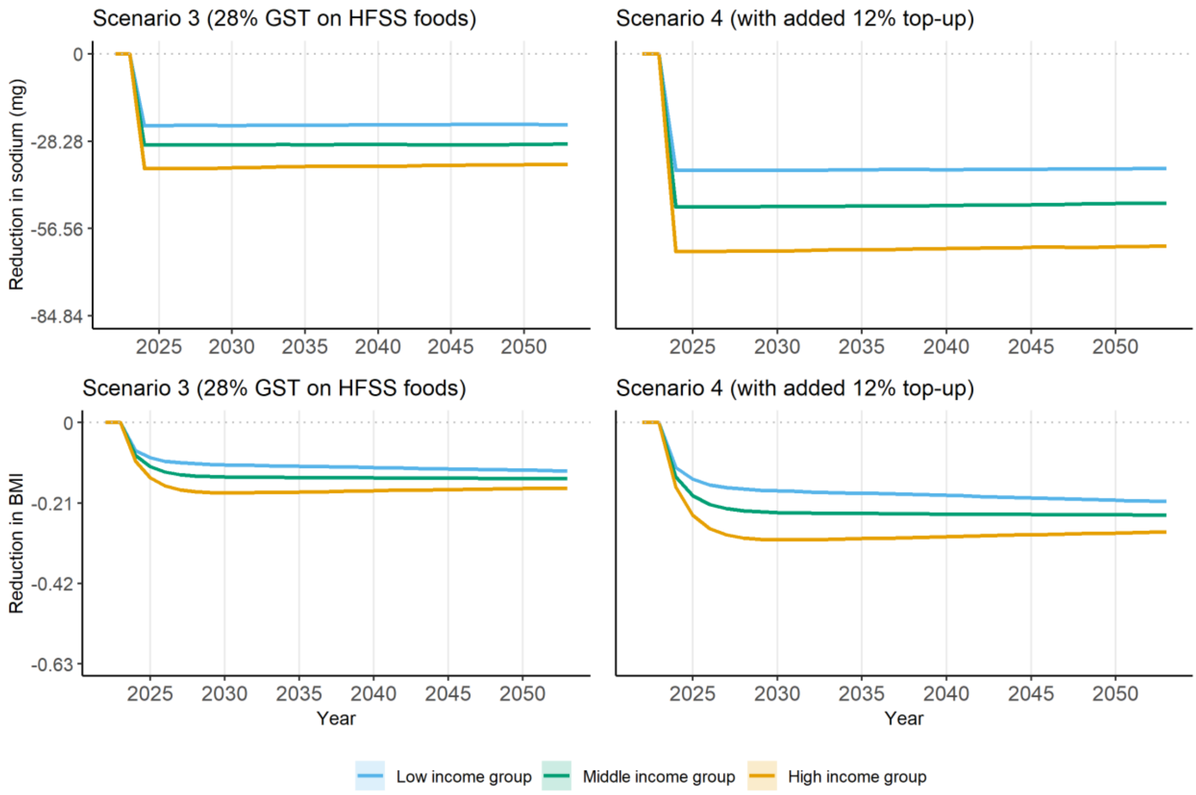

Notes: Lower bound estimates (upper bound estimates can be found in **Figure A8**). 95% confidence interval reported as shaded area. In the top two plots on sodium, a unit of 28.28 in y-axis corresponds to 1% of adjusted baseline sodium intake (2,828mg after adjustment according to height and weight distributions). In the bottom two plots on BMI, a unit of 0.21 in y-axis corresponds to 1% of baseline average BMI (21.43kg/m^2). Policy is introduced in 2024. Scenario 3: scenario defining items for which GST rate is increased to 28% if their nutrient content is above at least one of the respective thresholds set by the World Health Organization South East Asia Region nutrient profile model [3]. Scenarios 4: adding a 12% top-up to the tax rate applied on HFSS foods and beverages in Scenario 3. BMI: body mass index; GST: Goods and Services Tax. HFSS: High in fat, sodium, and sugar.

Table A10. Upper bound cumulative reduction in the incidence of five key diseases in the entire population and associated change in total health expenditure and DALYs over 30 years of policy implementation, under four policy scenarios

Disease	Annual incidence at baseline* (million cases)	Scenario 1		Scenario 2		Scenario 3		Scenario 4	
		CR[a] (million cases, %)	THE[b] (USD billion)	CR[a] (million cases, %)	THE[b] (USD billion)	CR[a] (million cases, %)	THE[b] (USD billion)	CR[a] (million cases, %)	THE[b] (USD billion)
IHD	6.45	-1.85 (-0.96)	-4.53	-3.06 (-1.58)	-7.50	-2.44 (-1.26)	-5.98	-4.00 (-2.07)	-9.83
CKD	2.10	-0.34 (-0.54)	-0.92	-0.59 (-0.93)	-1.59	-0.45 (-0.72)	-1.23	-0.77 (-1.22)	-2.07
Stroke	1.20	-0.22 (-0.61)	-0.69	-0.34 (-0.94)	-1.08	-0.25 (-0.71)	-0.81	-0.41 (-1.15)	-1.32
Diabetes	3.40	-1.80 (-1.76)	-2.79	-3.05 (-2.99)	-4.73	-2.52 (-2.47)	-3.90	-4.07 (-3.99)	-6.31
Asthma	4.77	-1.24 (-0.87)	-1.77	-2.20 (-1.53)	-3.13	-1.84 (-1.28)	-2.62	-2.95 (-2.06)	-4.21
**Total**	**17.92**	**-5.44 (-1.01)**	**-10.70**	**-9.23 (-1.72)**	**-18.03**	**-7.50 (-1.40)**	**-14.55**	**-12.20 (-2.27)**	**-23.74**
**Cumulative reduction in DALYs (million)**		**-10.36**		**-18.98**		**-15.55**		**-27.37**	

Notes: Upper bound estimates (lower bound estimates can be found in **Table A11**). Results estimated over a 30-year period (2024-2053). [a]CR: Cumulative reduction over 30 years, in million cases. Percentage of annual baseline incidence number (2019) in India per year in parenthesis, calculated as cumulative reduction over 30 years ÷ (annual incidence × 30). [b]THE: Total health expenditure over 30 years, in billion US dollar 2024. Scenario 1: defining items for which GST rate is increased to 28% based on the definition of foods and beverages high in fat, sugar, and sodium by the Food Safety and Standards Authority of India [2]; Scenario 2: adding a 12% top-up to the tax rate applied on HFSS foods and beverages in Scenario 1. Scenario 3: defining items for which GST rate is increased to 28% if their nutrient content is above at least one of the respective thresholds set by the World Health Organization South East Asia Region nutrient profile model [3]; Scenarios 4: adding a 12% top-up to the tax rate applied on HFSS foods and beverages in Scenario 3. IHD: Ischemic heart disease. CKD: Chronic kidney disease. GST: Goods and Services Tax. HFSS: High in fat, sodium, and sugar. *Source: Institute for Health Metrics and Evaluation (IHME), Global Burden of Disease Study 2019 (GBD 2019) results [5].

Table A11. Lower bound cumulative reduction in the incidence of five key diseases in the entire population and associated change in total health expenditure and DALYs over 30 years of policy implementation, under four policy scenarios

Disease	Annual incidence at baseline* (million cases)	Scenario 1		Scenario 2		Scenario 3		Scenario 4	
		CR[a] (million cases, %)	THE[b] (USD billion)	CR[a] (million cases, %)	THE[b] (USD billion)	CR[a] (million cases, %)	THE[b] (USD billion)	CR[a] (million cases, %)	THE[b] (USD billion)
IHD	6.45	-1.78 (-0.92)	-4.38	-3.06 (-1.58)	-7.50	-2.44 (-1.26)	-5.98	-3.94 (-2.03)	-9.66
CKD	2.10	-0.37 (-0.58)	-0.99	-0.63 (-1.00)	-1.71	-0.48 (-0.76)	-1.29	-0.81 (-1.28)	-2.18
Stroke	1.20	-0.21 (-0.57)	-0.66	-0.30 (-0.84)	-0.97	-0.26 (-0.71)	-0.82	-0.40 (-1.11)	-1.27
Diabetes	3.40	-1.77 (-1.74)	-2.75	-3.02 (-2.96)	-4.68	-2.50 (-2.45)	-3.87	-4.01 (-3.93)	-6.21
Asthma	4.77	-1.25 (-0.88)	-1.79	-2.05 (-1.44)	-2.93	-1.78 (-1.24)	-2.54	-2.87 (-2.01)	-4.10
**Total**	**17.92**	**-5.39 (-1.00)**	**-10.57**	**-9.06 (-1.69)**	**-17.78**	**-7.45 (-1.39)**	**-14.50**	**-12.02 (-2.24)**	**-23.42**
**Cumulative reduction in DALYs (million)**		**-12.26**		**-19.97**		**-17.68**		**-24.82**	

Notes: Lower bound estimates (upper bound estimates can be found in **Table A10**). Results estimated over a 30-year period (2024-2053). [a]CR: Cumulative reduction over 30 years, in million cases. Percentage of annual baseline incidence number (2019) in India per year in parenthesis, calculated as cumulative reduction over 30 years ÷ (annual incidence × 30). [b]THE: Total health expenditure over 30 years, in billion US dollar 2024. Scenario 1: defining items for which GST rate is increased to 28% based on the definition of foods and beverages high in fat, sugar, and sodium by the Food Safety and Standards Authority of India [2]; Scenario 2: adding a 12% top-up to the tax rate applied on HFSS foods and beverages in Scenario 1. Scenario 3: defining items for which GST rate is increased to 28% if their nutrient content is above at least one of the respective thresholds set by the World Health Organization South East Asia Region nutrient profile model [3]; Scenarios 4: adding a 12% top-up to the tax rate applied on HFSS foods and beverages in Scenario 3. IHD: Ischemic heart disease. CKD: Chronic kidney disease. GST: Goods and Services Tax. HFSS: High in fat, sodium, and sugar. *Source: Institute for Health Metrics and Evaluation (IHME), Global Burden of Disease Study 2019 (GBD 2019) results [5].

Table A12. Lower bound of cumulative reduction in disease incidence rate (cases per 100,000 population) compared to no policy change, by income group, scenarios 1 and 2

Disease	**Scenario 1**			**Scenario 2**		
	Low income	Middle income	High income	Low income	Middle income	High income
IHD	-68	-110	-191	-115	-188	-332
CKD	-13	-27	-36	-25	-45	-60
Stroke	-9	-12	-22	-14	-18	-32
Diabetes	-72	-119	-171	-119	-197	-305
Asthma	-67	-79	-105	-101	-130	-184
**Total**	**-230**	**-347**	**-525**	**-374**	**-577**	**-913**

Notes: Lower bound estimates (upper bound estimates can be found in **Table 3**). Results estimated over a 30-year period (2024-2053). Scenario 1: GST rate is increased to 28% for foods and beverages high in fat, sugar, and sodium based on the definition by the Food Safety and Standards Authority of India [2]; Scenario 2: adding a 12% top-up to the tax rate applied on HFSS foods and beverages in Scenario 1. IHD: Ischemic heart disease. CKD: Chronic kidney disease. GST: Goods and Services Tax. HFSS: High in fat, sugar, and sodium.

Table A13. Upper bound of cumulative reduction in disease incidence rate (cases per 100,000 population) compared to no policy change, by income group, scenarios 3 and 4

Disease	**Scenario 3**			**Scenario 4**		
	Low income	Middle income	High income	Low income	Middle income	High income
IHD	-96	-161	-243	-154	-267	-403
CKD	-17	-31	-49	-31	-47	-84
Stroke	-9	-18	-27	-14	-32	-41
Diabetes	-117	-158	-234	-179	-260	-389
Asthma	-86	-123	-164	-136	-194	-270
**Total**	**-325**	**-490**	**-717**	**-513**	**-800**	**-1188**

Notes: Upper bound estimates (lower bound estimates can be found in **Table A14**). Results estimated over a 30-year period (2024-2053). Scenario 3: scenario defining items for which GST rate is increased to 28% if their nutrient content is above at least one of the respective thresholds set by the World Health Organization South East Asia Region nutrient profile model [3]. Scenarios 4: adding a 12% top-up to the tax rate applied on HFSS foods and beverages in Scenario 3. IHD: Ischemic heart disease. CKD: Chronic kidney disease. GST: Goods and Services Tax. HFSS: High in fat, sugar, and sodium.

Table A14. Lower bound of cumulative reduction in disease incidence rate (cases per 100,000 population) compared to no policy change, by income group, scenarios 3 and 4

Disease	**Scenario 3**			**Scenario 4**		
	Low income	Middle income	High income	Low income	Middle income	High income
IHD	-100	-153	-248	-148	-253	-413
CKD	-24	-28	-47	-33	-54	-80
Stroke	-11	-14	-29	-19	-21	-44
Diabetes	-108	-162	-239	-167	-253	-400
Asthma	-86	-120	-151	-139	-188	-253
**Total**	**-329**	**-477**	**-714**	**-506**	**-768**	**-1190**

Notes: Lower bound estimates (upper bound estimates can be found in **Table A13**). Results estimated over a 30-year period (2024-2053). Scenario 3: scenario defining items for which GST rate is increased to 28% if their nutrient content is above at least one of the respective thresholds set by the World Health Organization South East Asia Region nutrient profile model [3]. Scenarios 4: adding a 12% top-up to the tax rate applied on HFSS foods and beverages in Scenario 3. IHD: Ischemic heart disease. CKD: Chronic kidney disease. GST: Goods and Services Tax. HFSS: High in fat, sugar, and sodium.

Figure A10. Upper bound reduction in the prevalence number of hypertension in the entire population in 10, 20 and 30 years after policy implementation under four policy scenarios

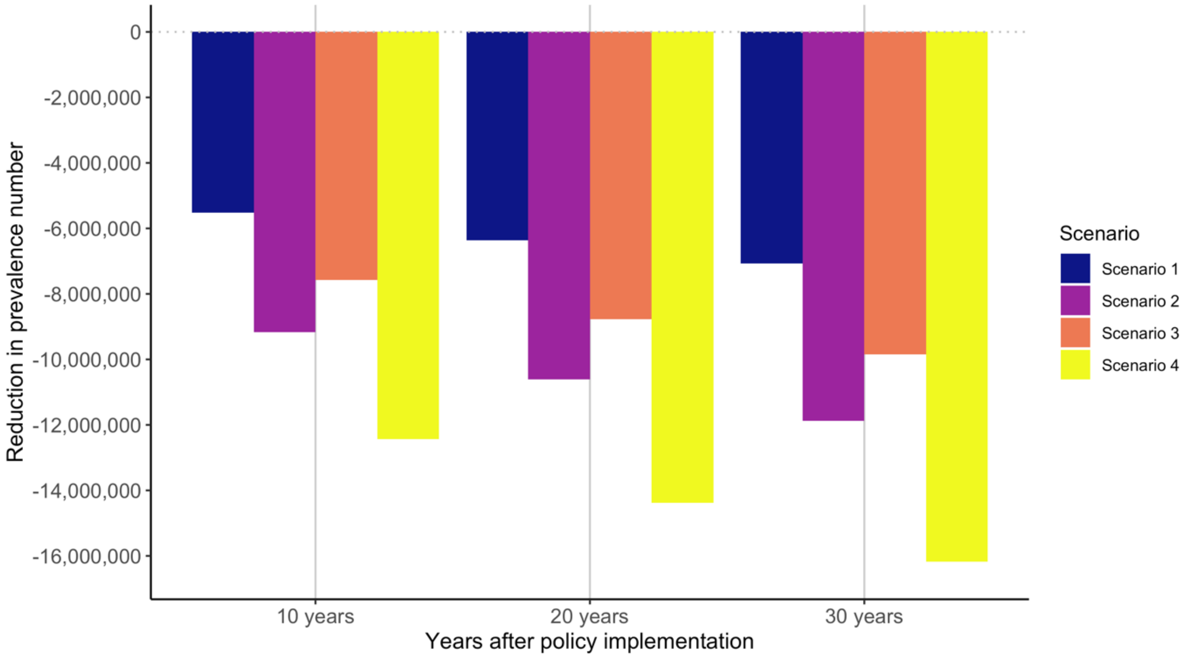

Notes: Reduction in the prevalence number of hypertension is calculated after microsimulation, with the reduction in sodium intake and BMI from Health-GPS microsimulation and relative risks of sodium and BMI on the incidence of hypertension from the literature. Upper bound estimates (lower bound estimates can be found in **Figure A10**). Scenario 1: defining items for which GST rate is increased to 28% based on the definition of foods and beverages high in fat, sugar, and sodium by the Food Safety and Standards Authority of India [2]; Scenario 2: adding a 12% top-up to the tax rate applied on HFSS foods and beverages in Scenario 1. Scenario 3: defining items for which GST rate is increased to 28% if their nutrient content is above at least one of the respective thresholds set by the World Health Organization South East Asia Region nutrient profile model [3]; Scenarios 4: adding a 12% top-up to the tax rate applied on HFSS foods and beverages in Scenario 3.GST: Goods and Services Tax. HFSS: High in fat, sodium, and sugar.

Figure A11. Lower bound reduction in the prevalence number of hypertension in the entire population in 10, 20 and 30 years after policy implementation under four policy scenarios

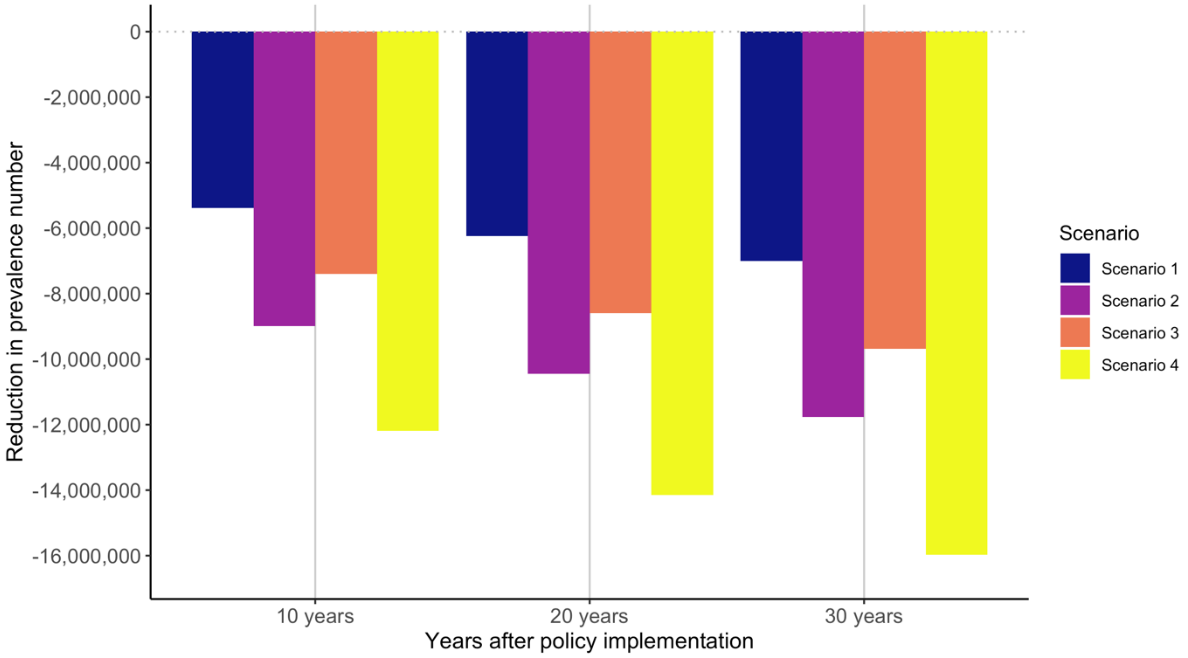

Notes: Reduction in the prevalence number of hypertension is calculated after microsimulation, with the reduction in sodium intake and BMI from Health-GPS microsimulation and relative risks of sodium and BMI on the incidence of hypertension from the literature. Lower bound estimates (upper bound estimates can be found in **Figure A9**). Scenario 1: defining items for which GST rate is increased to 28% based on the definition of foods and beverages high in fat, sugar, and sodium by the Food Safety and Standards Authority of India [2]; Scenario 2: adding a 12% top-up to the tax rate applied on HFSS foods and beverages in Scenario 1. Scenario 3: defining items for which GST rate is increased to 28% if their nutrient content is above at least one of the respective thresholds set by the World Health Organization South East Asia Region nutrient profile model [3]; Scenarios 4: adding a 12% top-up to the tax rate applied on HFSS foods and beverages in Scenario 3. GST: Goods and Services Tax. HFSS: High in fat, sodium, and sugar.

Figure A12. Lower bound of cumulative reduction in DALYs per 100,000 population compared to no policy change, by income group, scenarios 1 and 2

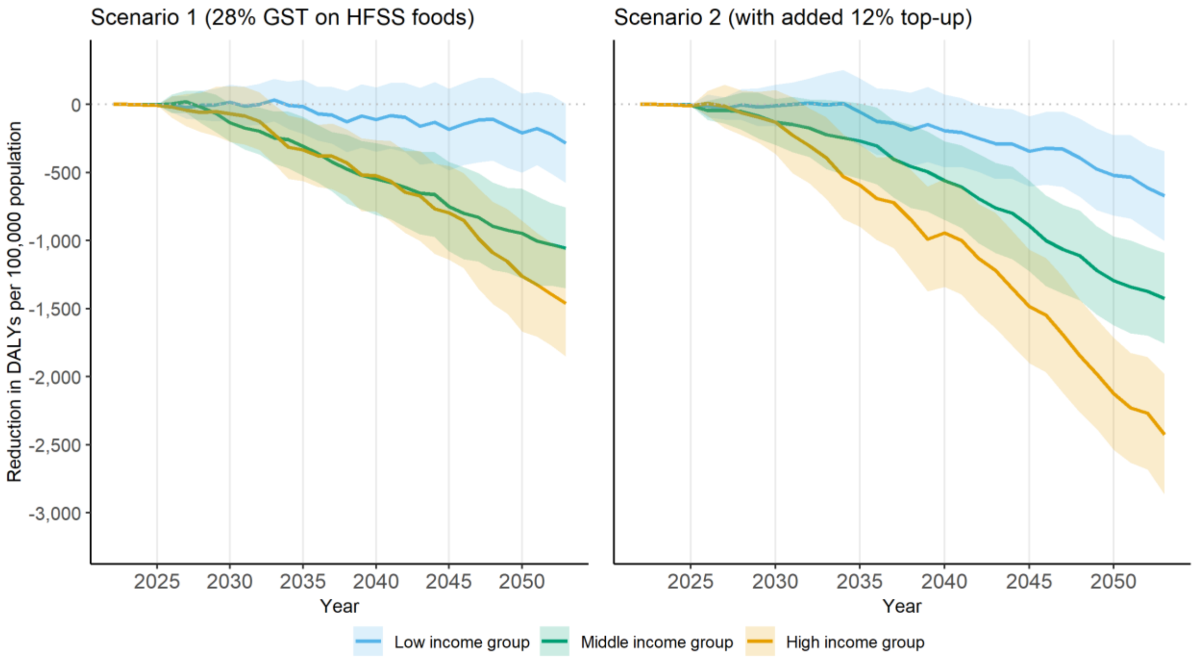

Notes: Cumulative reduction in DALYs per 100,000 population of five key diseases including ischemic heart disease, chronic kidney disease, stroke, diabetes and asthma over 2022-2053. Lower bound estimates (upper bound estimates can be found in **Figure 4**). 95% confidence interval reported as shaded area. Policy is introduced in 2024. Scenario 1: GST rate is increased to 28% for foods and beverages high in fat, sugar, and sodium based on the definition by the Food Safety and Standards Authority of India [2]; Scenario 2: adding a 12% top-up to the tax rate applied on HFSS foods and beverages in Scenario 1. DALYs: Disability-Adjusted Life Years. HFSS: High in fat, sodium, and sugar.

Figure A13. Upper bound of cumulative reduction in DALYs per 100,000 population compared to baseline, scenarios 3 and 4

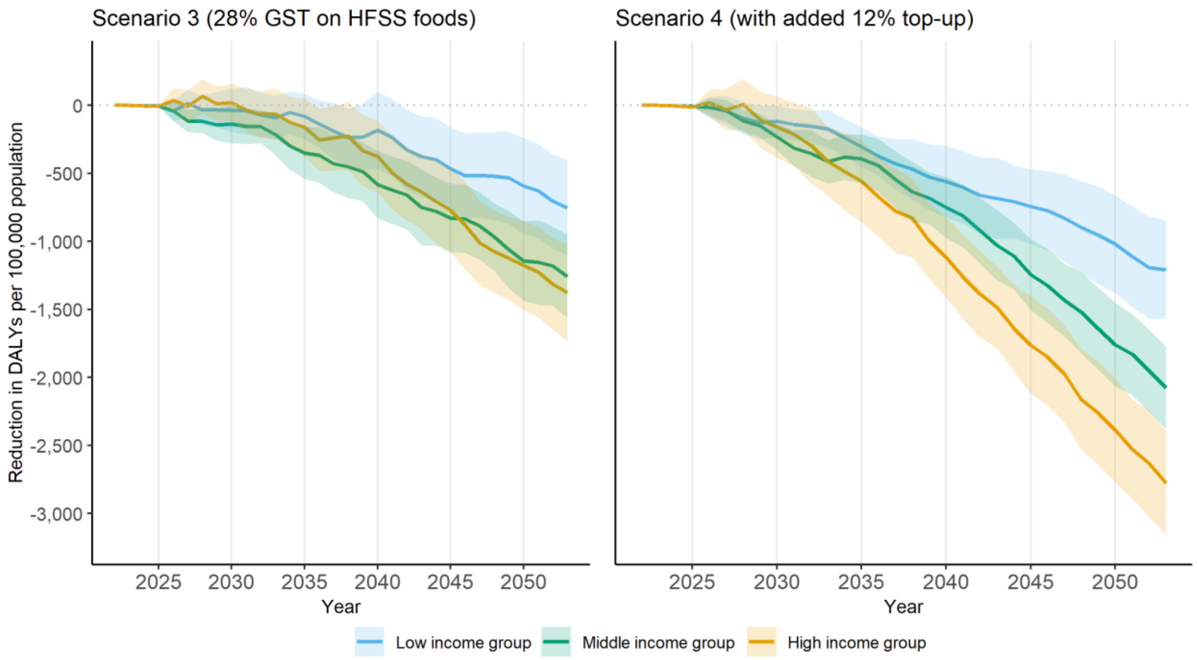

Notes: Cumulative reduction in DALYs per 100,000 population of five key diseases including ischemic heart disease, chronic kidney disease, stroke, diabetes and asthma over 2022-2053. Upper bound estimates (lower bound estimates can be found in **Figure A14**). 95% confidence interval reported as shaded area. Policy is introduced in 2024. Scenario 3: defining items for which GST rate is increased to 28% if their nutrient content is above at least one of the respective thresholds set by the World Health Organization South East Asia Region nutrient profile model [3]. Scenarios 4: adding a 12% top-up to the tax rate applied on HFSS foods and beverages in Scenario 3. DALYs: Disability-Adjusted Life Years. HFSS: High in fat, sodium, and sugar.

Figure A14. Lower bound of cumulative reduction in DALYs per 100,000 population compared to baseline, scenarios 3 and 4

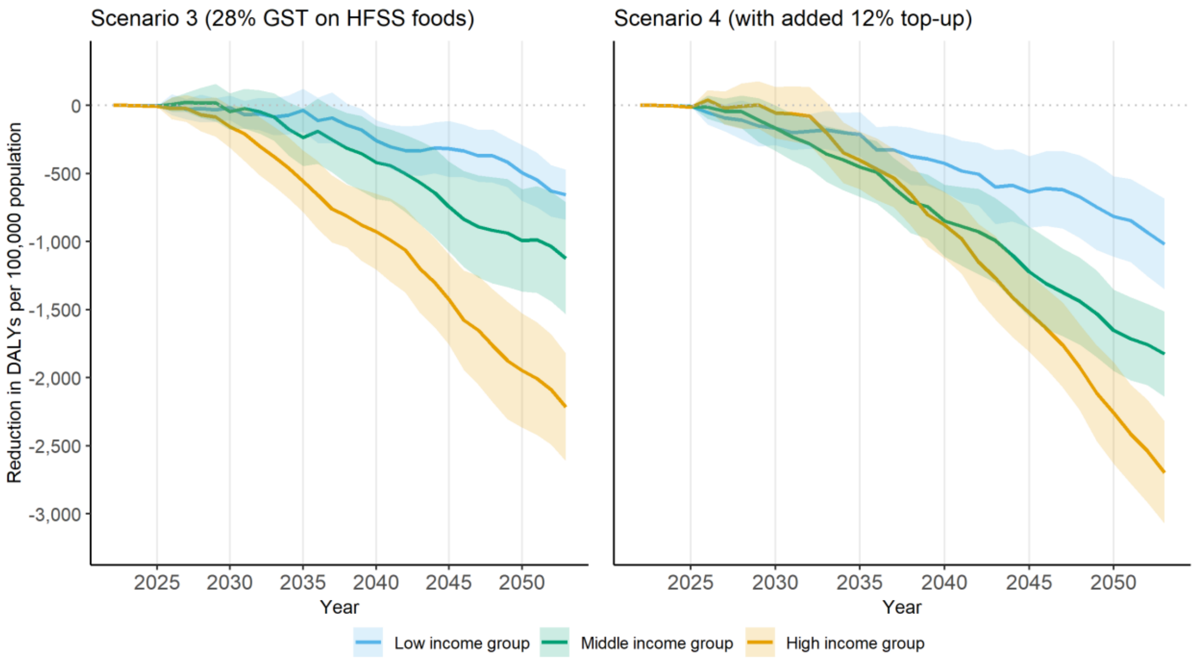

Notes: Cumulative reduction in DALYs per 100,000 population of five key diseases including ischemic heart disease, chronic kidney disease, stroke, diabetes and asthma over 2022-2053. Lower bound estimates (upper bound estimates can be found in **Figure A13**). 95% confidence interval reported as shaded area. Policy is introduced in 2024. Scenario 3: defining items for which GST rate is increased to 28% if their nutrient content is above at least one of the respective thresholds set by the World Health Organization South East Asia Region nutrient profile model [3]. Scenarios 4: adding a 12% top-up to the tax rate applied on HFSS foods and beverages in Scenario 3. DALYs: Disability-Adjusted Life Years. HFSS: High in fat, sodium, and sugar.

Table A15. Summary of lower bound results, scenarios 1 and 2

		Immediate effect at implementation		Cumulative results for the five key diseases over 2024-2053		
		Change in HH expenditure on foods and beverages (%)	Change in tax revenue from foods and beverages (%)	Change in incidence rate per 100,000 population	Change in DALYs per 100,000 population	Change in total health expenditure (USD billion)
Scenario 1	Low income	+0.6%	+51.3%	-230	-285	-10.6
	Middle income	+0.6%		-347	-1053	
	High income	+0.8%		-525	-1460	
Scenario 2	Low income	+0.9%	+92.0%	-374	-672	-17.8
	Middle income	+1.0%		-577	-1424	
	High income	+1.3%		-913	-2424	

Notes: Lower bound estimates (upper bound estimates can be found in **Table 4**). Results estimated over a 30-year period (2024-2053). Scenario 1: GST rate is increased to 28% for foods and beverages high in fat, sugar, and sodium based on the definition by the Food Safety and Standards Authority of India [2]; Scenario 2: adding a 12% top-up to the tax rate applied on HFSS foods and beverages in Scenario 1. DALYs: Disability-adjusted life years. GST: Goods and Services Tax. HFSS: High in fat, sugar, and sodium. USD: US dollar 2024.

Table A16. Summary of upper bound results, scenarios 3 and 4

		Immediate effect at implementation		Cumulative results for the five key diseases over 2024-2053		
		Change in HH expenditure on foods and beverages (%)	Change in tax revenue from foods and beverages (%)	Change in incidence rate per 100,000 population	Change in DALYs per 100,000 population	Change in total health expenditure (USD billion)
Scenario 3	Low income	+0.6%	+61.7%	-325	-752	-14.6
	Middle income	+0.6%		-490	-1256	
	High income	+0.8%		-717	-1375	
Scenario 4	Low income	+1.0%	+110.0%	-513	-1210	-23.7
	Middle income	+0.9%		-800	-2077	
	High income	+1.3%		-1188	-2776	

Notes: Upper bound estimates (lower bound estimates can be found in **Table A17**). Results estimated over a 30-year period (2024-2053). Scenario 3: scenario defining items for which GST rate is increased to 28% if their nutrient content is above at least one of the respective thresholds set by the World Health Organization South East Asia Region nutrient profile model [3]. Scenarios 4: adding a 12% top-up to the tax rate applied on HFSS foods and beverages in Scenario 3. DALYs: Disability-adjusted life years. GST: Goods and Services Tax. HFSS: High in fat, sugar, and sodium. USD: US dollar 2024.

Table A17. Summary of lower bound results, scenarios 3 and 4

		Immediate effect at implementation		Cumulative results for the five key diseases over 2024-2053		
		Change in HH expenditure on foods and beverages (%)	Change in tax revenue from foods and beverages (%)	Change in incidence rate per 100,000 population	Change in DALYs per 100,000 population	Change in total health expenditure (USD billion)
Scenario 3	Low income	+0.6%	+61.7%	-329	-654	-14.5
	Middle income	+0.6%		-477	-1122	
	High income	+0.8%		-714	-2216	
Scenario 4	Low income	+1.0%	+110.0%	-506	-1018	-23.4
	Middle income	+0.9%		-768	-1826	
	High income	+1.3%		-1190	-2696	

Notes: Lower bound estimates (upper bound estimates can be found in **Table A16**). Results estimated over a 30-year period (2024-2053). Scenario 3: scenario defining items for which GST rate is increased to 28% if their nutrient content is above at least one of the respective thresholds set by the World Health Organization South East Asia Region nutrient profile model [3]. Scenarios 4: adding a 12% top-up to the tax rate applied on HFSS foods and beverages in Scenario 3. DALYs: Disability-adjusted life years. GST: Goods and Services Tax. HFSS: High in fat, sugar, and sodium. USD: US dollar 2024.

References Appendix A

1 Ministry of Statistics and Programme Implementation. Household Consumption Expenditure Survey 2022–23 [Internet]. New Delhi: National Statistical Office, Government of India; 2024 [cited 2025 April 20]. Available from: https://microdata.gov.in/NADA/index.php/catalog/224

2 Food Safety and Standards Authority of India. Labelling & Display Amendment Draft Regulations, 2022 (44272/2022/REGULATION-FSSAI) [Internet]. New Delhi: FSSAI; 2022 [cited 2023 Jun 25]. Available from: https://fssai.gov.in/upload/uploadfiles/files/Draft_Notification_HFSS_20_09_2022.pdf

3 World Health Organization, Regional Office for South-East Asia. WHO nutrient profile model for the South-East Asia Region [Internet]. New Delhi: WHO; 2017 [cited 2023 Sep 6]. Available from: https://www.who.int/publications/i/item/9789290225447

4 Deaton A. Quality, quantity, and spatial variation of price. The American Economic Review. 1988 Jun 1:418–30.

5 Institute for Health Metrics and Evaluation. Global Burden of Disease [Internet]. Seattle (WA): IHME, University of Washington; [cited 2023 Nov 20]. Available from: https://www.healthdata.org/research-analysis/gbd
