# Supplementary material for: Taxation of foods high in fat, sugar, and sodium in India: A modelling study of health and economic impacts
Source: PLoS Med. 2026 Jan 5;23(1):e1004572. doi: 10.1371/journal.pmed.1004572 (PMC12768244; doi:10.1371/journal.pmed.1004572)
Supplement: S2 Text — Sensitivity scenarios. (PDF) [file pmed.1004572.s002.pdf]

S2 Text

Appendix B. Sensitivity scenarios

Appendix B forms part of the revised submission.

Supplement to: *Roche M, Zhu J, Olney J, Laydon DJ, Joe W, Sharma M, Steele L, Sassi F. Taxation of foods high in saturated fat, sugar, and sodium in India: A modelling study of health and economic impacts. Submitted after final revisions on 12 December 2025.*

Table of Contents

Table B1. Sensitivity scenarios definition 2
Figure B1. Sensitivity scenario: Passthrough. Immediate impact of fiscal policy scenarios on average daily energy and nutrient intake, by income group 3
Figure B2. Sensitivity scenario: Passthrough. Impact of fiscal policy scenarios on average total household expenditure on food and beverages, by income group 4
Figure B3. Sensitivity scenario: Passthrough. Impact of fiscal policy scenarios on government tax revenue from foods and beverages 5
Figure B4. Sensitivity scenario: Reformulation. Immediate impact of fiscal policy scenarios on average daily energy and nutrient intake, by income group 6
Figure B5. Sensitivity scenario: Reformulation. Impact of fiscal policy scenarios on average total household expenditure on food and beverages, by income group 7
Figure B6. Sensitivity scenario: Reformulation. Impact of fiscal policy scenarios on government tax revenue from foods and beverages 8
Figure B7. Sensitivity scenario: Subsidy. Immediate impact of fiscal policy scenarios on average daily energy and nutrient intake, by income group 9
Figure B8. Sensitivity scenario: Subsidy. Impact of fiscal policy scenarios on average total household expenditure on food and beverages, by income group 10
Figure B9. Sensitivity scenario: Subsidy. Impact of fiscal policy scenarios on government tax revenue from foods and beverages 11
References Appendix B 12

Table B1. Sensitivity scenarios definition

**Name of sensitivity scenario**	**Definition of scenario**
Passthrough	Tax under-shifting with a uniform tax passthrough of 80%, based on the meta-analysis of SSB tax evaluation studies by Andreyeva et al (2022) [1].<br><br>Example: If 0% GST rate originally applies on an HFSS item priced at $1·00. After the policy change, the GST rate applied will be increased to 28%, but the price of the item will only increase by 28% * 80% = 22·4%, leading to a price of $1·22 (vs. $1·28 under full tax passthrough).
Reformulation	If the nutrient content of an HFSS item is less than 50% above the FSSAI HFSS [2] or WHO SEARO NPM [3] threshold for one or several nutrients (among sugar, sodium, and saturated fat), the industry is assumed to reduce the nutrient content of the item for it not to be considered HFSS and avoid the tax. This reformulation is modelled to take place before the introduction of the tax.<br><br>Example: Under the FSSAI HFSS definition [2], if an item contains 1·2mg of sodium per kcal, we assume that the industry would reformulate the item to 1·0mg/kcal to avoid the tax.<br><br>Further assumptions:<br>- If the sugar content of an item is reduced to meet the HFSS definition threshold, its energy content is proportionally reduced (following the 1g = 4 kcal conversion rate)<br>- If the total fat content of an item is reduced to meet the HFSS definition threshold, its energy content is proportionally reduced (following the 1g = 9 kcal conversion rate)<br>- If the saturated fat content of an item is reduced to meet the HFSS definition threshold, it is assumed to be replaced by other forms of fat and thus its energy content is not reduced<br>- If the energy content of an item is reduced to meet the HFSS threshold, and the sugar, saturated fat, and total fat content are below their respective HFSS threshold, the content of sugar, saturated fat, and total fat is assumed not to be modified
Subsidy	GST rate of 0% on F&V and pulses.

Notes: F&V: fruits & vegetables, FSSAI: Food Safety and Standards Authority of India, HFSS: high in fat, sugar, and sodium, GST: Goods and Services Tax, NPM: nutrient profile model, SSB: sugar-sweetened beverages, WHO SEARO: World Health Organization South East Asia Region. Each of the four main scenarios of the analysis are re-simulated under the three assumptions listed in the table.

Figure B1. Sensitivity scenario: Passthrough. Immediate impact of fiscal policy scenarios on average daily energy and nutrient intake, by income group

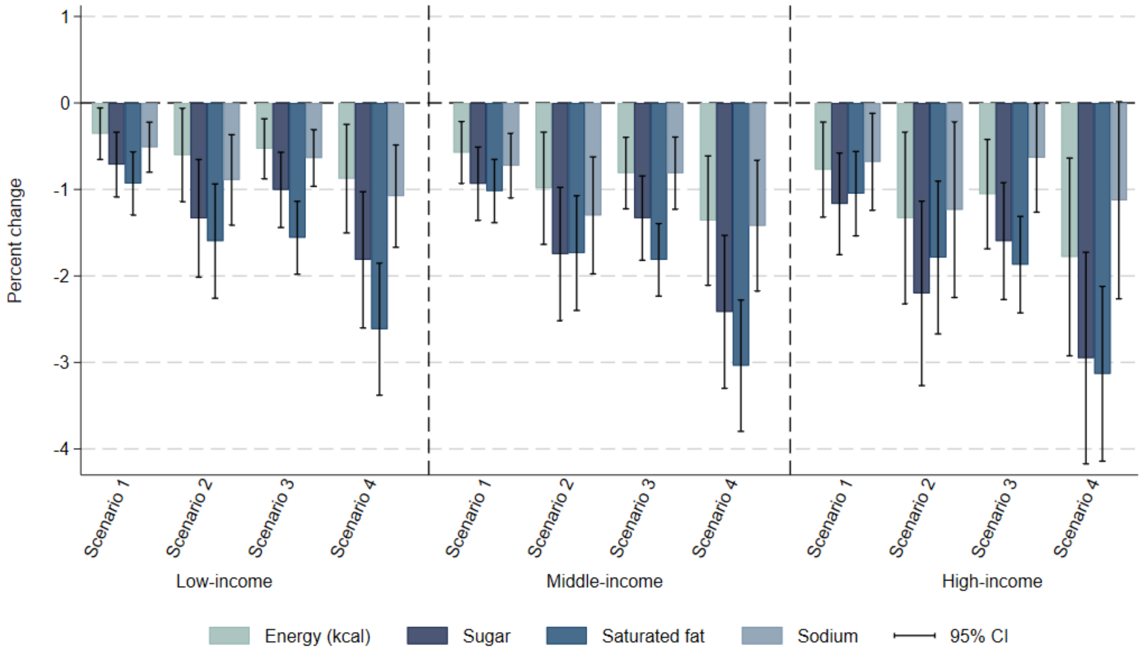

Notes: This is a sensitivity analysis using an incomplete passthrough of tax rate changes to prices of 80%. Vertical segments represent the 95% confidence intervals. Survey weighted. Scenario 1: defining items for which GST rate is increased to 28% based on the definition of foods and beverages high in fat, sugar, and sodium by the Food Safety and Standards Authority of India [2]; Scenario 2: adding a 12% top-up to the tax rate applied on HFSS foods and beverages in Scenario 1. Scenario 3: scenario defining items for which GST rate is increased to 28% if their nutrient content is above at least one of the respective thresholds set by the World Health Organization South East Asia Region nutrient profile model [3]; Scenarios 4: adding a 12% top-up to the tax rate applied on HFSS foods and beverages in Scenario 3. GST: Goods and Services Tax. HFSS: High in fat, sodium, and sugar.

Figure B2. Sensitivity scenario: Passthrough. Impact of fiscal policy scenarios on average total household expenditure on food and beverages, by income group

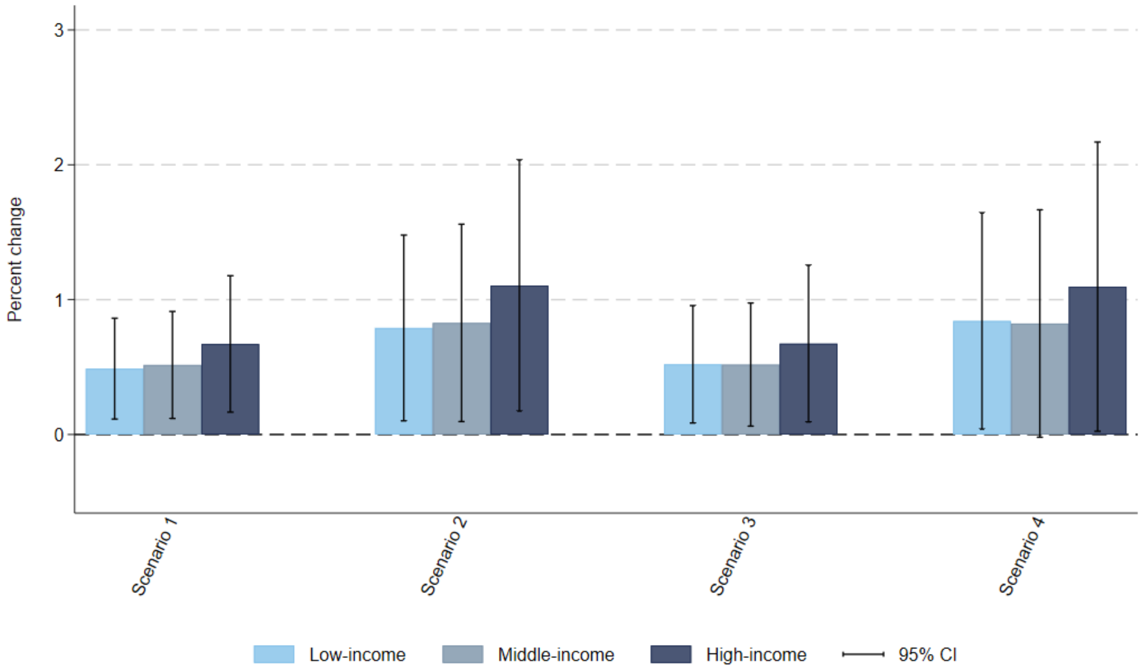

Notes: This is a sensitivity analysis using an incomplete passthrough of tax rate changes to prices of 80%. Vertical segments represent the 95% confidence intervals. Survey weighted. Scenario 1: defining items for which GST rate is increased to 28% based on the definition of foods and beverages high in fat, sugar, and sodium by the Food Safety and Standards Authority of India [2]; Scenario 2: adding a 12% top-up to the tax rate applied on HFSS foods and beverages in Scenario 1. Scenario 3: scenario defining items for which GST rate is increased to 28% if their nutrient content is above at least one of the respective thresholds set by the World Health Organization South East Asia Region nutrient profile model [3]; Scenarios 4: adding a 12% top-up to the tax rate applied on HFSS foods and beverages in Scenario 3. GST: Goods and Services Tax. HFSS: High in fat, sodium, and sugar.

Figure B3. Sensitivity scenario: Passthrough. Impact of fiscal policy scenarios on government tax revenue from foods and beverages

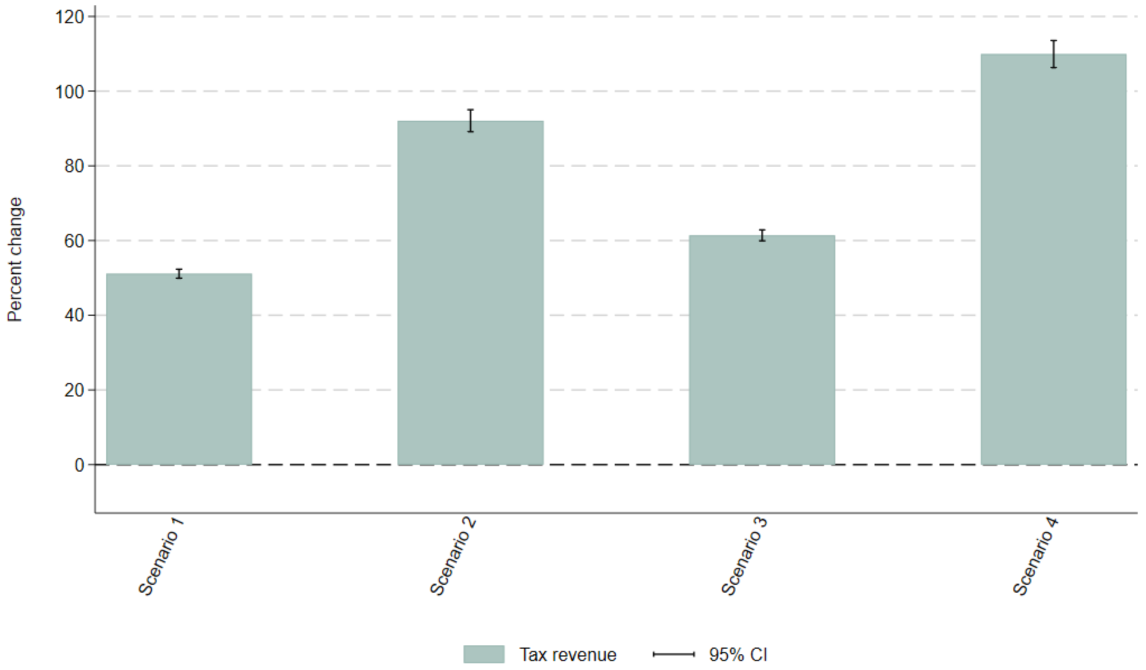

Notes: This is a sensitivity analysis using an incomplete passthrough of tax rate changes to prices of 80%. Vertical segments represent the 95% confidence intervals. Survey weighted. Scenario 1: defining items for which GST rate is increased to 28% based on the definition of foods and beverages high in fat, sugar, and sodium by the Food Safety and Standards Authority of India [2]; Scenario 2: adding a 12% top-up to the tax rate applied on HFSS foods and beverages in Scenario 1. Scenario 3: scenario defining items for which GST rate is increased to 28% if their nutrient content is above at least one of the respective thresholds set by the World Health Organization South East Asia Region nutrient profile model [3]; Scenarios 4: adding a 12% top-up to the tax rate applied on HFSS foods and beverages in Scenario 3. GST: Goods and Services Tax. HFSS: High in fat, sodium, and sugar.

Figure B4. Sensitivity scenario: Reformulation. Immediate impact of fiscal policy scenarios on average daily energy and nutrient intake, by income group

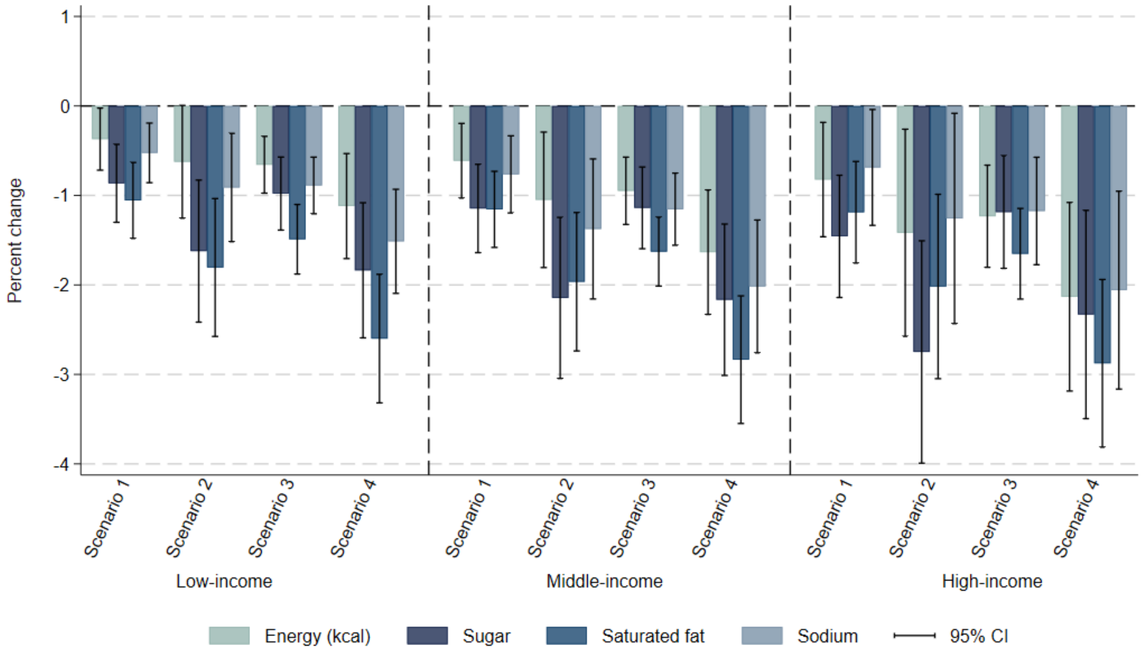

Notes: This is a sensitivity analysis assuming the reformulation of HFSS food items by the industry. If the nutrient content of an HFSS item is less than 50% above the FSSAI HFSS or WHO SEARO NPM threshold for one or several nutrients (among sugar, sodium, and saturated fat), the industry is assumed to reduce the nutrient content of the item for it not to be considered HFSS and avoid the tax. This reformulation is modeled to take place before the introduction of the tax. Vertical segments represent the 95% confidence intervals. Survey weighted. Scenario 1: defining items for which GST rate is increased to 28% based on the definition of foods and beverages high in fat, sugar, and sodium by the Food Safety and Standards Authority of India [2]; Scenario 2: adding a 12% top-up to the tax rate applied on HFSS foods and beverages in Scenario 1. Scenario 3: scenario defining items for which GST rate is increased to 28% if their nutrient content is above at least one of the respective thresholds set by the World Health Organization South East Asia Region nutrient profile model [3]; Scenarios 4: adding a 12% top-up to the tax rate applied on HFSS foods and beverages in Scenario 3. GST: Goods and Services Tax. HFSS: High in fat, sodium, and sugar.

Figure B5. Sensitivity scenario: Reformulation. Impact of fiscal policy scenarios on average total household expenditure on food and beverages, by income group

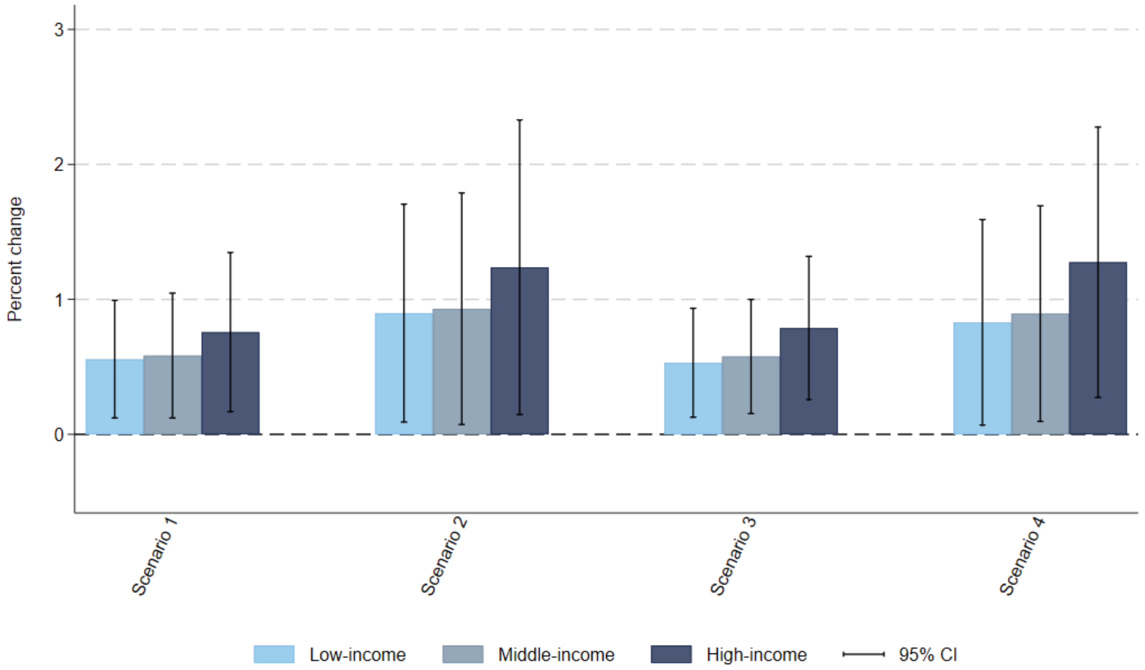

Notes: This is a sensitivity analysis assuming the reformulation of HFSS food items by the industry. If the nutrient content of an HFSS item is less than 50% above the FSSAI HFSS or WHO SEARO NPM threshold for one or several nutrients (among sugar, sodium, and saturated fat), the industry is assumed to reduce the nutrient content of the item for it not to be considered HFSS and avoid the tax. This reformulation is modeled to take place before the introduction of the tax. Vertical segments represent the 95% confidence intervals. Survey weighted. Scenario 1: defining items for which GST rate is increased to 28% based on the definition of foods and beverages high in fat, sugar, and sodium by the Food Safety and Standards Authority of India [2]; Scenario 2: adding a 12% top-up to the tax rate applied on HFSS foods and beverages in Scenario 1. Scenario 3: scenario defining items for which GST rate is increased to 28% if their nutrient content is above at least one of the respective thresholds set by the World Health Organization South East Asia Region nutrient profile model [3]; Scenarios 4: adding a 12% top-up to the tax rate applied on HFSS foods and beverages in Scenario 3. GST: Goods and Services Tax. HFSS: High in fat, sodium, and sugar.

Figure B6. Sensitivity scenario: Reformulation. Impact of fiscal policy scenarios on government tax revenue from foods and beverages

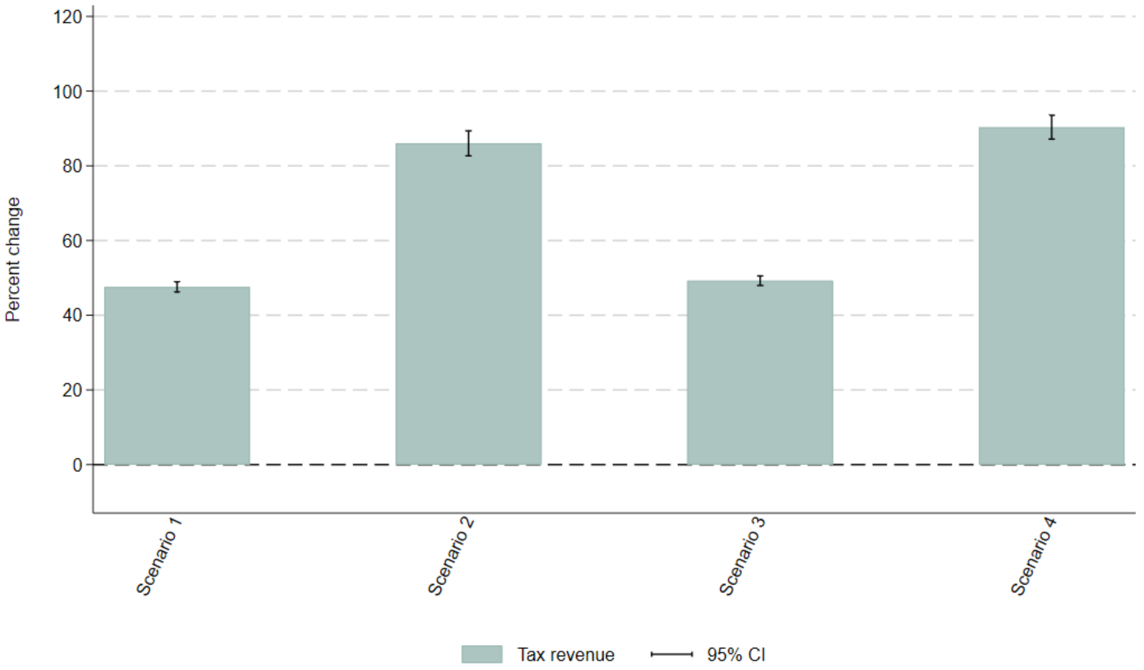

Notes: This is a sensitivity analysis assuming the reformulation of HFSS food items by the industry. If the nutrient content of an HFSS item is less than 50% above the FSSAI HFSS or WHO SEARO NPM threshold for one or several nutrients (among sugar, sodium, and saturated fat), the industry is assumed to reduce the nutrient content of the item for it not to be considered HFSS and avoid the tax. This reformulation is modeled to take place before the introduction of the tax. Vertical segments represent the 95% confidence intervals. Survey weighted. Scenario 1: defining items for which GST rate is increased to 28% based on the definition of foods and beverages high in fat, sugar, and sodium by the Food Safety and Standards Authority of India [2]; Scenario 2: adding a 12% top-up to the tax rate applied on HFSS foods and beverages in Scenario 1. Scenario 3: scenario defining items for which GST rate is increased to 28% if their nutrient content is above at least one of the respective thresholds set by the World Health Organization South East Asia Region nutrient profile model [3]; Scenarios 4: adding a 12% top-up to the tax rate applied on HFSS foods and beverages in Scenario 3. GST: Goods and Services Tax. HFSS: High in fat, sodium, and sugar.

Figure B7. Sensitivity scenario: Subsidy. Immediate impact of fiscal policy scenarios on average daily energy and nutrient intake, by income group

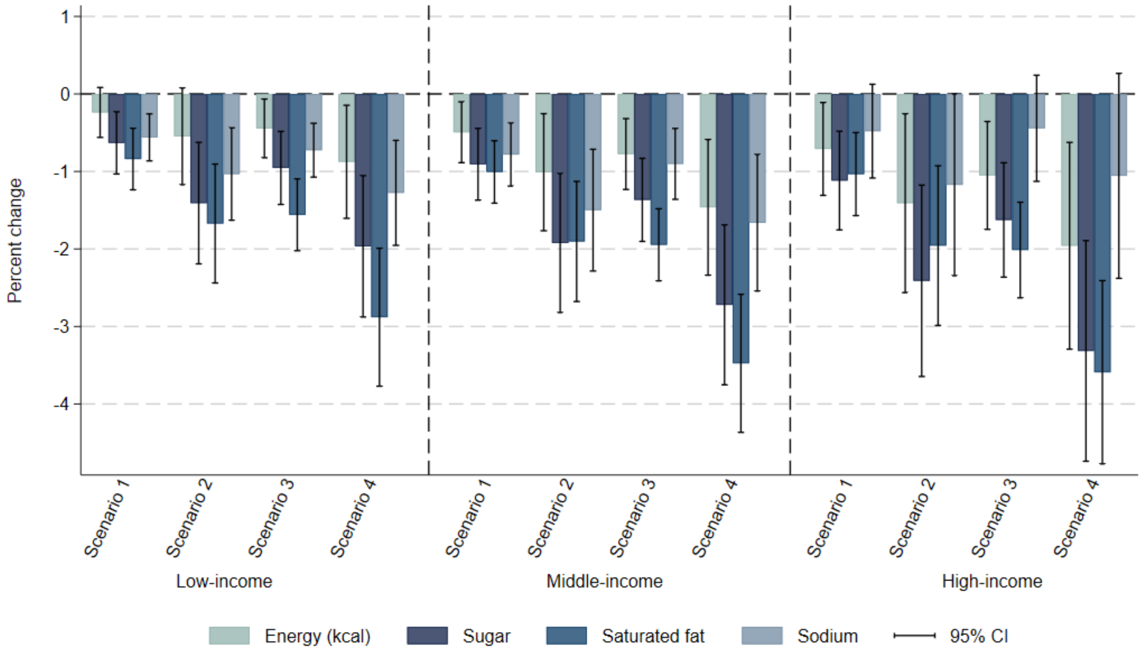

Notes: This is a sensitivity analysis additionally zero-rating F&V and pulses (GST rate = 0%). Most F&V are already zero-rated at baseline so most of the subsidy effect comes from pulses of which many are taxed with a GST rate of 5% at baseline (**Figure A1**). Vertical segments represent the 95% confidence intervals. Survey weighted. Scenario 1: defining items for which GST rate is increased to 28% based on the definition of foods and beverages high in fat, sugar, and sodium by the Food Safety and Standards Authority of India [2]; Scenario 2: adding a 12% top-up to the tax rate applied on HFSS foods and beverages in Scenario 1. Scenario 3: scenario defining items for which GST rate is increased to 28% if their nutrient content is above at least one of the respective thresholds set by the World Health Organization South East Asia Region nutrient profile model [3]; Scenarios 4: adding a 12% top-up to the tax rate applied on HFSS foods and beverages in Scenario 3. GST: Goods and Services Tax. HFSS: High in fat, sodium, and sugar.

Figure B8. Sensitivity scenario: Subsidy. Impact of fiscal policy scenarios on average total household expenditure on food and beverages, by income group

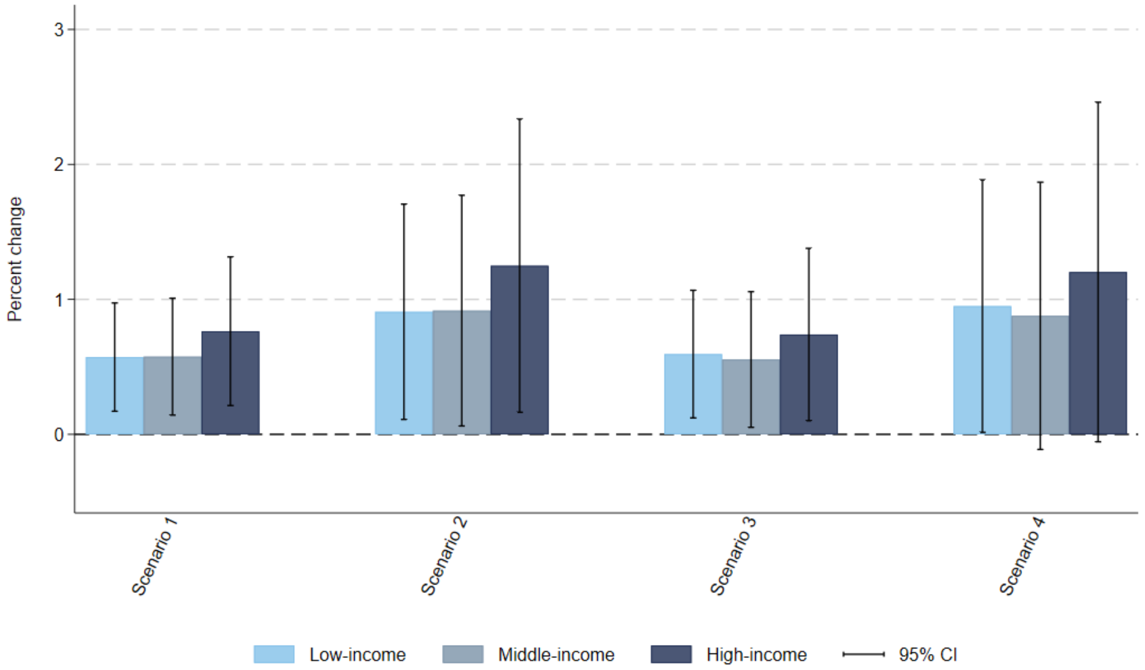

Notes: This is a sensitivity analysis additionally zero-rating F&V and pulses (GST rate = 0%). Most F&V are already zero-rated at baseline so most of the subsidy effect comes from pulses of which many are taxed with a GST rate of 5% at baseline (**Figure A1**). Vertical segments represent the 95% confidence intervals. Survey weighted. Scenario 1: defining items for which GST rate is increased to 28% based on the definition of foods and beverages high in fat, sugar, and sodium by the Food Safety and Standards Authority of India [2]; Scenario 2: adding a 12% top-up to the tax rate applied on HFSS foods and beverages in Scenario 1. Scenario 3: scenario defining items for which GST rate is increased to 28% if their nutrient content is above at least one of the respective thresholds set by the World Health Organization South East Asia Region nutrient profile model [3]; Scenarios 4: adding a 12% top-up to the tax rate applied on HFSS foods and beverages in Scenario 3. GST: Goods and Services Tax. HFSS: High in fat, sodium, and sugar.

Figure B9. Sensitivity scenario: Subsidy. Impact of fiscal policy scenarios on government tax revenue from foods and beverages

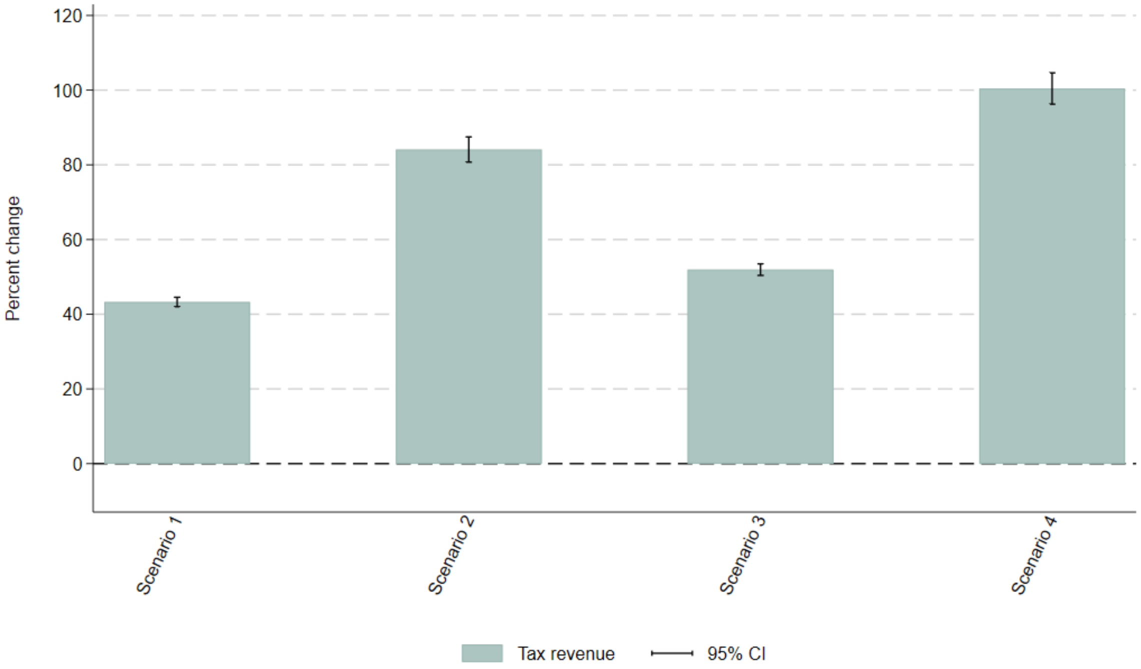

Notes: This is a sensitivity analysis additionally zero-rating F&V and pulses (GST rate = 0%). Most F&V are already zero-rated at baseline so most of the subsidy effect comes from pulses of which many are taxed with a GST rate of 5% at baseline (**Figure A1**). Vertical segments represent the 95% confidence intervals. Survey weighted. Scenario 1: defining items for which GST rate is increased to 28% based on the definition of foods and beverages high in fat, sugar, and sodium by the Food Safety and Standards Authority of India [2]; Scenario 2: adding a 12% top-up to the tax rate applied on HFSS foods and beverages in Scenario 1. Scenario 3: scenario defining items for which GST rate is increased to 28% if their nutrient content is above at least one of the respective thresholds set by the World Health Organization South East Asia Region nutrient profile model [3]; Scenarios 4: adding a 12% top-up to the tax rate applied on HFSS foods and beverages in Scenario 3. GST: Goods and Services Tax. HFSS: High in fat, sodium, and sugar.

References Appendix B

1 Andreyeva T, Marple K, Marinello S, Moore TE, Powell LM. Outcomes following taxation of sugar-sweetened beverages: a systematic review and meta-analysis. JAMA Network Open. 2022 Jun 1;5(6):e2215276–.

2 Food Safety and Standards Authority of India. Labelling & Display Amendment Draft Regulations, 2022 (44272/2022/REGULATION-FSSAI) [Internet]. New Delhi: FSSAI; 2022 [cited 2023 Jun 25]. Available from: https://fssai.gov.in/upload/uploadfiles/files/Draft_Notification_HFSS_20_09_2022.pdf

3 World Health Organization, Regional Office for South-East Asia. WHO nutrient profile model for the South-East Asia Region [Internet]. New Delhi: WHO; 2017 [cited 2023 Sep 6]. Available from: https://www.who.int/publications/i/item/9789290225447
