# Supplementary material for: Taxation of foods high in fat, sugar, and sodium in India: A modelling study of health and economic impacts
Source: PLoS Med. 2026 Jan 5;23(1):e1004572. doi: 10.1371/journal.pmed.1004572 (PMC12768244; doi:10.1371/journal.pmed.1004572)
Supplement: S3 Text — Almost Ideal Demand System (AIDS) demand model. (PDF) [file pmed.1004572.s003.pdf]

S3 Text

Appendix C. Almost Ideal Demand System (AIDS) demand model

Appendix C forms part of the revised submission.

Supplement to: *Roche M, Zhu J, Olney J, Laydon DJ, Joe W, Sharma M, Steele L, Sassi F. Taxation of foods high in saturated fat, sugar, and sodium in India: A modelling study of health and economic impacts. Submitted after final revisions on 12 December 2025.*

Table of Contents

C1. The model 2
C.2. Food groups 3
Table C1. Estimated average income elasticity estimates, by income group 4
Figure C1. Estimated average income elasticity estimates, by income group 5
Table C2. Robustness: Estimated average price elasticities using aggregated beverage grouping, full sample 6
Table C3. Robustness: Estimated average price elasticities using non-food CPI as unit value for numeraire, full sample 7
References Appendix C 8

C1. The model

As with most other household budget surveys, the NSSO Household Consumption Expenditure survey 2023-24 does not provide price information, thus we rely on unit values (i.e., expenditure divided by quantity). However, unit values are subject to measurement errors in both expenditure and quantity and may lead to quality shading, where consumers may not only adjust quantity but also the quality of the goods they buy as a response to price changes. We thus estimate consumer responses to food price changes (technically, own- and cross-price elasticities of demand) using Deaton (1988)'s Almost Ideal Demand System (AIDS) model adjusting for quality shading and measurement error [1].

$$w_{jic} = \alpha_j^0 + \beta_j^0 \ln x_{ic} + \gamma_j^0 \boldsymbol{z_{ic}} + \sum_k \theta_{jk} \ln p_{kc} + f_{jc} + u_{jic}^0 \quad (1)$$

$$\ln v_{jic} = \alpha_j^1 + \beta_j^1 \ln x_{ic} + \gamma_j^1 \boldsymbol{z_{ic}} + \sum_k \psi_{jk} \ln p_{kc} + u_{jic}^1 \quad (2)$$

where w_{jic} represents the share of total expenditure on food and beverages (x_{ic}) that household *i* in cluster *c* spends on good j; p_{kc} is the price of good k which does not vary in cluster *c*; f_{jc} are unobservable cluster fixed effects; v_{jic} is the unit value of good j; and u_{jic} are idiosyncratic error terms. $\boldsymbol{z_{ic}}$ is a vector of household characteristics, including the logarithm of household size, the sector of the household (urban vs. rural), the sex of the head of household and a dummy for completion of primary education, and dummies capturing the religion of the household. We remove regional and seasonal effects using dummies for the six Indian administrative zones and the quarter of the year in which the household reports information.

The specification nests rich income (Engel-curve) responses and flexible cross-price substitution patterns while remaining consistent with standard consumer theory. Adding-up, homogeneity, and Slutsky symmetry are imposed so the estimates are integrable and interpretable as arising from well-behaved preferences in the price-independent generalized logarithmic preferences class (PIGLOG) [2].

The model assumes spatially varying prices, where all households within a near geographical area face the same price. As defined by NSSO, a cluster is equivalent to a village, or an urban block, surveyed during the same period. Any within-cluster variation in unit values is due to differences in the quality of the purchased items. Spatial variations in unit values between clusters provide an identification strategy to avoid the endogeneity of prices and address quality shading [1]. We argue this assumption is justified in a middle-income country like India where transport is more difficult and relatively costlier, and markets are not always well-integrated [2]. To abide by our main assumption of spatially varying prices, we force households to face the same price if they are drawn from the same cluster, regardless of their income group. This takes care of a potential endogeneity in average prices that might arise if households from different income groups face different average prices despite being in the same cluster [3]. We drop clusters not having at least two households consuming at least an item for each group. Following the demand system literature [4], we normalize the *numeraire* group's unit value to one.

We recover uncompensated price elasticity of demand estimates at the means through a three-step method including demeaned within-cluster regressions, between-cluster regressions of adjusted budget share on adjusted unit values estimated in the first step using an error-in-variable estimator, and finally separating quality and price effects.

$$\varepsilon_{jk} = \frac{\hat{\theta}_{jk}}{\overline{w}_j} - \delta_{jk} \quad (3)$$

where δ_{jk} is the Kronecker delta equal to one if $j = k$ (i.e., for own-price elasticities) or zero otherwise.

This approach has been used extensively to model the demand for food in low- and middle-income countries [5]. A key strength is its rigorous treatment of measurement noise. By residualizing within clusters, averaging to the

cluster, and then applying measurement-error corrections that scale with buyer and household counts, it strips out spurious variation that would otherwise attenuate price effects while also tempering quality shading.

Nevertheless, the use of this model with NSSO Household Consumption Expenditure survey data has some limitations. First, censoring is only mitigated, not solved. Unit-value regressions exclude non-buyers while budget share regressions include everyone. Clusters with very few buyers are dropped to avoid unstable means and the measurement-error corrections down-weight clusters where buyer counts are small. These steps reduce the distortion created by many zeros, but they cannot remove selection bias if the decision not to buy is correlated with unobserved preferences, access, or local conditions that also relate to prices. Second, using household budget survey data, the underlying demand model cannot account for cross-price effects within food groups. Future research using detailed consumer panel data (e.g., home-scan) could estimate demand systems using more disaggregated groups, for example, by bundling items based on nutritional impact. Third, the NSSO also lacks information on household disposable income, which is commonly used as an instrumental variable to address the endogeneity in total expenditure arising from simultaneity bias [6].

Details regarding the microeconomic foundations of Deaton (1988)'s AIDS model, as well as its derivation and the steps involved in its estimation, have been described elsewhere [2]. The paper uses uncompensated price elasticities as opposed to compensated price elasticities, as uncompensated elasticities measure the effect of a price change for one good while keeping income and the prices of other goods constant, thereby reflecting both income and substitution effects.

C.2. Food groups

We estimate the model parameters for eleven groups: cereals, dairy, pulses, edible oils and spices (including salt and raw sugar), fruits and vegetables (including nuts, hereafter referred to as F&V), animal meats (fresh), packaged processed foods, sweets, SSBs, non-SSBs, and a numeraire group containing food-away-from-home and non-food expenditure. While we account for served processed foods (on-trade sector, i.e., food-away-from-home, like bars, restaurants, etc.) in the estimation of total nutrient intake, we do not include this aggregate as a standalone food group in our demand system. Instead, we group it with non-food expenditure within the *numeraire*.

The GST is applied differently to the on-trade sector in India, based on industry categorisation or types of premises rather than the specific type of food or beverage items served. Therefore, the GST scenarios simulated in this study only apply to the off-trade sector. In addition, food-away-from-home is not well captured in the NSS Household Consumption Expenditure survey, including only a limited number of broadly defined items (cooked meals purchased, cooked meals received free in the workplace, cooked meals received as assistance, cooked snacks purchased, other served processed food) [7]. While the simulated GST scenarios may impact the price of raw materials, it is not possible, given the available data, to estimate the extent to which this may affect the price of served processed foods.

Table C1. Estimated average income elasticity estimates, by income group

	Low-income	Middle-income	High-income
Cereals	0.329***	0.271***	0.122***
	(0.015)	(0.016)	(0.006)
Dairy	1.175***	0.752***	0.496***
	(0.017)	(0.018)	(0.007)
Pulses	0.450***	0.386***	0.281***
	(0.012)	(0.013)	(0.006)
Oils & spices	0.404***	0.327***	0.229***
	(0.008)	(0.010)	(0.005)
F&V	0.564***	0.470***	0.344***
	(0.009)	(0.011)	(0.005)
Animal meats, fresh	0.980***	0.734***	0.477***
	(0.017)	(0.022)	(0.008)
Packaged proc. foods	1.076***	0.861***	0.610***
	(0.015)	(0.017)	(0.008)
Sweets	0.842***	0.846***	0.665***
	(0.018)	(0.025)	(0.011)
SSBs	1.962***	1.560***	1.162***
	(0.042)	(0.048)	(0.020)
Non-SSBs	1.093***	0.992***	0.867***
	(0.022)	(0.028)	(0.013)

Notes: Estimated based on NSS Household Consumption Expenditure survey 2022-23 [8] and using Deaton (1988)'s quality-adjusted Almost Ideal Demand System model [1]. Bootstrapped standard errors after 300 replications. Vertical segments represent the 95% confidence intervals. Pack.: Packaged; proc.: processed; SSBs: sugar-sweetened beverages.

Figure C1. Estimated average income elasticity estimates, by income group

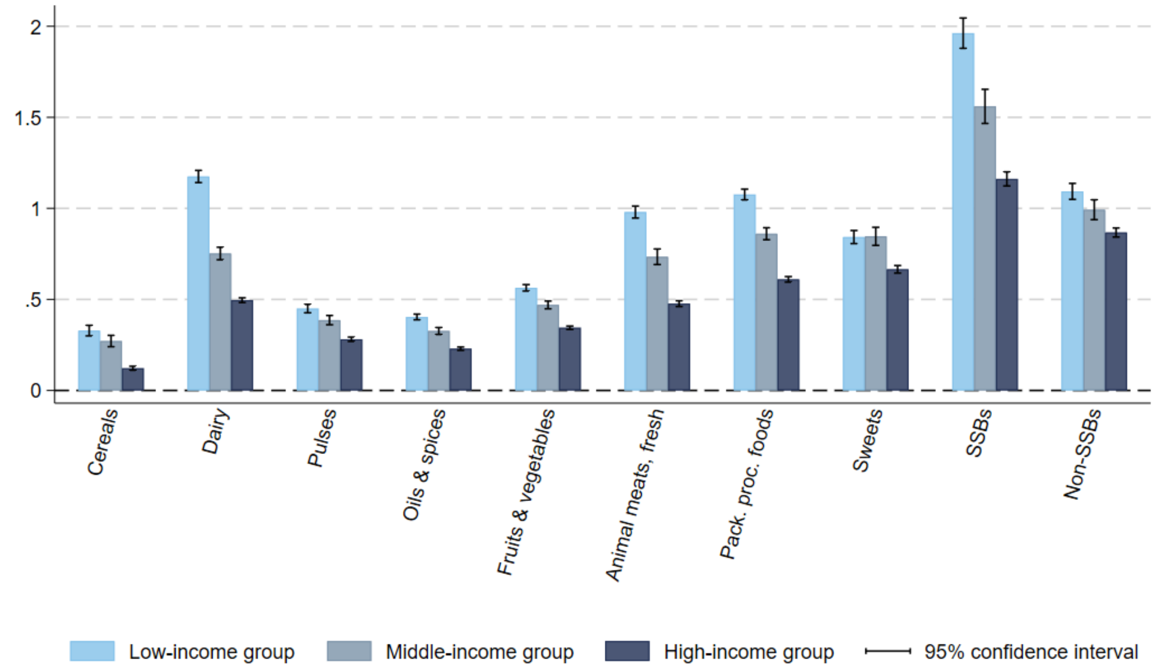

Notes: Estimated based on NSS Household Consumption Expenditure survey 2022-23 [8] and using Deaton (1988)'s quality-adjusted Almost Ideal Demand System model [1]. Bootstrapped standard errors after 300 replications. Vertical segments represent the 95% confidence intervals. Pack.: Packaged; proc.: processed; SSBs: sugar-sweetened beverages.

Table C2. Robustness: Estimated average price elasticities using aggregated beverage grouping, full sample

	Cereals	Dairy	Pulses	Oils & spices	F&V	Animal meats, fresh	Packaged proc. foods	Sweets	SSBs
Cereals	**-0.768*****	-0.054***	-0.002	-0.016***	0.042***	0.012**	-0.016***	0.008*	0.038***
	**(0.003)**	(0.006)	(0.002)	(0.004)	(0.004)	(0.006)	(0.004)	(0.004)	(0.004)
Dairy	-0.028***	**-1.141*****	-0.014***	-0.017***	0.000	0.069***	0.044***	0.037***	0.058***
	(0.002)	**(0.005)**	(0.002)	(0.004)	(0.003)	(0.005)	(0.004)	(0.004)	(0.004)
Pulses	0.000	-0.031***	**-0.781*****	-0.101***	-0.230***	0.339***	-0.091***	-0.046***	0.041***
	(0.003)	(0.008)	**(0.013)**	(0.013)	(0.008)	(0.016)	(0.009)	(0.009)	(0.005)
Oils & spices	-0.002	0.008**	-0.029***	**-0.327*****	0.060***	0.076***	0.010*	-0.023***	0.110***
	(0.002)	(0.004)	(0.004)	**(0.007)**	(0.004)	(0.007)	(0.005)	(0.006)	(0.003)
F&V	0.012***	0.010***	-0.070***	0.039***	**-0.632*****	-0.134***	-0.015***	0.006	-0.002
	(0.002)	(0.003)	(0.002)	(0.004)	**(0.004)**	(0.005)	(0.004)	(0.004)	(0.003)
Animal meats, fresh	-0.001	0.127***	0.163***	0.100***	-0.253***	**-0.625*****	0.286***	-0.010	0.017**
	(0.004)	(0.009)	(0.008)	(0.013)	(0.009)	**(0.026)**	(0.011)	(0.011)	(0.008)
Packaged proc. foods	-0.032***	0.125***	-0.084***	-0.011	-0.065***	0.460***	**-0.966*****	0.032***	-0.021***
	(0.004)	(0.012)	(0.007)	(0.015)	(0.011)	(0.018)	**(0.009)**	(0.007)	(0.005)
Sweets	-0.004	0.123***	-0.057***	-0.125***	-0.005	-0.025	0.038***	**-0.388*****	-0.084***
	(0.006)	(0.016)	(0.009)	(0.019)	(0.014)	(0.022)	(0.009)	**(0.015)**	(0.008)
SSBs	0.017***	0.108***	0.013***	0.162***	-0.020***	0.018**	-0.011***	-0.041***	**-1.506*****
	(0.003)	(0.008)	(0.003)	(0.006)	(0.005)	(0.008)	(0.003)	(0.004)	**(0.007)**

Notes: Sample size: 247,368 households in 7,146 clusters. Estimated based on NSS Household Consumption Expenditure survey 2022-23 [7] and using Deaton (1988)'s quality-adjusted Almost Ideal Demand System model [1]. Own-price elasticities and their standard errors are denoted in bold. Bootstrapped standard errors after 300 replications. Pack.: Packaged; proc.: processed; F&V: fruits & vegetables; SSBs: sugar-sweetened beverages. * p<0·1, ** p<0·05, *** p<0·01.

Table C3. Robustness: Estimated average price elasticities using non-food CPI as unit value for numeraire, full sample

	Cereals	Dairy	Pulses	Oils & spices	F&V	Animal meats, fresh	Packaged proc. foods	Sweets	SSBs	Non-SSBs
Cereals	**-0.797*****	-0.088***	0.007***	-0.026***	0.049***	0.023***	-0.028***	0.022***	0.015**	0.004
	**(0.004)**	(0.007)	(0.003)	(0.005)	(0.004)	(0.007)	(0.004)	(0.005)	(0.006)	(0.004)
Dairy	-0.038***	**-1.124*****	-0.010***	-0.003	-0.024***	0.072***	0.050***	0.057***	0.014**	0.071***
	(0.002)	**(0.006)**	(0.002)	(0.003)	(0.003)	(0.006)	(0.004)	(0.005)	(0.007)	(0.004)
Pulses	0.013***	-0.017*	**-0.748*****	-0.143***	-0.170***	0.284***	-0.071***	-0.032***	0.001	0.036***
	(0.004)	(0.009)	**(0.013)**	(0.016)	(0.011)	(0.019)	(0.009)	(0.010)	(0.009)	(0.004)
Oils & spices	-0.006***	0.023***	-0.042***	**-0.367*****	0.061***	0.070***	0.005	-0.031***	0.016**	0.075***
	(0.002)	(0.004)	(0.005)	**(0.009)**	(0.004)	(0.008)	(0.006)	(0.006)	(0.006)	(0.003)
F&V	0.014***	-0.017***	-0.051***	0.037***	**-0.642*****	-0.127***	-0.017***	0.003	0.007	0.006**
	(0.002)	(0.003)	(0.003)	(0.004)	**(0.005)**	(0.006)	(0.004)	(0.005)	(0.005)	(0.003)
Animal meats, fresh	0.006	0.131***	0.128***	0.085***	-0.236***	**-0.630*****	0.265***	-0.013	-0.024**	0.025***
	(0.005)	(0.011)	(0.009)	(0.012)	(0.010)	**(0.030)**	(0.013)	(0.014)	(0.012)	(0.007)
Packaged proc. foods	-0.042***	0.138***	-0.063***	-0.024	-0.067***	0.414***	**-0.922*****	0.002	0.035***	-0.011**
	(0.005)	(0.013)	(0.007)	(0.016)	(0.011)	(0.020)	**(0.011)**	(0.008)	(0.008)	(0.005)
Sweets	0.013**	0.192***	-0.040***	-0.141***	-0.017	-0.031	0.001	**-0.439*****	0.030***	-0.073***
	(0.006)	(0.019)	(0.010)	(0.019)	(0.016)	(0.028)	(0.010)	**(0.019)**	(0.010)	(0.008)
SSBs	0.020	0.080	-0.014	0.046	0.002	-0.124**	0.087***	0.059***	**-1.203*****	0.090***
	(0.018)	(0.058)	(0.018)	(0.043)	(0.035)	(0.052)	(0.021)	(0.022)	**(0.017)**	(0.020)
Non-SSBs	-0.001	0.195***	0.020***	0.151***	0.016**	0.042***	-0.002	-0.045***	0.036***	**-1.474*****
	(0.004)	(0.011)	(0.003)	(0.007)	(0.007)	(0.010)	(0.005)	(0.005)	(0.006)	**(0.009)**

Notes: Estimated based on NSS Household Consumption Expenditure survey 2022-23 [7] and using Deaton (1988)'s quality-adjusted Almost Ideal Demand System model [1]. Own-price elasticities and their standard errors are denoted in bold. Bootstrapped standard errors after 300 replications. Pack.: Packaged; proc.: processed; F&V: fruits & vegetables; SSBs: sugar-sweetened beverages. * p<0·1, ** p<0·05, *** p<0·01.

References Appendix C

1 Deaton A. Quality, quantity, and spatial variation of price. The American Economic Review. 1988 Jun 1:418–30.

2 Deaton A. The Analysis of Household Surveys: A Microeconometric Approach to Development Policy [Internet]. Washington, D.C.: World Bank; 2019 [cited 2023 Dec 19]. Available from: http://hdl.handle.net/10986/30394

3 John RM, Tullu FT, Gupta R. Price elasticity and affordability of aerated or sugar-sweetened beverages in India: implications for taxation. BMC Public Health. 2022 Jul 17;22(1):1372.

4 Richards TJ, Padilla L. Promotion and fast food demand. American Journal of Agricultural Economics. 2009 Feb;91(1):168-83.

5 Green R, Cornelsen L, Dangour AD, Turner R, Shankar B, Mazzocchi M, Smith RD. The effect of rising food prices on food consumption: systematic review with meta-regression. BMJ. 2013 Jun 17;346.

6 Banks J, Blundell R, Lewbel A. Quadratic Engel curves and consumer demand. Review of Economics and statistics. 1997 Nov 1;79(4):527–39.

7 Ministry of Statistics and Programme Implementation. Household Consumption Expenditure Survey 2022–23 [Internet]. New Delhi: National Statistical Office, Government of India; 2024 [cited 2025 April 20]. Available from: https://microdata.gov.in/NADA/index.php/catalog/224
