# Supplementary material for: Taxation of foods high in fat, sugar, and sodium in India: A modelling study of health and economic impacts
Source: PLoS Med. 2026 Jan 5;23(1):e1004572. doi: 10.1371/journal.pmed.1004572 (PMC12768244; doi:10.1371/journal.pmed.1004572)
Supplement: S4 Text — Health-GPS microsimulation model. (PDF) [file pmed.1004572.s004.pdf]

S4 Text

Appendix D. Health-GPS microsimulation model

Appendix D forms part of the revised submission.

Supplement to: *Roche M, Zhu J, Olney J, Laydon DJ, Joe W, Sharma M, Steele L, Sassi F. Taxation of foods high in saturated fat, sugar, and sodium in India: A modelling study of health and economic impacts. Submitted after final revisions on 12 December 2025.*

Table of Contents

D1. Overview 2
Figure D1. Health-GPS model diagram 3
D2. Demographic module 3
D2.1. Data 3
D2.2. Births and deaths 3
D2.3. Residual mortality 4
D2.4. Population 5
D2.5. Net migration 5
D3. Risk factor modules 5
Figure D2. Energy balance model layers and factors, used within Health-GPS. 6
D3.1. Energy balance model 6
D3.2. Sodium intake 6
D3.3. Risk factor initialization and propagation 7
D4. Disease modules 8
D5. Burden of disease module 9
D6. Health expenditure 10
D7. Hypertension 11
Table D1. Model parameters and variables 12
References Appendix D 15

D1. Overview

Health-GPS is a microsimulation model that simulates individuals over time, estimating their risks of developing a variety of NCDs (e.g., stroke) given their exposure to particular risk factors (e.g., excessive sodium intake). Health-GPS then models possible health policies (e.g., changes in taxation on foods high in sodium), that might affect these risk factors, and therefore may impact the incidence and outcomes of NCDs at the population level.

The model creates a synthetic population of individuals that broadly reproduces demographic and socioeconomic characteristics of the population of a given country, or sub-national jurisdiction, and simulates individual life histories from birth to death. Because it simulates a population at the individual level, Health-GPS can capture heterogeneity in risk exposures, diseases, and multi-morbidity patterns. Correlations between risk factor exposures and between diseases are reflected in simulated populations. Health-GPS is calibrated to real-world data to capture relationships between variables while matching to estimated and projected demographic and epidemiological metrics from international databases.

In Health-GPS, a baseline scenario is simulated reflecting expected demographic and epidemiological changes over a time horizon in a given population, to match current demographic projections over that same time horizon. Intervention scenarios are then simulated, reflecting the introduction of health policies applied to targeted individuals and risk factors. Changes in risk exposures will, in turn, impact other risk factors, diseases, and mortality through the model equations. Outcomes simulated through the model typically include, but are not limited to, the prevalence of risk factors, the incidence of, and mortality from, NCDs, years of life lost (YLLs), years lived with disability (YLDs), disability-adjusted life years (DALYs), and life expectancy. Policy impacts are estimated as the difference in these outcomes between the baseline and intervention scenarios. Given the random variation in model outputs due to Health-GPS' stochastic nature, the model repeats these steps multiple times and averages the differences to obtain the effect of a given health policy. Another way of limiting the impact of stochastic variation on simulation results is to model large population samples.

This appendix is divided into the following sections describing **four key Health-GPS component modules**, illustrated in **Figure D1**:

1. **Demographic module:** births, deaths, immigration, population and socioeconomic status.
2. **Risk factor modules:** including energy balance and high dietary sodium intake in the version used for the study described in this paper.
3. **Disease modules:** we explicitly model the following diseases: asthma; chronic kidney disease; diabetes; ischemic heart disease; and stroke (including intracerebral haemorrhage, ischemic stroke, and subarachnoid haemorrhage). Other diseases are accounted for in a residual mortality aggregate.
4. **Disease burden module:** including metrics used to estimate population-level outcomes of interventions, specifically YLLs, YLDs, and DALYs.

In the last section, we additionally include sources and methods used to estimate the health expenditure impact of interventions.

Figure D1. Health-GPS model diagram

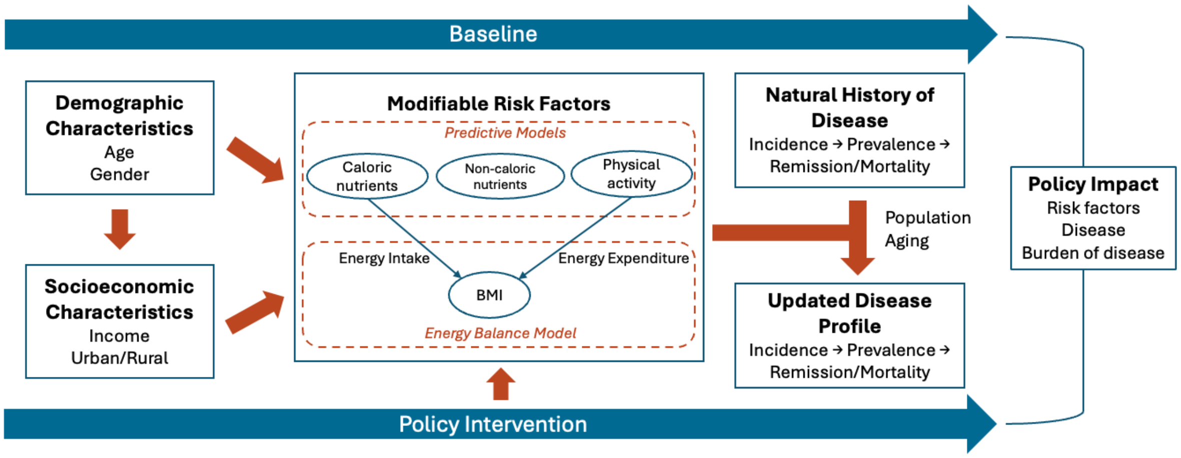

Notes: Disease is modelled through three types of events: onset, remission, and fatality, with data on prevalence, incidence, remission, and mortality rates for non-cancerous diseases collected from the IHME Global Burden of Disease. Cancers are modelled using data from the International Agency for Research on Cancer (IARC) on prevalence at 1, 3, and 5 years after diagnosis, incidence, and mortality rates. Cancer survivors beyond five years post-diagnosis are assumed to be in remission.

D2. Demographic module

The demographic module manages the yearly numbers of births, deaths, and migrations, as well as socioeconomic status. This module aims to produce a baseline scenario consistent with projected demographic data.

D2.1. Data

Health-GPS uses the United Nations (UN)'s World Population Prospects projections for yearly births, deaths, total population, and net migration, for all countries, by age and sex [1]. In the next sections, we explain how these data are calibrated in the demographic module.

D2.2. Births and deaths

The demographic module reproduces historical and projected births and deaths in a baseline scenario. The UN estimates and projects birth and death rates for all countries from 1950 until 2100. These estimates and projections are available in five-year intervals, and the module interpolates the data linearly to account for missing years.

These data determine the number of births at the beginning of each year of the simulation, assigning sex based on a sex ratio. Newborns are disease-free at the beginning of the simulation, and their initial risk factor exposures are generated using the initialisation equations of the risk factor modules. For all individuals in Health-GPS, risk and disease profiles are updated yearly until they either die or migrate.

Similarly, UN data provide estimates of the number of deaths in each year of the simulation. Health-GPS calculates an individual's probability of death each year based on their sex, age and disease profile. The model simulates multiple diseases but allows for death from other non-modelled causes, i.e. residual mortality.

D2.3. Residual mortality

Let the ith person (who has neither died nor emigrated) have age a_i and sex g_i. Let $m_{dag}(t)$ be the annual 'excess mortality' associated with disease d, for age a, and sex g, during calendar year t. We use the Institute for Health Metrics and Evaluation (IHME) definition of excess mortality [2], that is disease-specific mortality divided by disease prevalence. Then let $m_{id}^*(t)$ denote

$$m_{id}^*(t) = \begin{cases} m_{da_ig_i}(t) & \text{if person } i \text{ has disease } d \text{ at time } t \\ 0 & \text{otherwise} \end{cases}$$

where $0 \leq m_{id}^*(t) \leq 1$. Then the probability $s_i(t)$ that the ith person survives diseases $1, \dots, N_D$ in year t is given by

$$s_i(t) = \prod_{d=1}^{N_D} 1 - m_{id}^*(t)$$

Then the number of people $N_{sag}(t)$ of age a, and sex g who survive diseases $1, \dots, N_D$ in year t is given by

$$N_{sag}(t) = \sum_{\substack{i:a_i=a \\ i:\, g_i=g}} s_i(t)$$

and so the proportion of people $S_{ag}(t)$ of age a, and sex g who survive diseases $1, \dots, N_D$ in year t is given by

$$S_{ag}(t) = {N_{sag}(t)}\big/{N_{ag}(t)}$$

where N is the number of people in the population, and $N_{ag}(t)$ denotes the number of people of age a and sex g at time t.

If $M_{ag}(t)$ and $RM_{ag}(t)$ respectively denote the population-level mortality rate and residual mortality rate (of diseases *not* modelled in Health-GPS), for people of age a and sex g, then we have

$$1 - M_{ag}(t) = (1 - RM_{ag}(t))S_{ag}(t)$$

and so rearranging gives

$$RM_{ag}(t) = 1 - \left({(1 - M_{ag}(t))}\big/{S_{ag}(t)}\right)$$

This equation is used throughout the model. It is calculated in the baseline scenario, and the corresponding values are then used in the simulation of intervention scenarios. This assumes that mortality from non-modelled causes of death is constant across scenarios. However, diseases are not always assumed to be independent in Health-GPS, which means that variations in morbidity from modelled diseases that are produced by an intervention may impact non-modelled causes of death, and therefore residual mortality. Therefore, the assumption that residual mortality is constant across scenarios is an approximation and may lead to a (small) underestimation of the effects of an intervention when such an intervention produces a reduction in morbidity from modelled diseases.

At any given time *t*, each individual *i* is exposed to a specific risk of death in connection with the diseases they have, plus a residual risk in connection with causes of death that are not explicitly modelled in the simulation. Individuals who reach age 110 (i.e., the maximum attainable age in Health-GPS) are assumed to die at the next time step.

In the simulation, each year individuals die with probability $(1 - s_i(t))\ RM_{a_ig_i}(t)$.

D2.4. Population

The demographic module uses historical and projected population changes by year, sex, and age. UN population projections are available in 5-year age groups, and we linearly interpolate the population for each age group between two years. Similarly to deaths, population numbers are smoothed. This conserves the total annual population by sex.

D2.5. Net migration

While data on births and deaths are publicly available in several databases, finding accurate data on migration is more challenging. We model migration using the following formula:

$$TP(a+1,t+1) = TP(a,t) + B(t) - D(a,t) + I(a,t)$$

where $TP(a,\mathrm{t}), B(t), D(a,\mathrm{t})$ and $I(a,\mathrm{t})$ respectively denote the total population, number of births, number of deaths, and net migration for age a in year t.Therefore, migration is simulated indirectly to replicate expected numbers from the UN World Population Prospects database [1]. Consequently, depending on the sign of net migration, individuals will be added or removed from the population. In particular, the attributes of migrants are bootstrapped (i.e., sampled with replacement) from the distributions of the existing individuals of the same sex and age.

D3. Risk factor modules

Health-GPS models risk factor exposures accounting for hierarchical dependencies. For instance, characteristics of the living environment (e.g., neighbourhood deprivation) may influence individual behaviours (e.g., dietary intake), and this in turn, may influence the likelihood of conditions (e.g., obesity, hypertension) typically linked with the incidence of NCDs. Diseases themselves can be risk factors for other diseases. Diabetes, for example, is associated with a higher incidence of cardiovascular disease or chronic kidney disease [3,4]. The associations between risk factors are complex and dynamic. They can change due to exogenous shocks such as changes in food prices, or the implementation of new interventions and policies. As individuals age in the simulation, their risk exposures are updated in line with the latest observed age- and sex-specific prevalences (those used to initialise the synthetic population at the start of the simulation). Such age- and sex-specific prevalences are typically held constant throughout the projection period unless specific underlying trends are assumed (in some of the analyses in this project, for instance, a future trend in ultra-processed food consumption has been assumed). Future trends over time in risk factor exposures may apply uniformly for the entire population, or differently for different individuals or population sub-groups.

In the version of the Health-GPS model used for the evaluation of food tax policy scenarios in India, the most upstream risk exposure is food prices, which are assumed to influence food purchases and eventually nutrient intakes. All of the above risk exposures are modelled at the household level outside Health-GPS, using a separate demand system model, described in the paper. Individual nutrient intakes are calculated based on household food purchases and are used as inputs into Health-GPS microsimulations. Nutrient intakes are assumed to generate health impacts through two main pathways: energy and sodium. These two pathways are described in the following sections, 3.1 and 3.2.

Figure D2. Energy balance model layers and factors, used within Health-GPS.

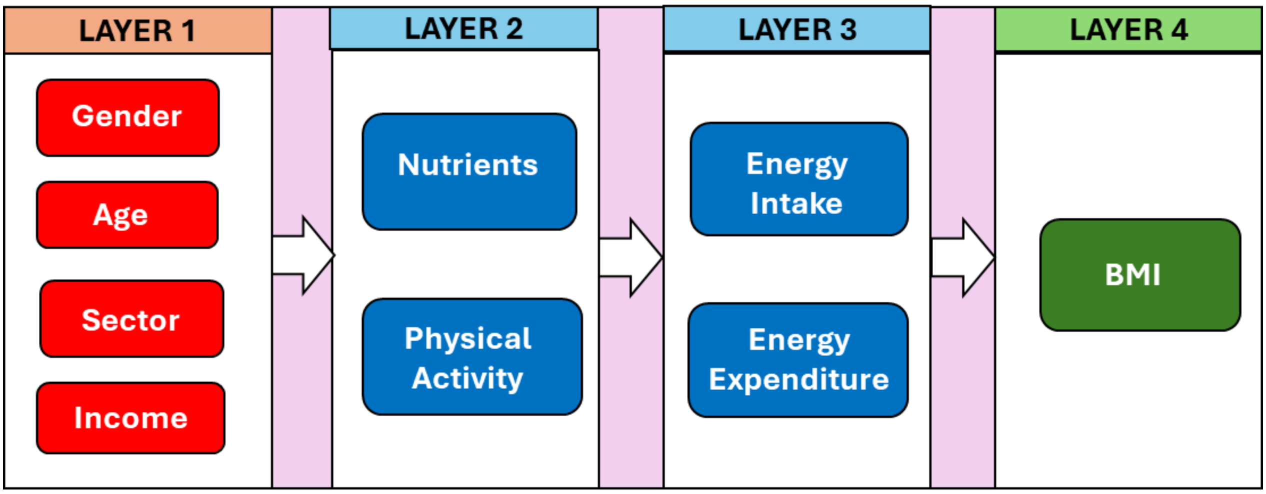

Notes: BMI: body mass index.

D3.1. Energy balance model

Health-GPS models body weight dynamics through an energy balance model (EBM) following the mathematical framework developed by Hall et al (2022) [5]. The purpose of this model is to understand and predict how various factors, such as diet and physical activity, influence an individual's body weight over time. In this section, we provide an overview of how Health-GPS incorporates the Hall et al (2022) model [5]. Full details can be found in Hall et al (2022) [5] and all Health-GPS model codes (including the implementation of the Hall et al (2022) model) can be found at https://github.com/imperialCHEPI/healthgps .

The Hall et al (2022) model takes into account the principles of energy balance, which is the relationship between the calories consumed through food and beverages and the calories expended through metabolism and physical activity [5]. The model incorporates complex physiological processes and feedback mechanisms to provide a more realistic representation of weight dynamics than simpler, linear models.

In this framework, energy intake and expenditure are the result of diet, physical activity and metabolic patterns. Body weight changes in the microsimulation are associated with dynamic imbalances between energy intake (EI, the calories consumed from food and beverages), and energy expenditure (EE, the energy expended throughout the day). When an individual is in a steady state energy balance (i.e., "energy in" equals "energy out"), body weight is assumed to remain stable. A surplus of energy is stored as fat, leading to weight gain. Conversely, an energy deficit leads to weight loss.

Energy intake and expenditure are determined by nutrient intakes (fat, carbohydrates, protein), physical activity, and prior body mass index (BMI) as a proxy for metabolic energy requirements, which in turn are determined by individual characteristics including age, sex and socioeconomic status (**Figure D2**). The output of the energy balance model is a (time-dependent) change in BMI.

Each layer in **Figure D2** influences subsequent layers. Risk factors are simulated in two steps: 1) initialisation, using static risk factor equations; 2) projection, which updates values for each risk factor at every time cycle during the simulation using dynamic risk factor equations.

D3.2. Sodium intake

Sodium intake is modelled alongside nutrient intake that contribute to the EBM. Sodium intake affects disease incidence directly, through relative risks derived from existing studies. Most of the sodium-attributable risk of disease is generated through an increase in blood pressure caused by excessive sodium intakes, however, the individual steps of this causal chain are not modelled in Health-GPS. The relative risks used to model increases in disease risk linked with sodium intake account for the increased blood pressure that mediates the effect of sodium intake on diseases. On the other hand, changes in hypertension prevalence are calculated in parallel as a function of sodium intake.

D3.3. Risk factor initialization and propagation

We use a two-stage approach to initialize risk factors within each person in Health-GPS that combines regression modelling, data transformation, and stochastic variation.

Nutrient initialisation

First, we use a regression model to determine average values of nutrient intake by age, sex, income (i.e., high, middle and low) and sector (i.e., rural or urban). Second, for each person and each nutrient, we add a stochastic variation term to these average values to generate the required population heterogeneity. This stochastic variation term is sampled from the residuals of the regression performed in the first step.

To ensure normality of the nutrient intake variables, a Box-Cox transformation is applied:

$$\acute{y}_k = \begin{cases} \dfrac{y_k^{\lambda} - 1}{\lambda_k} & , \lambda_k \neq 0 \\ ln(y_k) & , \lambda_k = 0 \end{cases}$$

where $\acute{y}_k$ is the transformed variable, y_k is the dependent variable (nutrient intake) and λ_k is the transformation variable for nutrient $k = 1, \dots, 4$.

After transforming the variables, average daily nutrient intake $\acute{y}_k$ is initialized using the equation below for each of the following four nutrients: fat, protein and carbohydrates (measure in grams per day); and sodium (measured in milligrams per day). For nutrient k, we have

$$\acute{y}_k = \beta_{k0} + \beta_{k1}.\text{Sex} + \beta_{k2}.\text{Age} + \beta_{k3}.\text{Age}^2 + \beta_{k4}.\text{Age}^3 + \beta_{k5}.\text{Sector} + \beta_{k6}.\text{Income} + \varepsilon_k$$

where β_{k0} is the intercept and ε_k is the error term for nutrient k. Data input for socio-economic variables and nutrient intake are based on NSS 68th round 2011-2012 and adjusted to December 2022 using the methods detailed in the Methods section of the paper.

To capture realistic variation and maintain correlations between nutrients, stochastic residuals (ε) are generated using Cholesky decomposition.

Energy intake and energy expenditure initialisation

After daily nutrient intake values have been assigned for each person, they are inputted into the Hall et al (2022) model,[5] together with each person's Physical Activity Level (PAL), to determine each person's energy intake (EI), and energy expenditure (EE). Physical Activity Level (PAL) is the ratio between energy expended through physical activity and the person's baseline metabolic rate. Individual PAL values are initialized from a normal distribution

$$PAL \sim Normal(1.6, 0.06)$$

and subsequently adjusted to maintain population-level expected values. Energy expenditure (EE) is calculated as a function of body composition and several metabolic parameters, integrating multiple physiological processes, including resting metabolic rate, thermic effect of food, adaptive thermogenesis, and energy partitioning. Further details can be found in Hall et al (2022) [5].

Energy intake (EI) quantifies the total caloric content consumed and is a function of the daily nutrient intakes calculated above.

BMI calculation

Body Mass Index (BMI) is calculated using the standard definition

$$BMI_i(t) = \frac{W_i(t)}{(H_i(t))^2}$$

where $W_i(t)$ and $H_i(t)$ respectively denote individual i's weight and height at time t.

$W_i(t)$ is initialized as a function of age, sex, energy intake and physical activity. H_i is calculated based on population-level expected values given a person's age and sex, their weight, as well as randomly sampled individual variation. Height is assumed to remain constant after age 19. Individual BMI values are calibrated to the BMI distribution for India in the NCD-RisC database through an adjustment of weight and nutrient intake values [6].

Using the initialized values for weight, height, energy intake and physical activity, the Hall et al (2022) model is used to initialize values of body fat, lean tissue, glycogen, extracellular fluid and energy expenditure for each person [5]. The changes in these values (governed by changes in nutrient intake and energy intake) are then used to update each individual's weight, and thus their BMI.

D4. Disease modules

Health-GPS is calibrated to match empirical disease distributions. Changes in the distributions of risk factors and diseases are then transferred to other diseases. Although we assume the associations of risk factors to diseases to be constant throughout the simulation, any deviation of a risk factor from its initial distribution will translate into increased or decreased disease incidence through relative risk equations (the epidemiological literature does not always distinguish clearly between relative risks and hazard ratios; when it is not possible to identify values for both, available values are used interchangeably in Health-GPS). Therefore, any change in the prevalence of a disease is solely caused by changes in risk factor distributions, alongside the ageing of the population.

The disease module uses multiple data sources, including IHME for prevalence, incidence, mortality and remission rates for non-cancerous diseases [2]. When cancers are modelled (which is not the case in this study), Health-GPS uses data from the International Agency for Research on Cancer (IARC) for prevalence at 1, 3 and 5 years (from diagnosis), incidence, and mortality rates [7]. Relative risks from BMI to diseases are derived from IHME attributable risk data, while those for sodium to ischaemic heart disease, stroke, and chronic kidney disease are derived from specific studies [8,9].

Probabilities of disease for each individual are updated throughout the simulation to reflect changes in risk exposures and disease profiles. Prevalence in the initial population is used to calibrate Health-GPS parameters. However, as time progresses, risk factor distributions and demographics are the sole drivers of prevalence.

The excess mortality rate is the proportion of excess deaths within a designated population of "cases" (people with a medical condition) over the course of the disease. These rates are kept constant over time in the simulation. Remission is the rate at which people with a disease revert to a state with the same survival prospect and disability level as those without the disease. Remission rates are kept constant over time in Health-GPS. In this study, remission rates are taken from IHME [2]. When not available, they are assumed zero.

Disease is modelled through 3 types of events: onset, remission and fatality. An individual's probability of acquiring a disease is calculated based on their risk exposures, existing conditions, and the population-level incidence of each disease. Therefore, this incidence is adjusted for each individual to account for their sex, age, risk and disease profiles. For each individual in the population and for each disease, we compute an Individual

Relative Risk (IRR), which reflects the aggregate change in disease incidence associated with the individual's risk factors and diseases. After disease onset, the individual may either go into remission or die, over time.

Let RR_{dfag} be the relative risk of disease d associated with risk factor $f = 1, \ldots, N_f$ for age a and sex g. Let DR_{dagj} be the relative risk of disease d associated with the presence of another disease j (where $j = 1, \ldots, N_D$). for age a and sex g. If

$$RR^*_{id}(t) = \begin{cases} RR_{dfa_ig_i} & \text{if person } i \text{ has risk factor f at time } t \\ 1 & \text{otherwise} \end{cases}$$

and

$$DR^*_{idj}(t) = \begin{cases} DR_{dja_ig_i} & \text{if person } i \text{ has disease } j \text{ at time } t \\ 1 & \text{otherwise} \end{cases}$$

then individual i's overall relative risk $IRR_{id}(\mathrm{t})$ of disease d is given by

$$IRR_{id}(t) = \prod_{f=1}^{N_f} RR^*_{id}(t). \prod_{j=1}^{N_D} DR^*_{idj}\,(t)$$

At the beginning of the simulation, each individual's disease status is assigned based on: (a) their $IRR_{id}(t_0)$; (b) the mean value of this quantity among people of the same age and sex; and (c) the prevalence of disease d in the same age and sex group. Therefore, individual i is assigned disease d with probability equal to

$$\frac{IRR_{id}(t_0)}{|IRR_d|_{a_ig_i}(t_0)} \times P_{da_ig_i}(t_0)$$

where $P_{dag}(t_0)$ and $|IRR_d|_{ag}$ respectively denote the population level prevalence, and the average relative risk of disease d for age a and sex g, at the beginning of the simulation. Note that this formulation assumes relative risks combine multiplicatively and are independent.

Similarly, if $I_{dag}(t_0)$ denotes the population level incidence of disease d for age a and sex g, as currently observed empirically (at t_0), then individual i develops disease d at time t with probability equal to

$$IRR_{id}(t) \times I^*$$

where

$$I^* = \frac{I_{da_ig_i}(t_0)}{|IRR_{id}|_{a_ig_i}(t_0)}$$

I^* is calculated in the baseline scenario and is held constant throughout the simulation. It is also used throughout the simulation of intervention scenarios.

Finally, each person of age a and sex g with disease d has probability ρ_{dag} of going into remission each year, and dies with probability $(1 - s_i(t))\; RM_{a_ig_i}(t)$ each year (see above section on Residual Mortality).

D5. Burden of disease module

If DW_d represents the disability weight associated with disease d, let

$$DW^*_{id}(t) = \begin{cases} DW_d & \text{if person } i \text{ has disease } d \text{ at time } t \\ 0 & \text{otherwise} \end{cases}$$

Now define the product $X_i(t)$ of the compliment of disability weights $X_i(t)$ by

$$X_i(t) = \prod_{d=1}^{N_D} (1 - DW^*_{id}(t))$$

and the average of this product for age a and sex g is

$$|X|_{ag}(t) = \sum_{\substack{i:a_i=a \\ i:g_i=g}} X_i(t) \Big/ N_{ag}(t)$$

where $N_{ag}(t)$ denotes the number of people of age a and sex g at time t. If $YLD_{ag}(0)$ is the empirically observed years lived with disability for age a and sex g over all diseases at the time of initialization, and if RDW_{ag} is the residual disability weight for diseases *not* included in Health-GPS for age a and sex g, then at time $t = 0$, we have

$$(1 - YLD_{ag}(0)) = (1 - RDW_{ag})|X|_{ag}(0)$$

Rearranging gives

$$RDW_{ag} = 1 - \left((1 - YLD_{ag}(0)) \Big/ |X|_{ag}(0)\right)$$

RDW_{ag} is initialized as above at the beginning of the simulation and unchanged after. More generally, across diseases (modelled in Health-GPS or otherwise) at time t, each person i has disability weight $DW_i(t)$ given by

$$DW_i(t) = 1 - (X_i(t)(1 - RDW_{ag}))$$

Disability-adjusted life years ($\text{DALYs}_{ag}(t)$) are calculated as the sum of the years lost due to premature mortality ($YLL_{ag}(t)$) and the years lived with a disability $YLD_{ag}(t)$ over the time horizon of the simulation, for age a and sex g. $YLD(t)$ at time t is given by

$$YLD_{ag}(t) = \frac{100{,}000}{N_{ag}(t)} \sum_{\substack{i:a_i=a \\ i:g_i=g}} DW_i(t)$$

The contribution of person i to years of life lost at time t is given by

$$YLL_i(t) = \begin{cases} LE(t) - a_i & \text{if } TD_i = t \text{ and } LE(t) > a_i \\ 0 & \text{otherwise} \end{cases}$$

where a_i is their age, TD_i gives their time of death, and $LE(t) = \max\{LE_{\text{Male}}(t), LE_{\text{Fenale}}(t)\}$ is the life expectancy at time t. Then

$$YLL_{ag}(t) = \frac{100{,}000}{N_{ag}(t)} \sum_{\substack{i:a_i=a \\ i:g_i=g}} YLL_i(t)$$

The DALYs at time t are then given by

$$\text{DALYs}_{ag}(t) = YLL_{ag}(t) + YLD_{ag}(t)$$

D6. Health expenditure

Total health expenditure (THE) consists of current health expenditure (CHE) and capital expenditure. According to National Health Accounts Estimates for India 2021-22, CHE is 87.32% of THE, and of the CHE, governments' share is 38.79% and the households' share is 50.55% (including out-of-pocket expenditure and insurance contributions). Out-of-pocket expenditure (OOPE) specifically account for 45.11% of THE [10]. Therefore, households' expenditure and households' OOPE on health accounts for 44.14% and 39.39% of THE, respectively.

For each disease in asthma, chronic kidney disease, diabetes, ischemic heart disease, and stroke (including ischemic stroke, intracerebral haemorrhage, and subarachnoid haemorrhage), either households' expenditure, or

OOPE on health, per person per year in India is derived from previous studies [11-13], depending on availability, and is then converted into THE per case following the method adopted by WHO in NCD investment cases [14]. Finally, the averted annual THE of each disease under intervention is calculated as the product of reduced cases under intervention and THE per case.

D7. Hypertension

Changes in hypertension prevalence are estimated independently with data on hypertension prevalence in India from the National Family Health Survey (NFHS-5), 2019-21 [15], estimates on changes in sodium and BMI under intervention from Health-GPS, and the association between sodium, BMI and the risk of hypertension from literature [16,17].

A linear association is assumed between BMI and the risk of hypertension, and between sodium and the risk of hypertension. One unit change in sodium (mg) and BMI (kg/m^2) is associated with a change of 0.00003 and 0.186 in the risk of hypertension, respectively [16,17]. Reduction in prevalence number of hypertension by age and sex is calculated as follows.

$$\Delta hypertension_{a,s} = prevalence_{a,s} \times \left[1 - (1 - \Delta sodium_{a,s} \times slope_{sodium}) \times (1 - \Delta bmi_{a,s} \times slope_{bmi})\right]$$

Where a denotes age and s denotes sex. $prevalence_{a,s}$ is the baseline prevalence number of hypertension from NFHS-5. $\Delta sodium_{a,s}$ and $\Delta bmi_{a,s}$ are policy effect estimates from Health-GPS. $slope_{sodium} = 0.00003$. $slope_{bmi} = 0.186$.

Table D1. Model parameters and variables

Symbol	Definition	Source/Assumption
Indices		
i	Index of person	
a	age	0-100
g	Index of sex	Male/Female
d	Index of disease	
k	Index of nutrient	
j	Index of disease other than disease d	
l	Index of covariate	
Demographic module		
t	Calendar time (year)	2022-2053 (policy introduced in 2024)
$m_{dag}(t)$	Annual 'excess mortality' associated with disease d, for age a, and sex g, during calendar year t	IHME [2]
N_D	Number of diseases simulated	7 in this study including asthma, chronic kidney disease, diabetes, ischemic heart disease, and stroke (including ischemic stroke, intracerebral haemorrhage, and subarachnoid haemorrhage)
$s_i(t)$	Probability that the ith person survives all simulated diseases in year t	Calculated in Health-GPS
$N_{sag}(t)$	Number of people of age a, and sex g who survive all simulated diseases in year t	Calculated in Health-GPS
$S_{ag}(t)$	Proportion of people of age a, and sex g who survive all simulated diseases in year t	Calculated in Health-GPS
$N_{ag}(t)$	Number of people of age a and sex g at time t.	UN World Population Prospects database [1]
$M_{ag}(t)$	Age- and sex-specific population-level mortality rate in year t	UN World Population Prospects database [1]
$RM_{ag}(t)$	Age- and sex-specific population-level residual mortality rate (of diseases not modelled in Health-GPS) in year t	Calculated in Health-GPS
$TP(a,t)$	Total population of age a in year t	UN World Population Prospects database [1]
$B(t)$	Births in year t	UN World Population Prospects database [1]
$D(a,t)$	Deaths of age a in year t	UN World Population Prospects database [1]
$I(a,t)$	Net migration for age a in year t	UN World Population Prospects database [1]
$income_i(t)$	Income tertile of individual i in year t	NSS Household Consumption Expenditure survey 2022-23
$sector_i(t)$	Sector of residence (urban or rural) of individual i in year t	NSS Household Consumption Expenditure survey 2022-23

Risk factor module		
y_1	Value of nutrient 1: Carbohydrate (untransformed)	NSS Household Consumption Expenditure survey 2022-23
y_2	Value of nutrient 2: Protein (untransformed)	NSS Household Consumption Expenditure survey 2022-23
y_3	Value of nutrient 3: Fat (untransformed)	NSS Household Consumption Expenditure survey 2022-23
y_4	Value of nutrient 4: Sodium (untransformed)	NSS Household Consumption Expenditure survey 2022-23
$\acute{y}_k$	Box-Cox transformed value of nutrient k	Calculated in Health-GPS
λ_k	Box-Cox transformation parameter for nutrient k	Calculated with y_k
β_{kl}	Linear regression coefficient for nutrient k and covariate l	Calculated with $\acute{y}_k$, a, g, $income_i(t)$ and $sector_i(t)$
ε_k	Error term for nutrient k	Calculated with $\acute{y}_k$, a, g, $income_i(t)$, $sector_i(t)$ and β_{kl}
σ_k	Standard deviation of ε_k	Calculated with ε_k
PAL	Physical Activity Level	A normal distribution with mean as 1.6 (i.e., 1.6 times basic metabolic requirement) and standard deviation as 0.06
$W_i(t)$	Weight of individual i at time t	Distribution from NCD-RisC [6]
$H_i(t)$	Height of individual i at time t	Distribution by age and sex from NCD-RisC [6]
$slope_g$	Slope of height over weight for sex g	Female: 0.3564; Male: 0.3718; estimated based on NCD-RisC data [6]
std_g	Standard deviation of height for sex g	Female: 0.096; Male: 0.085; estimated based on NCD-RisC data [6]
$BMI_i(t)$	Body Mass Index of individual i at time t	Calculated with $W_i(t)$ and $H_i(t)$
Disease module		
RR_{dfag}	Age- and sex-specific relative risk of disease d associated with risk factor f	IHME [2] and literature [8-9]
N_f	Number of dietary risk factors simulated	Given by authors, 4 in this study (carbohydrates, protein, fat, and sodium)
DR_{dagj}	Age- and sex-specific relative risk of disease d associated with presence of another disease j	IHME [2] and literature [18-19]
$IRR_{id}(\mathrm{t})$	Individual i's overall relative risk $IRR_{id}(\mathrm{t})$ of disease d at time t	Calculated in Health-GPS
$P_{dag}(t_0)$	Age- and sex-specific population level prevalence of disease d at the beginning of the simulation	IHME [2]
ρ_{dag}	Age- and sex-specific probability of going into remission from disease d each year	IHME [2]
Burden of disease module		
DW_d	Disability weight associated with disease d	IHME [2]

RDW_{ag}	Age- and sex-specific residual disability weight for diseases *not* included in Health-GPS	Calculated in Health-GPS
$YLL_{ag}(t)$	Age- and sex-specific years lost due to premature mortality over the time horizon of the simulation	Calculated in Health-GPS
$YLD_{ag}(t)$	Age- and sex-specific years lived with a disability over the time horizon of the simulation	Calculated in Health-GPS
$YLL_i(t)$	Contribution of person i to years of life lost at time t	Calculated in Health-GPS
TD_i	Time of death of person i	Calculated in Health-GPS
$LE(t)$	Life expectancy at time t	Calculated in Health-GPS
$\text{DALYs}_{ag}(t)$	Age- and sex-specific disability-adjusted life years at time t	Calculated in Health-GPS

References Appendix D

1 United Nations, Department of Economic and Social Affairs, Population Division. World Population Prospects 2022 [Internet]. New York: United Nations; 2022 [cited 2023 Nov 26]. Available from: https://population.un.org/wpp/

2 Institute for Health Metrics and Evaluation. Global Burden of Disease [Internet]. Seattle (WA): IHME, University of Washington; [cited 2023 Nov 20]. Available from: https://www.healthdata.org/research-analysis/gbd

3 Matheus AS, Tannus LR, Cobas RA, Palma CC, Negrato CA, Gomes MD. Impact of diabetes on cardiovascular disease: an update. International Journal of Hypertension. 2013;2013(1):653789.

4 Koye DN, Magliano DJ, Nelson RG, Pavkov ME. The global epidemiology of diabetes and kidney disease. Advances in Chronic Kidney Disease. 2018 Mar 1;25(2):121–32

5 Hall KD, Sacks G, Chandramohan D, Chow CC, Wang YC, Gortmaker SL, Swinburn BA. Quantification of the effect of energy imbalance on bodyweight. The Lancet. 2011 Aug 27;378(9793):826–37.

6 Institute for Health Metrics and Evaluation. Global Burden of Disease [Internet]. Seattle (WA): IHME, University of Washington; [cited 2023 Nov 20]. Available from: https://www.healthdata.org/research-analysis/gbd

7 International Agency for Research on Cancer. Global Cancer Observatory [Internet]. Lyon: IARC; 2022 [cited 2023 Nov 10]. Available from: https://gco.iarc.who.int/en

8 Tuomilehto J, Jousilahti P, Rastenyte D, Moltchanov V, Tanskanen A, Pietinen P, Nissinen A. Urinary sodium excretion and cardiovascular mortality in Finland: a prospective study. The Lancet. 2001 Mar 17;357(9259):848–51.

9 Liu N, Sun W, Xing Z, Ma F, Sun T, Wu H, Dong Y, Xu Z, Fu Y, Yuan H. Association between sodium intakes with the risk of chronic kidney disease: evidence from a meta-analysis. International Journal of Clinical and Experimental Medicine. 2015;8(11):20939.

10 Ministry of Health and Family Welfare, Government of India. National Health Accounts: estimates for India 2021–22 [Internet]. New Delhi: MoHFW; 2024 [cited 2024 Sep 25]. Available from: https://nhsrcindia.org/sites/default/files/2024-09/NHA 2021-22.pdf

11 Yadav J, Menon GR, John D. Disease-specific out-of-pocket payments, catastrophic health expenditure and impoverishment effects in India: an analysis of National Health Survey data. Applied Health Economics and Health Policy. 2021 Sep;19:769–82.

12 Kwatra G, Kaur P, Toor G, Badyal DK, Kaur R, Singh Y, Pandian JD. Cost of stroke from a tertiary center in northwest India. Neurology India. 2013 Nov 1;61(6):627–32.

13 Kansra P, Oberoi S. Cost of diabetes and its complications: results from a STEPS survey in Punjab, India. Global Health Research and Policy. 2023 Apr 7;8(1):11.

14 Pan American Health Organization; World Health Organization; United Nations Inter-Agency Task Force; United Nations Development Programme. The case for investment in prevention and control of noncommunicable diseases and mental disorders in Suriname: a return-on-investment analysis [Internet]. Washington (DC): PAHO; 2023 [cited 2025 Aug 9]. Available from: https://www.paho.org/en/node/93097

15 International Institute for Population Sciences (IIPS) and ICF. 2021. National Family Health Survey (NFHS-5), 2019-21: India: Volume I [Internet]. Mumbai: IIPS [cited 2025 Aug 9]. Available from: https://dhsprogram.com/pubs/pdf/FR375/FR375.pdf

16 Gelber RP, Gaziano JM, Manson JE, Buring JE, Sesso HD. A prospective study of body mass index and the risk of developing hypertension in men. Am J Hypertens. 2007 Apr;20(4):370-7.

17 Filippini T, Malavolti M, Whelton PK, Vinceti M. Sodium Intake and Risk of Hypertension: A Systematic Review and Dose-Response Meta-analysis of Observational Cohort Studies. Curr Hypertens Rep. 2022 May;24(5):133-144.

18 Almdal T, Scharling H, Jensen JS, Vestergaard H. The independent effect of type 2 diabetes mellitus on ischemic heart disease, stroke, and death: A population-based study of 13 000 men and women with 20 years of follow-up. Arch Intern Med. 2004;164(13):1422–6.

19 Mueller NT, Koh WP, Odegaard AO, Gross MD, Yuan JM, Pereira MA. Asthma and the risk of type 2 diabetes in the Singapore Chinese Health Study. Diabetes Res Clin Pract [Internet]. 2013 Feb [cited 2025 Sep 15];99(2):192–9. Available from: https://pubmed.ncbi.nlm.nih.gov/23260853/
