# Supplementary material for: Taxation of foods high in fat, sugar, and sodium in India: A modelling study of health and economic impacts
Source: PLoS Med. 2026 Jan 5;23(1):e1004572. doi: 10.1371/journal.pmed.1004572 (PMC12768244; doi:10.1371/journal.pmed.1004572)
Supplement: S5 Text — Income trend assumption. (PDF) [file pmed.1004572.s005.pdf]

S5 Text

Appendix E. Income trend assumption

Appendix E forms part of the revised submission.

Supplement to: *Roche M, Zhu J, Olney J, Laydon DJ, Joe W, Sharma M, Steele L, Sassi F. Taxation of foods high in saturated fat, sugar, and sodium in India: A modelling study of health and economic impacts. Submitted after final revisions on 12 December 2025.*

Table of Contents

Description 2
Figure E1. Comparing projection assumptions for highly processed food items 3
References Appendix E 3

Description

The income trend factor $f_{i,j,0}$ for income group *i* and food group *j* for period $t = 0$ is estimated as the product of the income elasticity estimate ($\eta_{i,j}$) (**Table C1**) and an income growth assumption. We assume a constant 6% income growth for India based on IMF projections [2].

$$f_{i,j,0} = 1 + 0.06 \times \eta_{i,j}$$

Food consumption for period $t = 1$ is then estimated at the food item level by multiplying $f_{i,j,0}$ with the household level total daily consumption at $t = 0$. We derive household level daily intake of energy and nutrient by multiplying the estimated daily consumption for each item at $t = 1$ with each item's energy and nutrient content derived from food composition tables. We sum this up at household-level to obtain the estimated household daily total intake of energy and nutrient. We then individualise daily intake based on household members' age and sex using the daily NIN Dietary Guidelines for Indians 2024 to obtain the estimated daily individual nutrient intake ($\hat{q}_{i,n,1}$) for individual *i*, nutrient *n*, and period $t = 1$. The baseline individual-level nutrient-specific income trend factor $f_{i,n,0}$ for individual *i* and nutrient *n* is recovered as below:

$$f_{i,n,0} = 1 + \frac{\hat{q}_{i,n,1} - q_{i,n,0}}{q_{i,n,0}}$$

We then run the below cross-sectional OLS regression:

$$\log(\mathrm{q_{i,n,0}}) = f\left(\log(MPCE_{i,h,0}), \mathbf{X_{i,h,0}}, \boldsymbol{\beta}\right)$$

Where $MPCE_{i,h}$ represents the per capita monthly total expenditure for household h and $\mathbf{X_{i,h}}$ a vector of household and individual characteristics including the household sector, size, religion, and the individual sex, age, and education. We obtain the vector of coefficients $\hat{\boldsymbol{\beta}}$.

We recover the predicted individual level nutrient intake for future periods as:

$$\hat{\mathrm{q}}_{\mathrm{i,n,t}} = e^{f\left(\log(MPCE_{i,h,t}), \mathbf{X_{i,h,0}}, \hat{\boldsymbol{\beta}}\right)}$$

Where $MPCE_{i,h,t} = MPCE_{i,h,0} \times 1.06^t$ based on the constant IMF projection assumption for real GDP per capita.

The nutrient-specific decay rate $\hat{b}_n$ is finally recovered as the coefficient from the below OLS regression of the logarithm of the predicted growth rate in nutrient intake on a time trend:

$$\log\left(\frac{\hat{\mathrm{q}}_{\mathrm{i,n,t+1}}}{\hat{\mathrm{q}}_{\mathrm{i,n,t}}}\right) = g(t, b_n)$$

As expected from Engel's Law, which posits that, as household income increases, the proportion spent on food decreases, $b_n < 0$. To align with Engel's Law [2], the individual-level nutrient-specific income trend factor for period *t* is scaled by an exponential decay over time and is recovered using the below formulae:

$$f_{i,n,t} = f_{i,n,0} \times e^{b_n t}$$

Figure C1 shows that our income elasticities are well-behaved, i.e., higher for low-income households across food groups, in line with Engel's Law [2]. **Figure E1** cross-validates this approach by comparing the estimated yearly growth rate in the consumption of packaged processed foods and sweets - two food groups with a high

proportion of HFSS foods (**Table A6**) - with historical trends based on Euromonitor International Passport data and Tak et al (2022) for selected highly processed food items [3,4].

Figure E1. Comparing projection assumptions for highly processed food items

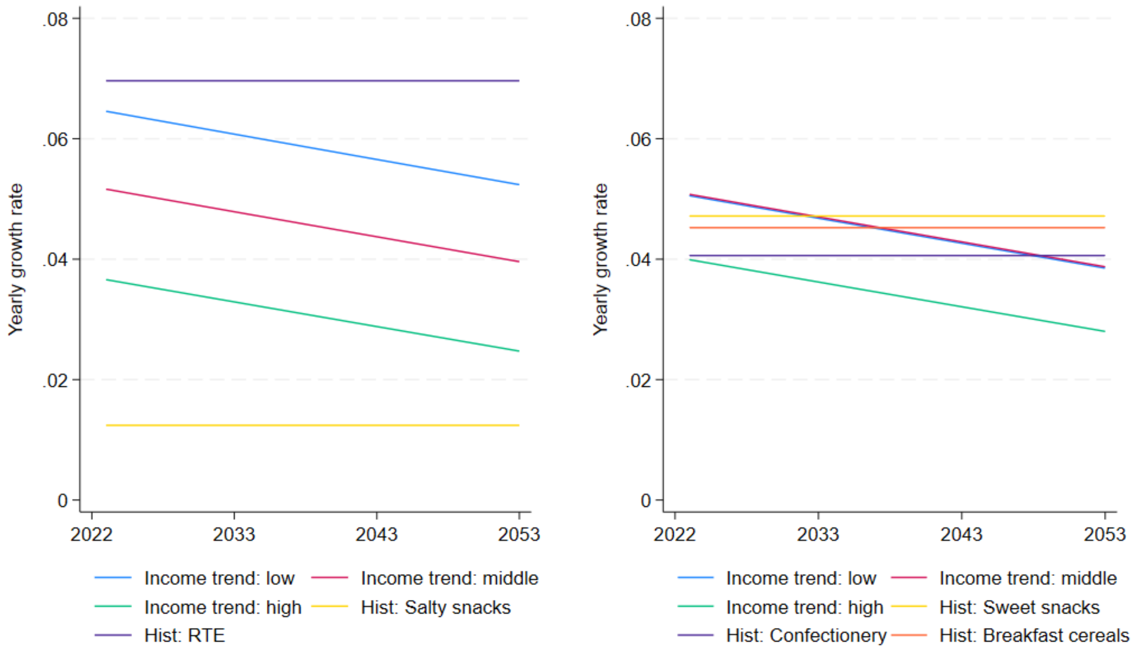

Notes: Historical trends based on Euromonitor International Passport data and Tak et al (2022) for selected highly processed food items [3,4]. Income group categorized using NSS Household Consumption Expenditure Survey 2022-23 monthly total household expenditure per capita. Hist: historial, RTE: ready-to-eat.

References Appendix E

1 International Monetary Fund. Countries: India [Internet]. Washington (DC): IMF; [cited 2025 May 20]. Available from: https://www.imf.org/en/Countries/IND

2 Engel E. Die Productions und Consumptionsverhältnisse des Königreichs Sachsen. Zeitschrift des Statistischen Bureaus des Königlich Sächsischen Ministerium des Inneren. 1857. Reprinted in: Engel E. Die Lebenskosten belgischer Arbeiter-Familien. Dresden: C Heinrich; 1895. German

3 Euromonitor International. Passport global market information database [Internet]. London: Euromonitor International; [cited 2025 Aug 6]. Available from: https://www.euromonitor.com/our-expertise/passport

4 Tak M, Law C, Green R, Shankar B, Cornelsen L. Processed foods purchase profiles in urban India in 2013 and 2016: a cluster and multivariate analysis. BMJ open. 2022 Oct 1;12(10):e062254
